# Impact of intensity standardisation and ComBat batch size on clinical-radiomic prognostic models performance in a multi-centre study of patients with glioblastoma

**DOI:** 10.1007/s00330-024-11168-7

**Published:** 2024-11-28

**Authors:** Kavi Fatania, Russell Frood, Hitesh Mistry, Susan C. Short, James O’Connor, Andrew F. Scarsbrook, Stuart Currie

**Affiliations:** 1 Department of Radiology, Leeds Teaching Hospitals NHS Trust, England, UK; 2 Leeds Institute of Medical Research, University of Leeds, Leeds, UK; 3 Division of Cancer Sciences, University of Manchester, Manchester, UK; 4 Department of Oncology, Leeds Teaching Hospitals NHS Trust, England, UK; 5 Department of Radiology, The Christie Hospital, Manchester, UK; 6 Division of Radiotherapy and Imaging, Institute of Cancer Research, London, UK

**Keywords:** Radiomics, Brain neoplasms, Diagnostic imaging, Glioblastoma, Prognosis

## Abstract

**Purpose:**

To assess the effect of different intensity standardisation techniques (ISTs) and ComBat batch sizes on radiomics survival model performance and stability in a heterogenous, multi-centre cohort of patients with glioblastoma (GBM).

**Methods:**

Multi-centre pre-operative MRI acquired between 2014 and 2020 in patients with IDH-wildtype unifocal WHO grade 4 GBM were retrospectively evaluated. WhiteStripe (WS), Nyul histogram matching (HM), and *Z*-score (ZS) ISTs were applied before radiomic feature (RF) extraction. RFs were realigned using ComBat and minimum batch size (MBS) of 5, 10, or 15 patients. Cox proportional hazards models for overall survival (OS) prediction were produced using five different selection strategies and the impact of IST and MBS was evaluated using bootstrapping. Calibration, discrimination, relative explained variation, and model fit were assessed. Instability was evaluated using 95% confidence intervals (95% CIs), feature selection frequency and calibration curves across the bootstrap resamples.

**Results:**

One hundred ninety-five patients were included. Median OS = 13 (95% CI: 12–14) months. Twelve to fourteen unique MRI protocols were used per MRI sequence. HM and WS produced the highest relative increase in model discrimination, explained variation and model fit but IST choice did not greatly impact on stability, nor calibration. Larger ComBat batches improved discrimination, model fit, and explained variation but higher MBS (reduced sample size) reduced stability (across all performance metrics) and reduced calibration accuracy.

**Conclusion:**

Heterogenous, real-world GBM data poses a challenge to the reproducibility of radiomics. ComBat generally improved model performance as MBS increased but reduced stability and calibration. HM and WS tended to improve model performance.

**Key Points:**

***Question*** *ComBat harmonisation of RFs and intensity standardisation of MRI have not been thoroughly evaluated in multicentre, heterogeneous GBM data*.

***Findings*** The *addition of ComBat and ISTs can improve discrimination, relative model fit, and explained variance but degrades the calibration and stability of survival models*.

***Clinical relevance*** *Radiomics risk prediction models in real-world, multicentre contexts could be improved by ComBat and ISTs, however, this degrades calibration and prediction stability and this must be thoroughly investigated before patients can be accurately separated into different risk groups*.

## Introduction

Glioblastoma (GBM) is the most common primary brain malignancy in adults, with a median overall survival (OS) of 12–15 months despite maximal oncological treatment (maximum safe surgical resection followed by adjuvant radiotherapy with concurrent temozolomide and further 6 cycles of adjuvant temozolomide—the Stupp protocol) [1, 2]. Many published models aim to improve risk stratification and help move towards developing ‘personalised medicine’ in GBM [3].

Extraction and analysis of large quantities of radiomic features (RFs) from medical imaging [4], have been used in prognostic models with promising results [5, 6]. However, clinical translation has been hampered by a lack of reproducibility linked to variability in multi-centre imaging protocols [7–9]. Intensity standardisation (IS) conforms to the scale and distribution of magnetic resonance imaging (MRI) signal intensity, which is affected by imaging protocol [10], however, there is no consensus on the best intensity standardisation technique (IST) [11, 12].

Statistical realignment of RFs using ComBat can also reduce the effect of different imaging acquisition parameters [13, 14]. ComBat requires sufficient data to estimate these ‘batch’ effects, and the minimum ComBat batch size (MBS) must be chosen to ensure accurate results [13, 14]. MBS choice not only affects ComBat performance, but also discards some of the data within heterogenous, real-world images.

Inconsistent statistical modelling, which in GBM has tended to focus on prognostic separation (‘discrimination’) [11, 12], may also play a role in the lack of reproducibility. Model calibration and stability are important but less well-evaluated [15]. Calibration compares predictions to observed survival and stability and examines the consistency of model performance [16]. To date, the effect of ISTs and ComBat MBS choice has not been thoroughly assessed on model calibration and stability in a multi-centre setting [11, 17]. The aim of this study was to assess the effect of ISTs and ComBat MBS choice on calibration, discrimination, relative model fit, explained variation, and stability of prognostic models in a heterogenous, multi-centre cohort of patients with GBM, rather than producing the most accurate prognostic model for OS prediction in GBM.

## Materials and methods

### Ethical approval

This was a retrospective study and therefore informed patient consent was not feasible. Ethical approval and institutional data access were approved via the local ethical review committee (REC ref: 19/YH/0300, IRAS project ID: 255585). A completed Checklist for Artificial Intelligence in Medical Imaging (CLAIM) [18] is provided in Supplementary Materials.

### Patient selection and characteristics

A description of the patient cohort, selection criteria, data collection, and image preparation has been previously published [19]. Inclusion criteria: adults (> 16-years-old) with histologically proven GBM according to the 2021 World Health Organisation classification of central nervous system tumours treated between 2014 and 2020; MRI performed prior to surgery; unifocal tumour; and all four of: T1-weighted (T1W), T2-weighted (T2W), fluid-attenuated inversion recovery (FLAIR) and gadolinium contrast-enhanced T1W (T1CE) MRI. Exclusions: absence of pre-operative MRI; significant degradation of imaging due to artefact; multifocal tumour; and isocitrate dehydrogenase (IDH) mutation. Clinical predictors have been defined previously [19] (Supplementary Materials).

### Image preparation and tumour segmentation

**Fig. 1:**
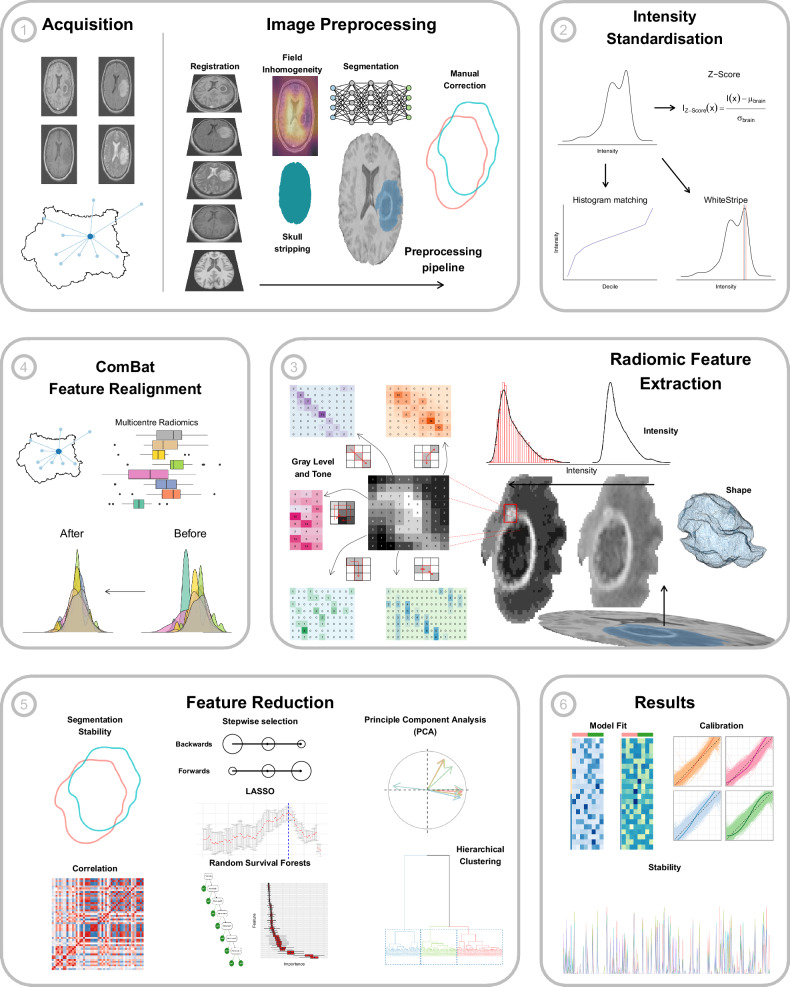
Methodological pipeline overview. Panels 1–6 outline the main steps of the experiment: (1) MRI scans acquired at multiple sites regionally pre-processed including registration, skull stripping, field inhomogeneity correction and tumour segmentation; (2) standardisation of MRI signal intensities; (3) RF extraction, including calculation of shape, intensity and higher-level features; (4) post-extraction realignment of multi-centre radiomics using ComBat; (5) application of feature reduction techniques to diminish data dimensionality; and (6) calculation of results and data analysis. FLAIR, fluid-attenuated inversion recovery; GLCM, grey-level co-occurrence matrix; GLDM, grey-level dependence matrix; GLRLM, grey-level run length matrix; GLSZM, grey-level size zone matrix; LASSO, least absolute shrinkage and selection operator; NGTDM, neighbouring grey tone difference matrix; T1, T1-weighted; T1CE, T1-weighted contrast-enhanced; T2, T2-weighted

A graphical illustration of the methodological pipeline is provided (Fig. 1). MRI studies were pre-processed and segmented using previously detailed methods [19] (Supplementary Materials). Key steps in the preparation and segmentation of imaging data are outlined, with further detail provided in the prior publication [19]. As a tertiary referral centre in the UK, it is standard practice for our institution to manage patients with GBM from the surrounding region (with a catchment of approximately four million people), which includes general hospitals (‘hub-and-spoke’ model).

The whole tumour and core volume (WV and CV, respectively) were segmented (Supplementary Materials) using a publicly available deep-learning model. CV was defined as enhancing and necrotic regions, and whole tumour volume (WTV) was defined as CV plus peritumoural high T2 signal (Fig. 1). Segmentations were checked manually and corrected by a board-certified neuroradiology fellow (5 years of radiology experience). Independently, 50 segmentations were also checked by a consultant neuroradiologist (> 10 years of consultant neuroradiology experience), and the inter-rater concordance was assessed using the dice similarity coefficient (DSC) [20].

### IS

**Fig. 2:**
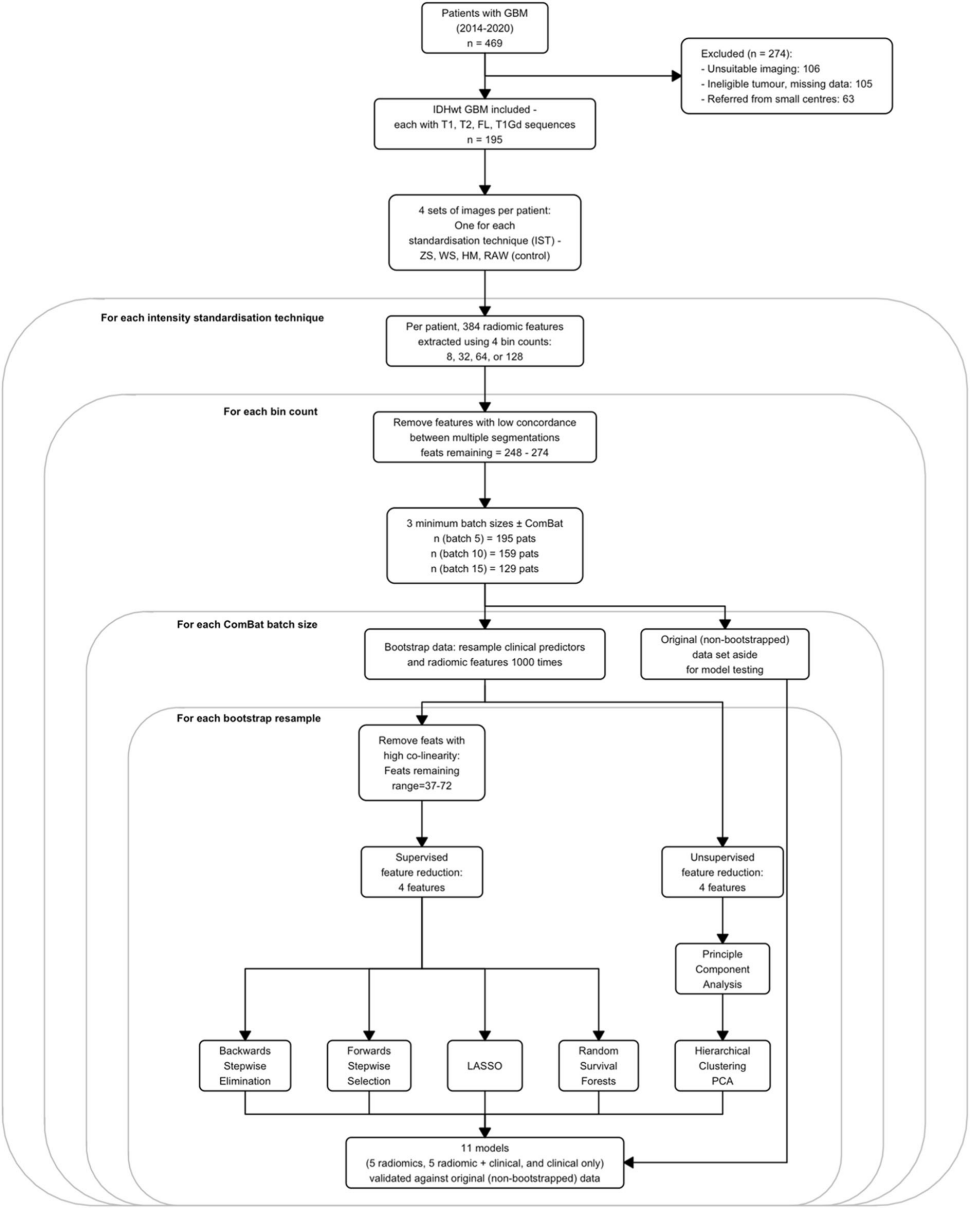
Flowchart of the statistical analysis. Note that because this study uses multi-centre data, but for some centres, the number of available patients is lower than the MBS, it results in a different number of patients in the samples (data on the number of patients per study site is contained in Supplementary Table 1a–d). FLAIR, fluid-attenuated inversion recovery; GBM, glioblastoma; HM, histogram matching; IDH, isocitrate dehydrogenase; LASSO, least absolute shrinkage and selection operator; PCA, principal component analysis; RAW, no IS applied to images (control); T1, T1-weighted; T1CE, T1-weighted contrast-enhanced; T2, T2-weighted; WS, WhiteStripe; ZS, *Z*-score

Three ISTs that are commonly used in patients with GBM [12] are WhiteStripe (WS) [21], Nyul histogram matching (HM) [22, 23] and *Z*-score (ZS). ZS and WS standardise intensities by subtracting the mean and dividing by the standard deviation of the whole brain or normal-appearing white matter intensity, respectively. HM produces a standardised intensity histogram by averaging the signal in a few scans, and this histogram is used to map the voxel intensities in images linearly onto the new scale (Supplementary Materials). Each IST was applied independently of the other, resulting in four separate images per sequence per patient (Fig. 2)—one per IST, plus the non-standardised images that served as control (‘RAW’ images).

### Radiomics feature extraction and ComBat feature realignment

PyRadiomics (v3.0.1) [24] was used to extract RFs from the WTV (Fig. 1). Three hundred eighty-four features were extracted from each image set (four sets, one per IST), including 18 first-order, 24 grey-level co-occurrence matrices, 16 grey-level run length matrices, 16 grey-level size zone matrices, 14 grey-level dependence matrices and 5 neighbouring grey-tone difference matrix features from each MR sequence, and 12 shape features extracted from the T1CE sequence. Features were extracted in 3 dimensions (3D), using a voxel size of 1 mm^3^. Four bin numbers (8, 32, 64, and 128) were used to extract four unique sets of RF per image to determine if ISTs were dependent on the bin number. Fixed bin numbers were used as they have a normalising effect [10]. After RF extraction, RFs with lower reproducibility between two independent WTV segmentations were removed if the intra-class correlation coefficient was below 0.8 (Supplementary Materials).

ComBat realignment was performed per MRI sequence, defining each batch not only on geographical location but also by the homogeneity of scan acquisition within sites (batch definition and acquisition parameters provided in Supplementary Table 1a–d). Age was entered as co-variate because this was found to vary significantly (*p* < 0.05) across batches (Supplementary Materials). Selecting the MBS represents a trade-off between increased performance of ComBat realignment against discarding too much data. A minimum of five patients has been previously identified as the lower limit for MBS [13, 25]. We chose three MBS values: 5, 10, or 15. Patients in smaller batches were excluded (Fig. 2) so 15 was the maximum to avoid excessive data loss. RFs without ComBat realignment were also included as a baseline assessment of IST alone.

### Statistical analysis and experimental settings

All statistical analysis was performed in R version 4.2.2 (2022-10-31) and overseen by a highly experienced statistician—a summary of the analysis is shown in Fig. 2. Cox proportional hazards (CPH) models for OS prediction (time from surgery to death, censor date 10/10/22) were built. 96 different combinations of ‘experimental settings’ (Fig. 2) were investigated; with and without ComBat, four ISTs, each with four bin counts and three MBS.

### Model building

Five feature selection (FS) methods were used to reduce dimensionality; four RFs were considered for entry into the radiomics model based on sample size calculations (Supplementary Materials). Each FS method was applied within each of the 1000 bootstraps resamples (Fig. 2) so that five sets of RFs were selected per bootstrap (Supplementary Table 2).

Unsupervised hierarchical clustering of patients was performed using principal component analysis (PCA) [26] of results. The four RFs explaining the most variation between clusters were retained. Prior to supervised FS, highly co-linear features were removed using a Spearman rank correlation range between 0.7 and − 0.7. Four supervised methods included CPH models with (1) backwards, (2) forward stepwise FS, and (3) with the LASSO. (4) Random survival forests (RSF) were trained, and the four most important RFs were selected with in-built functions (Supplementary Materials) [27].

In all, three models were produced. Each set of RFs was used to train a radiomics-only model. A clinical-only model was also trained as a baseline for results comparison using age, gender, O6-methylguanine-DNA methyltransferase (MGMT) promoter methylation, extent of surgical resection, oncological adjuvant treatment, tumour diameter and log-transformed WTV (prior analysis indicated log-transformation was the most effective non-linear transformation of WTV [19]). Clinical and radiomics features were then combined to produce a clinical-radiomics model.

### Model performance

Evaluating proposed prognostic models should include (at least) four domains: discrimination, calibration, relative model fit and relative explained variance (for more detail see Supplementary Materials). Calibration was assessed using the mean calibration slope and discrimination measured with Harrell’s *C*-index ($C$), and Royston and Sauerbrei’s D-statistic ($D$). Relative model fit was measured with Akaike’s information criterion (AIC) and relative explained variation with Royston and Sauerbrei’s ${R}^{2}$ (${R}_{D}^{2}$) and Nagelkerke’s ${R}^{2}$ (${R}_{N}^{2}$). Mean and 95% confidence intervals (95% CIs) were calculated across all 1000 bootstrap resamples (Fig. 2 and Supplementary Table 2). Bootstrapping, rather than a random train-test split, was used for optimism adjustment as it is recommended in statistical modelling literature [16].

Heatmaps were created to graphically illustrate the impact of ISTs and MBS. The heatmaps of discrimination, fit and explained variation were centred on the clinical-only model and scaled to the standard deviation of models for each experimental setting to highlight the change in model performance relative to the clinical-only model and allow comparison across settings [28]. For example, results for WS standardised images, bin count of 64 and MBS = 10 can be compared fairly to ZS images, bin count 32 and MBS = 15.

The impact of IST and MBS on model stability was assessed based on the size of 95% CIs for model performance measures the percentage of times that the same four features were selected together (feature co-occurrence), and the 1-year event prediction calibration plots across the 1000 bootstrap resamples.

## Results

### Study population

Cohort demographics are shown in Table 1 and are comparable to those in the scientific literature [29, 30]. Median survival was 13 months (95% CI: 12–14 months) following surgery, with 167 deaths (86%) occurring before the censor date.

**Table 1:** Summary of the main clinical, oncological and radiological features of the patient cohort (*n* = 195)

Demographic	Value
Age, years—median (IQR)	61 (55–68)
Gender—no. female (%)	72 (37%)
Surgical treatment—no. (%)	
Biopsy	44 (23%)
100% resected^a^	42 (22%)
≥ 90% resected^a^	62 (32%)
< 90% resected^a^	47 (24%)
Adjuvant oncology treatment—no. (%)	
No Stupp	102 (52%)
Full Stupp^b^	44 (23%)
Partial Stupp^c^	49 (25%)
MGMT methylation—no. (%)	70 (36%)
OS, months—median (95% CI)	13 (12–14)
Maximum tumour diameter, cm—median (IQR)	4.4 (3.35–5.35)
CV, cm^3^—median (IQR)	28.8 (13.4–50.9)
WTV, cm^3^—median (IQR)	107 (56.1–167)

*IQR* interquartile range, *MGMT* O6-methylguanine-DNA methyltransferase, *CI* confidence interval

^a^ Percentage of contrast-enhancing and necrotic tumour cores removed

^b^ Completed 60 Gy in 30 fractions radiotherapy with concomitant temozolomide and six cycles adjuvant temozolomide

^c^ Completed 60 Gy in 30 fractions radiotherapy with concomitant temozolomide and began adjuvant temozolomide

**Fig. 3:**
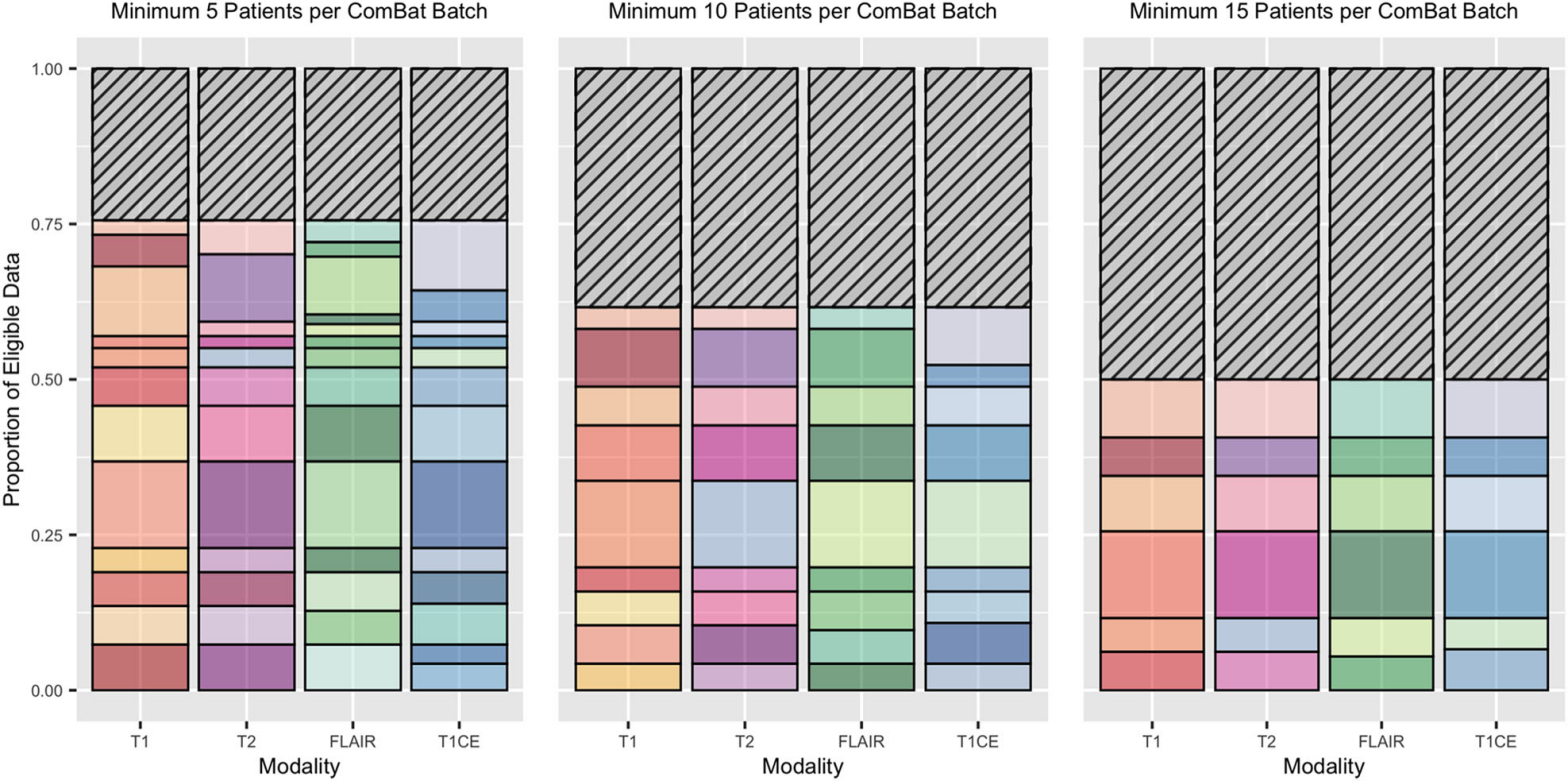
Bar charts demonstrating the proportion of eligible data used in the modelling process using three different ComBat MBSs. Three sets of stacked bar charts illustrate imaging data heterogeneity. Each bar represents one MRI sequence (*x*-axis), and the different colours/segments within each bar indicate a unique batch label for ComBat harmonisation. For example, this could indicate a different geographic location or a different set of acquisition parameters within the same location (see Supplementary Fig. 1a–c for a more in-depth key including each unique batch label, and Supplementary Table 1a–d for acquisition parameters per batch). The shaded regions indicate the proportion of imaging data that had to be excluded to meet the MBS. FLAIR, fluid-attenuated inversion recovery; T1, T1-weighted; T1CE, T1-weighted contrast-enhanced; T2, T2-weighted

Figure 3 and Supplementary Fig. 1a–c show the number of unique batches per MRI sequence in this heterogenous, multi-centre data. Depending on the sequence, there were 12–14 unique batches. 76% of eligible data was retained when MBS = 5 compared to 50% when MBS = 15.

### Model performance—effect of ISTs and ComBat batch size

**Fig. 4:**
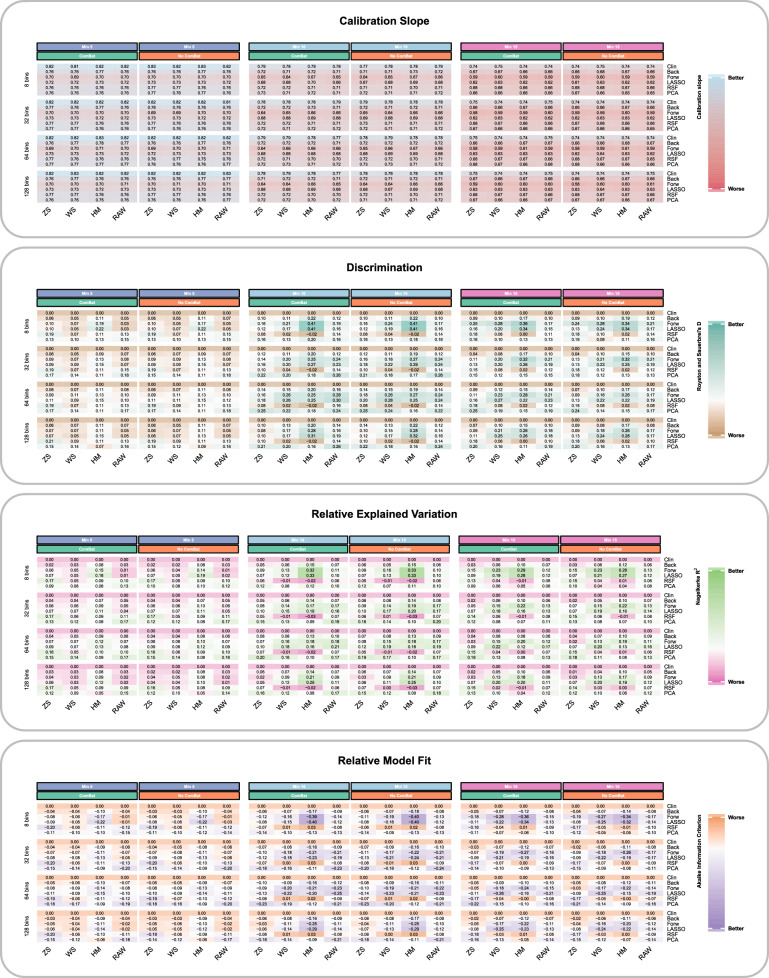
Heatmaps of model performance statistics per domain—calibration, discrimination, relative variance, and model fit. Heatmaps show the mean result per model performance statistic (averaged across the 1000 bootstrap resamples) for the clinical and the combined radiomic and clinical models across different selection procedures for all the experimental settings. The data for discrimination, relative explained variance and model fit statistics have been centred on the mean clinical value and scaled to the standard deviation across all models for that particular experimental setting (i.e. for each choice of minimum ComBat batch size, bin count and IS) so that it represents change relative to the clinical only model and allows more meaningful comparisons between different experiment settings. For each minimum ComBat batch size, there are two columns of results (indicated by the green/orange colour bars)—one indicating results with ComBat and one without ComBat realignment of RFs prior to modelling. CmB, ComBat; HM, histogram matching; LASSO, least absolute shrinkage and selection operator; PCA, hierarchical clustering of principle component results; RAW, no IS applied to images (control); RSF, random survival forests; WS, WhiteStripe; ZS, *Z*-score

A summary of the model performance for all experimental settings is shown in Fig. 4 and in Supplementary Table 4a–h).

### Model calibration and discrimination

Figure 4 shows that as MBS increased, the average calibration slope range decreased successively from 1 and there was little influence of ComBat, vs no ComBat. The heatmaps also demonstrated that the results for the average calibration slope for all ISTs compared to no standardisation (labelled ‘RAW’) were similar.

Both IST, MBS and the addition of ComBat affected discrimination. MBS 10 and 15 increased the range of scores compared to MBS 5, regardless of the use of ComBat. However, at MBS 15 the range of scores (0–0.36) with ComBat increased compared to without ComBat (0–0.34). The greatest relative improvement in discrimination was seen with LASSO or forward stepwise feature reduction, HM standardisation, eight bins and MBS ten patients regardless of the use of ComBat (0.41). Although not strictly observed, overall, HM and WS standardised images tended to produce the highest relative increase in discrimination compared to other ISTs.

### Relative explained variance and model fit

The additional benefit of ComBat was best seen with MBS 10 or 15 (at MBS 10, max scaled increase 0.29 with and 0.19 without and at MBS 15, 0.33 with and 0.28 without). For MBS 5 the addition of ComBat degraded the scores. The most increased scores were seen for HM (range − 0.03 to 0.26) or WS (− 0.01 to 0.22) standardised images and eight bins.

Model fit showed similar findings; the greatest improvements relative to the clinical model using ComBat compared to without was observed with MBS 15 (max scaled decrease − 0.36 and − 0.34, respectively). A lower score indicates improved relative model fit. At other MBSs, there was less benefit from ComBat realignment. RFs extracted with 8 bins, LASSO or forwards FS and HM standardisation produced the largest improvements in model fit (lowest AIC) and explained variation (highest R2). WS standardisation also performed well across most bin counts. As noted for discrimination, this was not a strictly observed finding and the result also depended upon which FS strategy was selected.

### Model stability

**Fig. 5:**
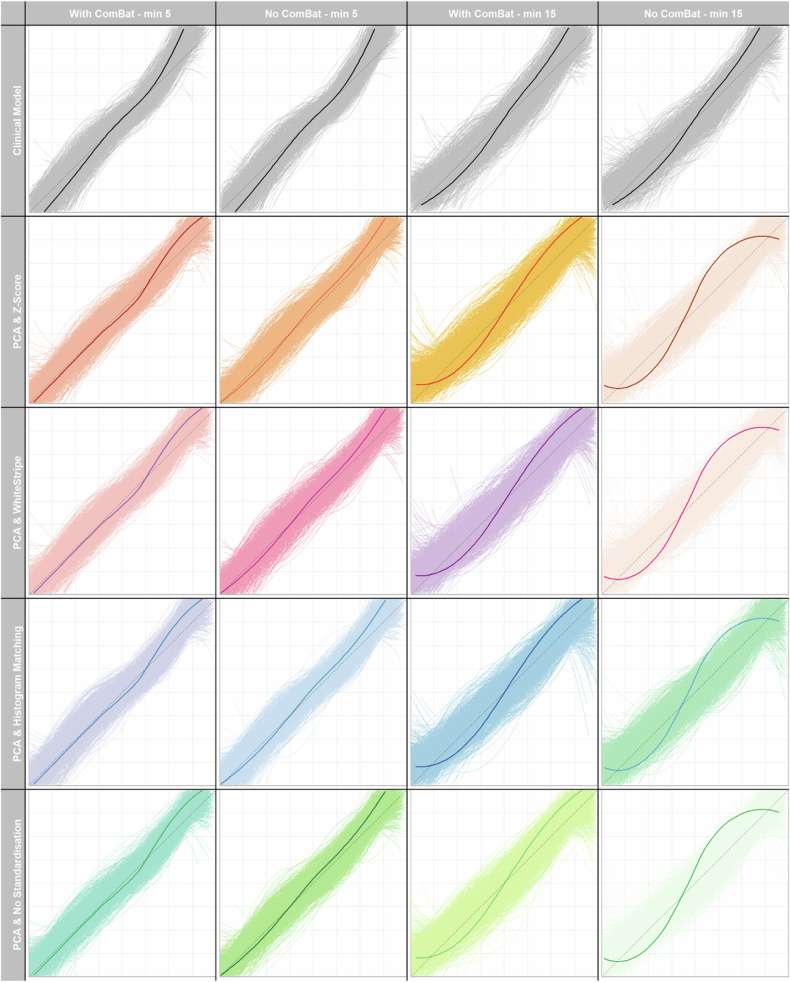
Calibration instability plots showing PCA FS clinical-radiomic combined models using 32 bins, different IS techniques, with and without ComBat realignment and showing the effects of different ComBat batch sizes (5 and 15). Calibration instability plots show, for the application of (columns 1 and 3), and without, ComBat (columns 2 and 4) using different MBSs (5 and 15) and IS techniques (rows), the results of individual survival predictions at 1 year, across the bootstrap resamples. *x*-Axes represent predicted and *y*-axes the observed survival at 1 year. The thin curves represent the predictions from one bootstrap sample and the thicker curve, predictions based on the original, non-bootstrapped data. Only 200, randomly selected, results are shown in each calibration plot. The grey dashed line represents a perfect calibration line, with greater deviation from this indicating worse calibration. Increased spread of the thinner curves indicates lower stability of that model building process. The calibration plots resulting from combined clinical and radiomics models, with features selected using hierarchical clustering of PCA results (rows 2, 3, and 4) are compared against the clinical-only models (grey, top row). CmB, ComBat; HM, histogram matching; PCA, hierarchical clustering of principle component results; RAW, no IS applied to images (control); WS, WhiteStripe; ZS, *Z*-score

The size of 95% CIs for model performance measures (Supplementary Table 4a–h), the frequency with which the same RFs were selected (Table 2) and the 1-year event prediction calibration plots (Fig. 5), all showed a trend towards reduced stability with increased MBS. All ISTs produced similar findings, as did models with and without ComBat realignment.

**Table 2:** Percentage of bootstrap resamples in which the same four RFs were selected for entry into the final model

Bin count	Feat select^b^	Percentage of resamples in which the same four radiomics features were selected											
		MBS for ComBat Realignment^a^											
		Minimum = 5				Minimum = 10				Minimum = 15			
		ZS	WS	HM	RAW	ZS	WS	HM	RAW	ZS	WS	HM	RAW
8	Backwards	< 1	< 1	< 1	< 1	< 1	< 1	< 1	< 1	< 1	< 1	< 1	< 1
	Forwards	**2**	1	1	< 1	1	1	1	< 1	< 1	1	1	< 1
	LASSO	1	1	1	< 1	< 1	**1**	< 1	< 1	< 1	< 1	< 1	< 1
	RSF	77	**78**	16	24	**75**	72	14	21	**72**	46	63	27
	PCA	7	7	6	6	5	5	**8**	5	6	7	6	7
32	Backwards	< 1	< 1	< 1	< 1	< 1	< 1	< 1	< 1	< 1	< 1	< 1	< 1
	Forwards	1	1	< 1	1	1	1	< 1	1	< 1	< 1	< 1	1
	LASSO	1	1	< 1	1	< 1	1	< 1	< 1	< 1	< 1	< 1	< 1
	RSF	27	**78**	16	20	25	**73**	16	19	**83**	47	67	25
	PCA	7	10	6	**12**	6	8	6	**10**	5	**8**	4	6
64	Backwards	< 1	< 1	< 1	< 1	< 1	< 1	< 1	< 1	< 1	< 1	< 1	< 1
	Forwards	1	1	1	1	< 1	1	< 1	1	< 1	< 1	1	< 1
	LASSO	1	1	1	1	1	1	< 1	1	< 1	1	< 1	< 1
	RSF	33	**80**	18	26	30	**77**	61	22	27	50	**61**	25
	PCA	10	14	8	14	8	**10**	7	8	8	**11**	5	7
128	Backwards	< 1	< 1	1	1	< 1	< 1	1	1	< 1	< 1	< 1	**1**
	Forwards	1	1	1	1	1	1	1	1	1	1	1	**2**
	LASSO	1	1	1	1	1	**2**	1	1	1	1	1	1
	RSF	**86**	79	66	22	**80**	74	61	18	**81**	46	63	22
	PCA	7	10	**16**	12	7	9	**10**	9	9	**12**	6	9

Results are shown for RFs with ComBat realignment, at all minimum ComBat batch sizes, bin counts and IS techniques and all five FS techniques. If one IS technique performed better than others, the result for that experimental setting is highlighted in bold

*HM* histogram matching, *LASSO* least absolute shrinkage and selection operator, *PCA* principal component analysis, *RAW* no IS prior to radiomic extraction, *WS* WhiteStripe standardisation, *ZS* *Z*-score IS

^a^ Minimum number of patients in the batch for ComBat realignment of RFs

^b^ Maximum of four RFs selected with the chosen method

The stability of calibration plots for 1-year event prediction using PCA FS and a bin count of 32 across all ISTs and ComBat batch sizes is illustrated in Fig. 5 (other FS calibration plots are shown in Supplementary Fig. 2a–d for bin count 32—other bin counts not included but illustrated similar findings). As the MBS increased, the stability of predictions decreased, as evidenced by greater spread from the null line of the bootstrapped results (shown in the paler colour). This was observed for all models and ISTs, with no IST clearly outperforming any other.

Similarly, the 95% CIs for model results (Supplementary Table 4a–h) showed a trend towards increased CI size, and hence lower stability, as the MBS was increased. Feature co-occurrence (Table 2) also showed the same trend, with fewer selection methods picking the same four RFs with increased MBS. As per the calibration plot results, the choice of IST did not show any trend with respect to model stability.

## Discussion

The aim of this project was to assess the effect of MRI IS technique and ComBat MBS on prognostic model performance including calibration and stability in a real-world, multi-centre GBM patient cohort. Results demonstrated worse calibration and model stability as MBS increased, and hence sample size decreased, however discrimination, explained variation, and model fit improved. HM and WS ISTs, overall, improved discrimination, and explained variation and model fit, which tended to occur at higher MBS, whereas choice of IST did not impact upon calibration or stability. The relative improvement of ComBat was mostly demonstrated at MBS 10 and 15, whereas there was little difference or even deterioration at lower MBS in some domains. By comparing across multiple domains of performance a more thorough assessment of ISTs and ComBat MBS was produced.

Previous studies that have compared the effect of ISTs on radiomics models [12] often show improved OS prediction [11, 17] or accuracy in differentiating grades of diffuse glioma [10, 31, 32]. Based on discrimination, relative fit or explained variation, performance improved through the choice of IST and, consistent with other studies [11, 12, 33], the current results show that HM and WS produced the highest relative improvements. However, for model calibration accuracy and model stability, IST did not affect results.

Adding ComBat slightly improved performance only at MBS 15 for discrimination and model fit, and at 10 and 15 for explained variation. This is explained by the likely increased accuracy of ComBat model coefficients estimation at higher MBS [14]. The application of ComBat to real-world datasets, however, poses a challenge due to the wide range of acquisitions and locations [13]. Previous studies have suggested that the MBS for ComBat could be as low as five [13, 25], however, others have suggested 20–30 minimum [14]. We opted for a compromise, which minimised data loss. A MBS 10 or 15 improved performance but this also made our models less stable, regardless of the addition of ComBat realignment. To our knowledge, no other studies have examined this impact. For real-world datasets, where scanner protocols are difficult to standardise across a broad geographical range and many centres, restricting the sample size for ComBat may not be a feasible option as it ignores the heterogeneity of imaging data, and more importantly, prediction models developed in this manner may not then be generalizable to sites with fewer patients. In our study, results without ComBat were similar to those with realignment, and a more practical solution may be to use fixed bin number discretisation and IST without ComBat in such data. Unsupervised clustering has been used to increase batch sizes [13, 34], grouping patients with similar RFs into clusters for ComBat realignment batches. However, the clustering results were not validated, and this approach would be difficult to validate with our sample size therefore we avoided this approach.

This study demonstrated a mixed picture regarding the effects of ISTs and ComBat batch sizes when we considered multiple domains of model performance and model stability. A systematic review of prognostic models in patients with GBM reported that 10 of 11 time-to-event models reported just the *C*-index [35]. A recent comparison of multiple ISTs in radiomics models in patients with ‘primary’ and recurrent high-grade glioma reported discrimination, using *C*-index, and relative model fit (AIC), but did not comment on calibration or ComBat MBS [11]. Our study also included a more in-depth assessment of model stability using bootstrapping, including calibration instability plots [16], which was a useful way to identify the consistency of model predictions. Stability is important as it provides information on how well a model performs following variations in input data, and not just how it performs on average [16].

The study has several limitations. Acquisition parameters were heterogeneous, including several centres with relatively few patients scanned, which impacted our ability to test larger batch sizes for ComBat. This is a real-world dataset, and the restriction of larger batches would have meant too few patients were included. The comparison of the relative impact of different ISTs could still be assessed, and this represents a case where good IS is required. Public data could have been used to supplement institutional data, however, the aim was to assess the performance of combined clinical-radiomic models, and hence well-curated data on clinical predictors were necessary. Future work could build on these results with additional public data. Only three out of many ISTs available were chosen for evaluation, however, these had previously been identified as the most popular choices in prior studies [19]. The supervised FS strategies considered far more than the four RFs suggested as the maximum by event per predictor calculation, however, they are popular within the literature and the decision will not have impacted upon our assessment of relative model performance due to IST and ComBat batch size. Finally, the measurement of IST impact on feature repeatability was not assessed, however, to the best of our knowledge, a preoperative GBM dataset with test–retest data is not available publicly.

## Conclusions

ISTs and ComBat MBS affected survival model performance in a heterogenous multi-centre GBM cohort. HM and WS, overall, improved discrimination, relative explained variation, and model fit, as did ComBat at higher MBS. However, calibration and model stability deteriorated as MBS increased, resulting in more data being discarded from modelling. This has clinical implications as referral systems such as the hub-and-spoke model in this study are hampered by varied image acquisitions, and therefore require robust methods for harmonising heterogenous datasets without compromising the model performance. Future work to demonstrate methods of improving radiomic model performance in real-world datasets that also preserve model stability is warranted.

## Supplementary information

ELECTRONIC SUPPLEMENTARY MATERIAL

## Abbreviations

CV: Core volume
CPH: Cox proportional hazards
FS: Feature selection
FLAIR: Fluid-attenuated inversion recovery
GBM: Glioblastoma
HM: Histogram matching
IS: Intensity standardisation
IST: Intensity standardisation technique
IDH: Isocitrate dehydrogenase
LASSO: Least absolute shrinkage and selection operator
MBS: Minimum ComBat batch size
MGMT: O6-methylguanine-DNA methyltransferase
OS: Overall survival
PCA: Principle component analysis
RF: Radiomic feature
RSF: Random survival forests
T1W: T1-weighted
T1CE: Gadolinium contrast-enhanced T1W
WS: WhiteStripe
WTV: Whole tumour volume
ZS: *Z*-score
