# Supplementary material for: Impact of intensity standardisation and ComBat batch size on clinical-radiomic prognostic models performance in a multi-centre study of patients with glioblastoma
Source: Eur Radiol. 2024 Nov 28;35(6):3354–66. doi: 10.1007/s00330-024-11168-7 (PMC12081554; doi:10.1007/s00330-024-11168-7)

Impact of intensity standardisation and ComBat batch size on clinical-radiomic prognostic models performance in a multi-centre study of patients with Glioblastoma.

ELECTRONIC SUPPLEMENTARY MATERIAL

Supplementary methods

Clinical variables

Clinical predictors included patient age, sex, and type of operation. Histopathological and cytogenetic data included histology, isocitrate dehydrogenase (IDH) 1 and 2 mutation and O6-methylguanine-DNA methyltransferase (MGMT) promoter methylation. Maximum axial or cranio-caudal diameter of the enhancing tumour core was measured using the gadolinium contrast-enhanced T1-weighted (T1CE) sequence. Extent of resection was estimated using the immediate (48-72 hour) post-resection MRI and grouped based upon the amount of contrast enhancing and necrotic tumour resected – (i) 100%, (ii) ≥90% or (iii) <90%. Adjuvant treatment was categorized as (i) full Stupp protocol – 60 Gy in 30 fractions radiotherapy with concomitant and 6 cycles adjuvant temozolomide; (ii) partial Stupp – 60 Gy in 30 fractions radiotherapy but temozolomide discontinued during either concomitant or adjuvant treatment phase; (iii) non-Stup – any other treatment protocol.

Image preparation and tumour segmentation

Digital Imaging and Communications in Medicine (DICOM) images were acquired across multiple centres across the region and historically transferred to a central picture archive and communication system (PACS) to facilitate routine patient care (acquisition parameters are summarised in **Supplemetary Tables 1a-d**). DICOM image preparation was performed in Python (Version 3.9) [1]. DICOM data was retrieved from PACS, pseudonymised and converted to Neuroimaging Informatics Technology Initiative (NIfTI) file format.

NIfTI images were processed and segmented using the open-source platform Federated Tumor Segmentation (FeTS) software, designed for performing these tasks on MRIs from patients with GBM [2]. T2-weighted (T2W), fluid attenuated inversion recovery (FLAIR) T2W, and T1CE sequences were rigidly co-registered to the T1-weighted (T1W) sequence, then to the SRI24 brain atlas [3], and spatially resampled to 1 x 1 x 1mm voxel resolution [4]. Images were skull-stripped [5] and tumours segmentated using the 'nnU-net' deep-learning network with pretrained model weights [6]. The tumour masks produced by the model were merged so that the whole tumour volume (WTV) was segmented, defined as the high T2/FLAIR signal region, which necessarily encompassed necrotic, enhancing, and peritumour oedema regions. FeTS software was also used to manually correct segmentations.

MR field inhomogeneity correction was performed with the N4ITK algorithm within the simple ITK package (Version 2.1.1.2) [7] before applying one of three MRI intensity standardisation techniques (ISTs).

Intensity standardisation (IS)

Full details for each technique and its implementation can be found online: https://github.com/jcreinhold/intensity-normalization.

Z-Score (ZS)

For each standardissed voxel ($I_{Z-Score}(x)$), the initial intensity ($I(x)$) is standardised by subtracting the mean intensity of all brain voxels (μ_{brain}), and then dividing it by the standard deviation of all the brain voxels' intensity values (σ_{brain}).

$$I_{Z-Score}(x) = \frac{I(x) - \mu_{brain}}{\sigma_{brain}}$$

WhiteStripe (WS)

Similarly, WS standardises each voxel ($I_{WhiteStripe}(x)$) by subtracting the mean intensity of normal appearing white matter (NAWM, μ_{NAWM}) and then dividing by the standard deviation of the intensity of NAWM (σ_{NAWM})[8].

$$I_{WhiteStripe}(x) = \frac{I(x) - \mu_{NAWM}}{\sigma_{NAWM}}$$

Histogram matching (HM)

Nyul's piecewise linear HM process [9] requires a standard histogram scale to be produced by averaging the intensity values from a subset of scans, using pre-defined intensity histogram landmarks (step-one). The landmarks are defined as percentiles, ranging from 1-99% of the intensity range (the default values are 1, 10, 20, 30, 40, 50, 60, 70, 80, 90 & 99%), such that outlier values are ignored [10]. All scans are then standardised by dividing the histogram of the new image into deciles and per decile, all voxels that fall into that range of intensities are linearly mapped using the standard scale produced in step-one [9, 10].

Radiomics Feature Extraction and ComBat feature realignment

A fixed bin number (FBN) was used rather than fixed bin size for intensity discretisation prior to texture feature calculation as previous work in diffuse glioma has suggested that a FBN may reduce the need for IST as it has a normalizing effect as bins are directly mapped to the intensity range of interest [11] and the Image Biomarker Standardardisation Initiative (IBSI) suggests this approach for MRI [12].

Pyradiomics is mostly compliant with the Image Biomarkmarker Standardardisation Initiative (IBSI) feature definitions. The definition of bin boundaries when using fixed bin sizes is different (not applicable to our study), pyradiomics aligns its resampling grid to the origin voxel (rather than to the centre), gray values are not rounded in pyradiomics and kurtosis is calculated as +3 compared to IBSI [12, 13].

ComBat is a statistical realignment process that aims to estimate the batch effects imparted onto radiomic features by imaging acquisition and variability in patient

demographics or clinical variables between sites. To decide which biological co-variates to include in the ComBat model, all continuous predictors were tested with one-way ANOVA and categorical predictors were tested with Fisher's exact test for significant differences ($p<0.05$) across batches. Ideally, all clinical predictors would have been added to the ComBat model as biological co-variates but this increases the sample size requirements for estimation of the batch effects [14], so a more pragmatic approach was adopted and only those with $p<0.05$ on significance testing were included.

Calculation of the intraclass correlation coefficients (ICCs) for radiomic features

The 50 patients that had their whole tumour volume segmented independently by two observers were used to measure the reproducibility of radiomic features. The two-way random effects ICC for each radiomic feature was measured by constructing a linear two-way random effects model with patient and segmentor selected as random effects. Reliable measurement of ICC assumes that the features are normally distributed (ie. follow a gaussian curve), hence the model residuals were checked visually for normality. If the residuals for a particular feature's model did not follow a normal distribution, the feature was power transformed using the Box-Cox (or Yeo-Johnson if they contained negative values), to attempt to shift their model residual distribution to a gaussian one. Radiomic features were excluded if, after a power transformation, the two-way random effects model residuals remained non-gaussian. A list of all transformed features, the lambda used for power-transformation and whether the transformation resulted in acceptance of the feature can be found in **Supplementary Table 3**. The radiomic features which could not be used in the comparison due to skewed residuals even after power-transformation, along with all transformed features and lambda values for power transformation are listed in **Supplementary Table 3**.

Calculation of sample size and event per predictor

Given that this was an exploratory analysis, comparing ISTs and ComBat batch sizes on model performance, rather than an exercise in producing the best prognostic model, we used all available data rather than calculating sample size a priori. However, as the number of candidate radiomic predictors was high, resulting in a low Event per Predictor

Parameter (EPP) rate, a number of feature reduction strategies were adopted and it was useful to know the minimum EPP available for modelling and this was calculated using previously published methodology [15, 16]. Of the GBM prognostic models identified in a recent systematic review of GBM prognostic models [17], none had published the Cox-Snell R^2 if the model had been applied in new data (adjusted Cox-Snell R^2, $R^2_{CS\_adj}$), which is the ideal parameter required for sample size calculation in time-to-event models [15, 16]. Therefore this had to be estimated from the minimum C-index (C) of models identified in the systematic review - 0.66 [17, 18]. The steps to estimating $R^2_{CS\_adj}$ are outlined here and further detail is found in the paper from Riley et al. [15]. This resulted in a minimum EPP of 22, which meant that four radiomic features were retained in the final candidate models, with suggested minimum sample size of 175.

First, Royston's D can be estimated from C:

$$D = 5.50(C - 0.5) + 10.26(C - 0.5)^2$$

Having estimated D from the reported C, $R^2_{D\_app}$ can be derived:

$$R^2_{D\_app} = \frac{\frac{\pi}{8}D^2}{\frac{\pi^2}{6} + \frac{\pi}{8}D^2}$$

$R^2_{D\_app}$ is used as a proxy for $R^2_{Royston\_app}$ to derive $R^2_{O\prime Quigley\_app}$:

$$R^2_{O\prime Quigley\_app} = \frac{-\frac{\pi^2}{6}R^2_{Royston\_app}}{\left(1 - \frac{\pi^2}{6}\right)R^2_{Royston\_app} - 1}$$

From $R^2_{O\prime Quigley\_app}$, the total number of events (E) used to derive the model (995) [18], the likelihood ratio (LR) of the model can be estimated:

$$LR = -E\ln\left(1 - R^2_{O\prime Quigley\_app}\right)$$

The apparent Cox-Snell R^2 ($R^2_{CS\_app}$) can then be derived, where n is the sample size (1354) [18]:

$$R^2_{CS\_app} = 1 - \exp\left(\frac{-LR}{n}\right)$$

Next, the Van Houwelingen and Le Cessie shrinkage factor (S_{VH}) can be derived, where p is the number of candidate predictor parameters (ie. all predictors that were tested for inclusion):

$$S_{VH} = 1 + \frac{p}{n\ln\left(1 - R^2_{CS\_app}\right)}$$

Finally, the adjusted Cox-Snell R^2 ($R^2_{CS\_adj}$) can be derived:

$$R^2_{CS\_adj} = S_{VH} R^2_{CS\_app}$$

From the $R^2_{CS\_adj}$, the R-package 'pmsampsize' [19] was used to calculate the minimum sample size and event per predictor parameter (EPP), using an event rate of 0.5 events/year based on median survival of patients with GBM being 12 months [17, 20] and a timepoint of 1 year.

Feature selection

Unsupervised selection used principle component analysis (PCA) and hierarchical clustering using the package 'FactoMineR' and default settings [21].PCA is a linear data reduction technique that describes the variation in the data using linear combinations of non-correlated features (principle components). Hierarchical clustering classified the PCA results so that 3 to 10 clusters were formed. Four RFs that explain the greatest variation between clusters were selected [21].

Cox proportional hazards (CPH) models using backwards or forwards stepwise feature selection and a p-value threshold of 0.1 was used until four features were included using the 'stepAIC' function [22]. CPH model with a least absolute shrinkage and selection operator (LASSO) penalty [23] and the smallest value of lambda that would select only four features was used. The optimal value of lambda was selected following

10-fold cross validation applied within each bootstrap resample. Random survival forests (RSFs) were used to select features using the package ‘RandomForestSRC’ [24]. Tuning the optimum number of features to split at each node, the minimum size of the terminal node and to determine the importance of features to the models was determined using the in-built functions. The four most important RFs were selected using this approach [24].

Model performance

Clinicians evaluating any proposed prognostic model need to assess it across (at least) four domains: discrimination, calibration, relative model fit and relative explained variance (supplementary methods).

Calibration shows how closely predictions match observed events; if an individual is predicted to have low risk of death, is the observed death rate also low for similar patients? This was assessed with the calibration slope and calibration plots for 1 year survival prediction. The variability of calibration of these predictions is important; a model that produces wildly different predictions after small alterations to training data indicates that the model is highly variable. Discrimination informs how good the model is at dividing patients into high or low risk groups. Harrell’s C-index (C) is the proportion of all pairs of individuals that can be ordered, in which the person with higher predicted risk has a lower survival. Often measured with Harrell’s C-index (C), a C of 1 is perfect discrimination and 0.5 indicates no discrimination. It has been suggested that comparing models using C is not informative and therefore Royston and Sauerbrei’s D-statistic (D). D is the log-hazard ratio for two equal sized groups that are split using the average (median) prognostic risk score. It starts at 0, with no upper bound was also calculated – a higher value is better.

Relative model fit provides an insight into which model (from all models built from this data), contains more information relating to outcome whilst using the fewest variables to do so; a more parsimonious model is generally a better one. Fit was measured with Akaike’s information criterion (AIC) - lower values suggest better model fit [25]. Relative explained variation indicates which model best explains the variation in survival times

for GBM patients for competing models. Two measures of explained variation were calculated - Royston and Sauerbrei's R^2 (R_D^2) and Nagelkerke's R^2 (R_N^2) - higher values indicate better performance [26]. Mean and 95% confidence intervals (95% CIs) were calculated across all 1000 bootstrap resamples (**Figure 2**, **Supplementary Table 2**).

Heatmaps were created to graphically illustrate the impact of ISTs and MBS. The heatmaps of discrimination, fit and explained variation were centered on the clinical-only model and scaled to the standard deviation of models for each experimental setting to highlight the change in model performance relative to the clinical-only model and allow comparison across settings [26]. For example, results for WS standardised images, bin count of 64 and MBS=10 can be compared fairly to ZS images, bin count 32 and MBS=15.

The impact of IST and MBS on feature selection stability was also assessed by measuring the percentage of times across bootstrap resamples that the same two, three or four features were selected together (feature co-occurrence).

Calibration plots

To make the calibration plots, the bootstrap resampling process outlined in **Supplementary Table 2** was followed to produce 1000 predictions for patient survival probabilities at 1 year. Since the data is censored, observed survival times would be potentially misleading and therefore estimated survival times were produced so that a smoothed calibration plot could be drawn using the package 'pseudo' [27]. This resulted in potentially 1000 curves that could be plot on the same axes, but 200 were randomly selected to enhance visualization.

Supplementary results

Segmentation

The mean (± standard deviation) DSC for WTV segmentations was 0.96 ± 0.03, which is equivalent to values published in the BRATS segmentation dataset, in which multiple expert raters segment the same GBM images, and our segmentation concordance was therefore within the expected variation of inter-rater agreement [28].

Feature reduction

ICC values of radiomic features from the two independent segmentations in the patients with two independent tumour masks resulted in removal of between 110-136 features (range across all bin counts and ISTs). For supervised feature selection, a range of 32-72 features remained following the removal of those with high co-linearity based on absolute Spearman correlation coefficient.

References

1. Van Rossum G, Drake FL (2009) Python 3 Reference Manual. CreateSpace, Scotts Valley, CA

2. Pati S, Baid U, Edwards B, et al (2022) Federated Learning Enables Big Data for Rare Cancer Boundary Detection. https://doi.org/10.48550/ARXIV.2204.10836

3. Rohlfing T, Zahr NM, Sullivan EV, Pfefferbaum A (2009) The SRI24 multichannel atlas of normal adult human brain structure. Human Brain Mapping 31:798–819. https://doi.org/10.1002/hbm.20906

4. Yushkevich PA, Pluta J, Wang H, et al (2016) IC-P-174: Fast Automatic Segmentation of Hippocampal Subfields and Medial Temporal Lobe Subregions In 3 Tesla and 7 Tesla T2-Weighted MRI. Alzheimer’s & Dementia 12:126–127. https://doi.org/10.1016/j.jalz.2016.06.205

5. Thakur S, Doshi J, Pati S, et al (2020) Brain extraction on MRI scans in presence of diffuse glioma: Multi-institutional performance evaluation of deep learning methods and robust modality-agnostic training. NeuroImage 220:117081. https://doi.org/10.1016/j.neuroimage.2020.117081

6. Isensee F, Jaeger PF, Kohl SAA, et al (2021) nnU-Net: a self-configuring method for deep learning-based biomedical image segmentation. Nature Methods 18:203–211. https://doi.org/10.1038/s41592-020-01008-z

7. Lowekamp BC, Chen DT, Ibáñez L, Blezek D (2013) The design of simpleITK. Frontiers in Neuroinformatics 7:1–14. https://doi.org/10.3389/fninf.2013.00045

8. Shinohara RT, Shiee N, Reich DS, et al (2014) Statistical normalization techniques for magnetic resonance imaging. NeuroImage: Clinical 6:9–19. https://doi.org/10.1016/j.nicl.2014.08.008

9. Nyúl LG, Udupa JK, Zhang X (2000) New variants of a method of MRI scale standardization. IEEE Transactions on Medical Imaging 19:143–150. https://doi.org/10.1109/42.836373

10. Shah M, Xiao Y, Subbanna N, et al (2011) Evaluating intensity normalization on MRIs of human brain with multiple sclerosis. Medical Image Analysis 15:267–282. https://doi.org/10.1016/j.media.2010.12.003

11. Carré A, Klausner G, Edjlali M, et al (2020) Standardization of brain MR images across machines and protocols: bridging the gap for MRI-based radiomics. Scientific Reports 10:12340. https://doi.org/10.1038/s41598-020-69298-z

12. Zwanenburg A, Leger S, Vallières M, Löck S (2016) Image biomarker standardisation initiative. https://doi.org/10.1148/radiol.2020191145

13. Pyradiomics Pyradiomics Frequently Asked Questions

14. Orlhac F, Eertink JJ, Cottereau A-S, et al (2022) A Guide to ComBat Harmonization of Imaging Biomarkers in Multicenter Studies. Journal of Nuclear Medicine 63:172–179. https://doi.org/10.2967/jnumed.121.262464

15. Riley RD, Snell KIE, Ensor J, et al (2019) Minimum sample size for developing a multivariable prediction model: PART II - binary and time-to-event outcomes. Statistics in Medicine 38:1276–1296. https://doi.org/10.1002/sim.7992

16. Riley RD, Ensor J, Snell KIE, et al (2020) Calculating the sample size required for developing a clinical prediction model. BMJ 368:m441. https://doi.org/10.1136/bmj.m441

17. Tewarie IA, Senders JT, Kremer S, et al (2021) Survival prediction of glioblastoma patients—are we there yet? A systematic review of prognostic modeling for glioblastoma and its clinical potential. Neurosurgical Review 44:2047–2057. https://doi.org/10.1007/s10143-020-01430-z

18. Gittleman H, Lim D, Kattan MW, et al (2017) An independently validated nomogram for individualized estimation of survival among patients with newly diagnosed glioblastoma: NRG Oncology RTOG 0525 and 0825. Neuro-Oncology 19:669–677. https://doi.org/10.1093/neuonc/now208

19. Ensor J (2023) pmsampsize: Sample Size for Development of a Prediction Model

20. Fatania K, Frood R, Mistry H, et al (2024) Tumour Size and Overall Survival in a Cohort of Patients with Unifocal Glioblastoma: A Uni- and Multivariable Prognostic Modelling and Resampling Study. Cancers 16:1301. https://doi.org/10.3390/cancers16071301

21. Le S, Josse J, Husson F (2008) FactoMineR: An R Package for Multivariate Analysis. Journal of Statistical Software 25:1–18

22. Venables W, Ripley B (2002) Modern Applied Statistics with S, Fourth. Springer, New York

23. Friedman J, Hastie T, Tibshirani R (2010) Regularization Paths for Generalized Linear Models via Coordinate Descent. Journal of Statistical Software 33:1–22. https://doi.org/10.18637/jss.v033.i01

24. Patel M, Zhan J, Natarajan K, et al (2021) Machine learning-based radiomic evaluation of treatment response prediction in glioblastoma. Clinical radiology 76:628.e17–628.e27. https://doi.org/https://dx.doi.org/10.1016/j.crad.2021.03.019

25. Sauerbrei W (1999) The use of resampling methods to simplify regression models in medical statistics. Journal of the Royal Statistical Society Series C: Applied Statistics 48:313–329. https://doi.org/10.1111/1467-9876.00155

26. Austin PC, Pencinca MJ, Steyerberg EW (2017) Predictive accuracy of novel risk factors and markers: A simulation study of the sensitivity of different performance measures for the Cox proportional hazards regression model. Statistical Methods in Medical Research 26:1053–1077. https://doi.org/10.1177/0962280214567141

27. Klein JP, Gerster M, Andersen PK, et al (2008) SAS and R functions to compute pseudo-values for censored data regression. Computer Methods and Programs in Biomedicine 89:289–300. https://doi.org/10.1016/j.cmpb.2007.11.017

28. Menze BH, Jakab A, Bauer S, et al (2015) The Multimodal Brain Tumor Image Segmentation Benchmark (BRATS). IEEE Transactions on Medical Imaging 34:1993–2024. https://doi.org/10.1109/TMI.2014.2377694

Supplementary Table 1a – Summary of patient, tumour and acquisition characteristics for T1-weighted images

Modality	Cluster	Count	Patient and tumour characteristics[a]								Acquisition parameters[b]																	
			Gender	Age	Biopsy	Gross resection[c]	Stupp[d]	MGMT[e]	Diameter (cm)	Volume (cm³)[f]	Series	Location[g]	Manufacturer	Model	Machine ID[g]	Field (T)	Pixel size (mm)	Slice thickness (mm)	Slice spacing (mm)	Orientation	Rows	Columns	Bandwidth (Hz)	Echo time (ms)	Echo train length	Inversion time (ms)	Repetition time (ms)	Flip angle (º)
T1	Batch 1	28	57	65 (51-85)	20	29	61	43	4.3 (1.4-8)	120 (17-230)	3D Fast Spin Echo	Site 6	Philips	Intera	Random ID 710	1.5	0.96 (0.9-0.96)	0.96 (0-1)	1	AX	260 (260-290)	260 (260-290)	240	3.6 (3.5-3.7)	200	-	7.7 (7.6-8)	8
	Batch 2	39	64	59 (35-78)	30	13	41	41	4.6 (2-7.9)	100 (5.4-250)	2D Spin Echo	Site 8	Philips	Achieva	Random ID 209	1.5	0.72	5	6	AX	320	320	140 (110-160)	13 (12-15)	1	-	600 (570-730)	64
	Batch 3	12	50	60 (49-74)	40	25	50	25	4.4 (1.3-6.6)	110 (8.9-220)	3D Fast Spin Echo	Site 5	Philips	Ingenia	Random ID 294	1.5	0.89	0.92 (0.9-1.1)	0.92 (0.9-1.1)	AX	290	290	220	3.5 (3.4-3.6)	220	-	7.8 (7.5-7.9)	8
	Batch 4	16	44	66 (48-74)	10	19	38	38	4.4 (2.3-7.3)	98 (7.3-250)	2D Spin Echo	Site 2	Siemens	Aera	Random ID 361	1.5	0.6	5	5.5	AX	380	340 (300-360)	130	7.7	1	-	530 (400-700)	86 (52-90)
	Batch 5	21	52	61 (44-81)	10	24	57	52	4.4 (2.7-7.5)	120 (19-280)	PROPELLOR	Site 3	Siemens	Aera	Random ID 940	1.5	0.92 (0.9-1.1)	4.2 (4-5)	5.4 (5.2-6.5)	AX	260	260	360	46	15	1100 (710-1300)	2700 (1600-3200)	150 (140-150)
	Batch 6	7	71	63 (41-80)	40	0	29	14	3.8 (3.3-8.5)	61 (46-160)	3D Fast Spin Echo	Site 4	GE	Discovery MR450	Random ID 544	1.5	0.47	1.2	0.6	COR	510	510	240	12	24	-	600	90
	Batch 7	13	85	71 (47-77)	8	31	31	54	4.9 (1.2-7.4)	140 (17-230)	2D Spin Echo	Site 7	Siemens	Aera	Random ID 679	1.5	0.72 (0.72-0.75)	5	6	AX	320	270 (250-320)	150	8.9	1	-	500 (410-550)	90
	Batch 8	17	65	65 (45-75)	40	18	41	35	4.3 (0.5-5.9)	89 (6.5-240)	3D Fast Spin Echo	Site 4	Siemens	Avanto Fit	Random ID 383	1.5	1 (0.98-1.1)	1	-	COR	260 (260-320)	200 (180-260)	750	11	63 (49-65)	-	690 (600-700)	120
	Batch 9	6	83	63 (55-68)	50	0	50	33	5.2 (2.4-6.7)	95 (16-130)	2D Spin Echo	Site 2	Siemens	Avanto	Random ID 118	1.5	0.6	5	5.5	AX	380	350 (310-360)	130	8.1 (7.8-9.4)	1	-	580 (500-660)	80 (58-90)
	Batch 10	15	67	56 (45-69)	7	40	67	33	5.7 (1.8-7.8)	150 (13-250)	2D Spin Echo	Site 1	Siemens	Avanto	Random ID 933	1.5	0.6 (0.6-0.62)	5.1 (5-7)	5.6 (5.5-7.7)	AX	380	350 (290-350)	130	7.8	1	-	510 (450-620)	86 (64-90)
	Batch 11	31	55	57 (34-81)	20	26	55	39	4.8 (1.4-7.4)	120 (8.4-220)	2D Spin Echo	Site 1	Siemens	Avanto	Random ID 534	1.5	0.61 (0.6-0.9)	5	5.5	AX	380 (260-380)	340 (260-350)	130	7.9 (7.8-12)	1	-	550 (450-680)	85 (57-90)
	Batch 12	20	70	59 (31-77)	20	25	40	20	3.9 (1.7-6.1)	85 (9-240)	2D Spin Echo	Site 2	Siemens	Aera	Random ID 78	1.5	0.6 (0.6-0.65)	5	5.5	AX	380	350 (310-360)	130	7.7	1	-	520 (400-660)	89 (82-90)

[a]Values for age, diameter and volume represent medians (range), and values for biopsy, gross resection, stupp and MGMT represent percentages of patients per batch

Modality	Cluster	Count	Patient and tumour characteristics[a]								Acquisition parameters[b]																	
			Gender	Age	Biopsy	Gross resection[c]	Stupp[d]	MGMT[e]	Diameter (cm)	Volume (cm³)[f]	Series	Location[g]	Manufacturer	Model	Machine ID[g]	Field (T)	Pixel size (mm)	Slice thickness (mm)	Slice spacing (mm)	Orientation	Rows	Columns	Bandwidth (Hz)	Echo time (ms)	Echo train length	Inversion time (ms)	Repetition time (ms)	Flip angle (º)

[b]Values represent mean (range) parameters per batch - ranges not stated for parameters that did not vary within the batch

[c]100% of enhancing and necrotic tumour removed according to radiological assessment

[d]Completed 60Gy in 30 fractions radiotherapy with concomitant temozolomide and began adjuvant temozolomide

[e]Percentage of patients per batch with methylation of the MGMT promoter

[f]Whole tumour volume (includes enhancement, necrosis and peritumoural high T2 signal)

[g]Site identifiers such as scanner location and machine identifier were anonymised

AX = axial; COR = coronal; GE = General Electric; MGMT = O6-methylguanine-DNA methyltransferase; PROPELLER = Periodically Rotated Overlapping ParallEL Lines with Enhanced Reconstruction; - = Not applicable or missing from DICOM header

Supplementary Table 1b – Summary of patient, tumour and acquisition characteristics for T2-weighted images

Modality	Cluster	Count	Patient and tumour characteristics[a]								Acquisition parameters[b]																	
			Gender	Age	Biopsy	Gross resection[c]	Stupp[d]	MGMT[e]	Diameter (cm)	Volume (cm³)[f]	Series	Location[g]	Manufacturer	Model	Machine ID[g]	Field (T)	Pixel size (mm)	Slice thickness (mm)	Slice spacing (mm)	Orientation	Rows	Columns	Bandwidth (Hz)	Echo time (ms)	Echo train length	Inversion time (ms)	Repetition time (ms)	Flip angle (º)
T2	Batch 1	17	59	57 (45-76)	6	35	71	24	5 (1.8-7.8)	110 (13-250)	2D Fast Spin Echo	Site 1	Siemens	Avanto	Random ID 255	1.5	0.51	5	5.5	AX	450	390 (340-390)	100	96	11	-	5400 (4900-5800)	150
	Batch 2	28	57	65 (51-85)	20	29	61	43	4.3 (1.4-8)	120 (17-230)	2D Fast Spin Echo	Site 6	Philips	Intera	Random ID 140	1.5	0.45 (0.41-0.57)	5	6	AX	530 (400-640)	530 (400-640)	200 (120-210)	110 (110-120)	20 (15-30)	-	5600 (4000-6300)	90
	Batch 3	37	65	59 (35-78)	30	14	43	41	4.6 (2-7.9)	100 (5.4-250)	2D Fast Spin Echo	Site 8	Philips	Achieva	Random ID 882	1.5	0.45	5	6	AX	510 (260-510)	510 (260-510)	140 (100-220)	100 (100-110)	15 (12-23)	-	5100 (4900-5500)	90
	Batch 4	10	50	60 (49-74)	40	20	50	20	4.4 (2.9-6.6)	110 (8.9-220)	2D Fast Spin Echo	Site 5	Philips	Ingenia	Random ID 430	1.5	0.41 (0.4-0.41)	5	6	AX	560 (560-580)	560 (560-580)	160 (150-180)	100	15	-	4900 (4500-5200)	90
	Batch 5	16	44	66 (48-74)	10	19	38	38	4.4 (2.3-7.3)	98 (7.3-250)	2D Fast Spin Echo	Site 2	Siemens	Aera	Random ID 969	1.5	0.51	5	5.5	AX	450	370 (340-390)	100	95	11	-	5600 (5400-6100)	150

Modality	Cluster	Count	Patient and tumour characteristics[a]								Acquisition parameters[b]																	
			Gender	Age	Biopsy	Gross resection[c]	Stupp[d]	MGMT[e]	Diameter (cm)	Volume (cm^3)[f]	Series	Location[g]	Manufacturer	Model	Machine ID[g]	Field (T)	Pixel size (mm)	Slice thickness (mm)	Slice spacing (mm)	Orientation	Rows	Columns	Bandwidth (Hz)	Echo time (ms)	Echo train length	Inversion time (ms)	Repetition time (ms)	Flip angle (º)
	Batch 6	21	52	61 (44-81)	10	24	57	52	4.4 (2.7-7.5)	120 (19-280)	2D Fast Spin Echo	Site 3	Siemens	Aera	Random ID 943	1.5	0.55 (0.51-0.6)	4	5.2	AX	450	330 (290-360)	170	80	19	-	4000 (3400-6600)	150
	Batch 7	28	57	58 (34-81)	20	29	50	43	4.6 (1.4-7.4)	130 (8.4-220)	2D Fast Spin Echo	Site 1	Siemens	Avanto	Random ID 233	1.5	0.51 (0.51-0.54)	5	5.5	AX	450	390 (340-450)	100 (100-130)	96 (90-110)	11 (11-15)	-	5400 (4100-6200)	150 (120-150)
	Batch 8	7	71	65 (55-68)	40	0	57	29	5.3 (2.4-6.7)	110 (16-160)	2D Fast Spin Echo	Site 2	Siemens	Avanto	Random ID 947	1.5	0.51	5	5.5	AX	450	380 (350-390)	100	96	11	-	5500 (5400-5800)	150
	Batch 9	5	100	67 (41-80)	40	0	20	20	3.8 (3.3-8.5)	61 (46-160)	2D Fast Spin Echo	Site 4	GE	Discovery MR450	Random ID 721	1.5	0.45 (0.43-0.47)	5	6	AX	510	510	200	98 (97-98)	24	-	5500 (5300-5700)	160
	Batch 10	8	88	72 (48-77)	0	25	25	25	4.7 (1.2-5.8)	110 (17-230)	2D Fast Spin Echo	Site 7	Siemens	Aera	Random ID 846	1.5	0.54 (0.39-0.56)	3	3.3	SAG	450	450	190	92 (83-110)	17	-	4300 (3800-4700)	150
	Batch 11	17	65	65 (45-75)	40	18	41	35	4.3 (0.5-5.9)	89 (6.5-240)	2D Fast Spin Echo	Site 4	Siemens	Avanto Fit	Random ID 669	1.5	0.57 (0.29-0.65)	5	6.5	AX	430 (380-770)	370 (290-670)	130	120	13	-	5000 (4600-5600)	150
	Batch 12	22	68	59 (31-77)	20	23	45	23	4 (1.7-6.1)	85 (9-270)	2D Fast Spin Echo	Site 2	Siemens	Aera	Random ID 901	1.5	0.51	5	5.5	AX	450	380 (350-410)	100 (100-190)	95 (95-96)	11 (11-17)	-	5600 (5300-6100)	150

[a]Values for age, diameter and volume represent medians (range), and values for biopsy, gross resection, stupp and MGMT represent percentages of patients per batch

[b]Values represent mean (range) parameters per batch - ranges not stated for parameters that did not vary within the batch

[c]100% of enhancing and necrotic tumour removed according to radiological assessment

[d]Completed 60Gy in 30 fractions radiotherapy with concomitant temozolomide and began adjuvant temozolomide

[e]Percentage of patients per batch with methylation of the MGMT promoter

[f]Whole tumour volume (includes enhancement, necrosis and peritumoural high T2 signal)

[g]Site identifiers such as scanner location and machine identifier were anonymised

AX = axial; GE = General Electric; MGMT = O6-methylguanine-DNA methyltransferase; SAG = Sagittal; - = Not applicable or missing from DICOM header

Supplementary Table 1c – Summary of patient, tumour and acquisition characteristics for Fluid Attenuated Inversion Recovery (FLAIR) images.

Modality	Cluster	Count	Patient and tumour characteristics[a]								Acquisition parameters[b]																	
			Gender	Age	Biopsy	Gross resection[c]	Stupp[d]	MGMT[e]	Diameter (cm)	Volume (cm³)[f]	Series	Location[g]	Manufacturer	Model	Machine ID[g]	Field (T)	Pixel size (mm)	Slice thickness (mm)	Slice spacing (mm)	Orientation	Rows	Columns	Bandwidth (Hz)	Echo time (ms)	Echo train length	Inversion time (ms)	Repetition time (ms)	Flip angle (º)
	Batch 1	10	80	56 (46-69)	10	30	60	10	5.9 (2.9-7.8)	180 (29-250)	2D Fast Spin Echo	Site 1	Siemens	Avanto	Random ID 732	1.5	0.45 (0.45-0.47)	5	5.5	AX	510	450	130	110	21	2500	9000	150
	Batch 2	24	62	64 (51-85)	20	25	58	42	4.3 (1.4-8)	130 (17-230)	2D Fast Spin Echo	Site 6	Philips	Intera	Random ID 941	1.5	0.46 (0.41-0.8)	5	6	AX	530 (290-640)	530 (290-640)	220 (200-320)	140	28 (27-50)	2800	10000 (10000-11000)	90
	Batch 3	38	66	59 (35-78)	30	13	39	39	4.6 (2-7.9)	100 (5.4-250)	2D Fast Spin Echo	Site 8	Philips	Achieva	Random ID 480	1.5	0.9	3.5	4.5	COR	260	260	370 (350-370)	120	47	2800	11000	90
	Batch 4	12	50	60 (49-74)	40	25	50	25	4.4 (1.3-6.6)	110 (8.9-220)	2D Fast Spin Echo	Site 5	Philips	Ingenia	Random ID 362	1.5	0.65	5	6	AX	350	350	390 (370-420)	130	53	2800	11000	90
	Batch 5	18	78	59 (31-72)	20	22	44	17	3.8 (1.7-6.1)	91 (9-270)	2D Fast Spin Echo	Site 2	Siemens	Aera	Random ID 242	1.5	0.87 (0.45-0.9)	5	5.5	AX	270 (260-510)	230 (210-420)	130	110	21	2500	9000	150
	Batch 6	15	47	66 (48-74)	10	20	33	40	4.4 (2.3-7.3)	100 (7.3-250)	2D Fast Spin Echo	Site 2	Siemens	Aera	Random ID 210	1.5	0.9	5	5.5	AX	260	220 (190-260)	150 (130-360)	110 (98-110)	22 (21-35)	2500	9000	150
FLAIR	Batch 7	21	52	61 (44-81)	10	24	57	52	4.4 (2.7-7.5)	120 (19-280)	2D Fast Spin Echo	Site 3	Siemens	Aera	Random ID 685	1.5	0.77 (0.72-0.84)	4	5.2	AX	320	230 (200-260)	180	84	17	1900 (1700-2000)	5300 (4600-5700)	150
	Batch 8	5	40	57 (45-76)	20	60	60	40	2.8 (1.8-7)	25 (13-190)	3D Fast Spin Echo	Site 1	Siemens	Avanto	Random ID 732	1.5	1.1	1.1	-	AX	260	190	440	470	110	1800	5000	120
	Batch 9	7	71	63 (41-80)	40	0	29	14	3.8 (3.3-8.5)	61 (46-160)	2D Spin Echo	Site 4	GE	Discovery MR450	Random ID 652	1.5	0.44 (0.43-0.47)	5	6	AX	510	510	120	120 (120-130)	1	2000	8000	160
	Batch 10	13	85	71 (47-77)	8	31	31	54	4.9 (1.2-7.4)	140 (17-230)	2D Fast Spin Echo	Site 7	Siemens	Aera	Random ID 217	1.5	0.72 (0.72-0.75)	5	6	AX	320	250 (240-260)	190	82	19 (16-22)	2300 (1800-2400)	7600 (5000-8000)	150
	Batch 11	16	62	66 (45-75)	40	19	38	38	4.4 (0.5-5.9)	100 (6.5-240)	2D Fast Spin Echo	Site 4	Siemens	Avanto Fit	Random ID 503	1.5	0.45 (0.43-0.49)	5	6.5	AX	510	420 (380-450)	130	110	21	2500	8800 (8000-9000)	150
	Batch 12	7	71	65 (55-68)	40	0	57	29	5.3 (2.4-6.7)	110 (16-160)	2D Fast Spin Echo	Site 2	Siemens	Avanto	Random ID 889	1.5	0.45	5	5.5	AX	510	430 (390-450)	130	110	21	2500	8800 (7700-9000)	150
	Batch 13	25	60	59 (37-81)	20	24	44	40	4.8 (1.8-7.4)	140 (8.4-220)	2D Fast Spin Echo	Site 1	Siemens	Avanto	Random ID 534	1.5	0.45 (0.45-0.47)	5	5.5	AX	510	440 (390-450)	130	110	21	2500	8900 (8000-9000)	150 (120-150)

Modality	Cluster	Count	Patient and tumour characteristics[a]								Acquisition parameters[b]																	
			Gender	Age	Biopsy	Gross resection[c]	Stupp[d]	MGMT[e]	Diameter (cm)	Volume (cm^3)[f]	Series	Location[g]	Manufacturer	Model	Machine ID[g]	Field (T)	Pixel size (mm)	Slice thickness (mm)	Slice spacing (mm)	Orientation	Rows	Columns	Bandwidth (Hz)	Echo time (ms)	Echo train length	Inversion time (ms)	Repetition time (ms)	Flip angle (º)
	Batch 14	5	20	54 (34-64)	20	40	100	40	3 (1.4-5.4)	21 (14-120)	3D Fast Spin Echo	Site 1	Siemens	Avanto	Random ID 534	1.5	1.1	1.1	-	AX	260	190	440	470	110	1800	5000	120

[a]Values for age, diameter and volume represent medians (range), and values for biopsy, gross resection, stupp and MGMT represent percentages of patients per batch

[b]Values represent mean (range) parameters per batch - ranges not stated for parameters that did not vary within the batch

[c]100% of enhancing and necrotic tumour removed according to radiological assessment

[d]Completed 60Gy in 30 fractions radiotherapy with concomitant temozolomide and began adjuvant temozolomide

[e]Percentage of patients per batch with methylation of the MGMT promoter

[f]Whole tumour volume (includes enhancement, necrosis and peritumoural high T2 signal)

[g]Site identifiers such as scanner location and machine identifier were anonymised

AX = axial; COR = coronal; FLAIR = Fluid Attenuated Inversion Recovery; GE = General Electric; MGMT = O6-methylguanine-DNA methyltransferase; - = Not applicable or missing from DICOM header

Supplementary Table 1d – Summary of patient, tumour and acquisition characteristics for post-contrast T1-weighted images.

Modality	Cluster	Count	Patient and tumour characteristics[a]								Acquisition parameters[b]																	
			Gender	Age	Biopsy	Gross resection[c]	Stupp[d]	MGMT[e]	Diameter (cm)	Volume (cm^3)[f]	Series	Location[g]	Manufacturer	Model	Machine ID[g]	Field (T)	Pixel size (mm)	Slice thickness (mm)	Slice spacing (mm)	Orientation	Rows	Columns	Bandwidth (Hz)	Echo time (ms)	Echo train length	Inversion time (ms)	Repetition time (ms)	Flip angle (º)
	Batch 1	27	59	65 (51-85)	20	30	63	44	4.3 (1.4-8)	110 (17-230)	3D Fast Spin Echo	Site 6	Philips	Intera	Random ID 590	1.5	0.96 (0.9-0.96)	0.98 (0.5-1)	0.98 (0.5-1)	AX	260 (260-290)	260 (260-290)	240	3.6 (3.5-3.7)	200	-	7.7 (7.6-8)	8
	Batch 2	39	64	59 (35-78)	30	13	41	41	4.6 (2-7.9)	100 (5.4-250)	2D Spin Echo	Site 8	Philips	Achieva	Random ID 321	1.5	0.72	5	6	AX	320	320	160 (110-160)	12 (12-15)	1	-	570 (540-730)	64
T1CE	Batch 3	11	45	61 (49-74)	50	18	45	27	4.4 (1.3-6.6)	110 (8.9-220)	3D Fast Spin Echo	Site 5	Philips	Ingenia	Random ID 358	1.5	0.89	0.92 (0.9-1.1)	0.92 (0.9-1.1)	AX	290	290	220	3.5 (3.4-3.6)	220	-	7.8 (7.5-7.9)	8
	Batch 4	16	69	62 (31-77)	20	19	38	19	3.8 (1.7-7.1)	85 (9-230)	3D MP-RAGE	Site 2	Siemens	Aera	Random ID 560	1.5	0.55	1.1	-	AX	510	380	250	2.3	1	1100	1900	15
	Batch 5	5	40	73 (45-76)	20	20	40	60	4.3 (1.5-6.2)	90 (32-190)	3D FLAIR	Unknown[h]	Siemens	Aera	Random ID 180	1.5	1 (0.98-1.1)	1.1 (1-1.2)	-	AX	250 (230-260)	240 (190-260)	170 (150-170)	4.4 (3.2-4.8)	1	900	410 (9.6-2000)	18 (8-20)

Modality	Cluster	Count	Patient and tumour characteristics[a]								Acquisition parameters[b]																	
			Gender	Age	Biopsy	Gross resection[c]	Stupp[d]	MGMT[e]	Diameter (cm)	Volume (cm^3)[f]	Series	Location[g]	Manufacturer	Model	Machine ID[g]	Field (T)	Pixel size (mm)	Slice thickness (mm)	Slice spacing (mm)	Orientation	Rows	Columns	Bandwidth (Hz)	Echo time (ms)	Echo train length	Inversion time (ms)	Repetition time (ms)	Flip angle (º)
	Batch 6	8	50	62 (50-74)	10	12	50	38	4.2 (3.5-5.3)	150 (63-180)	PROPELLER	Site 3	Siemens	Aera	Random ID 957	1.5	0.92 (0.9-0.98)	4.9 (4.5-5)	6.3 (5.8-6.5)	AX	260	260	360	46	15	840 (820-900)	2000 (1900-2100)	150
	Batch 7	31	58	58 (34-81)	20	26	52	42	4.5 (1.4-7.4)	120 (8.4-220)	3D MP-RAGE	Site 1	Siemens	Avanto	Random ID 488	1.5	0.55	1.1	-	AX	510 (480-510)	380 (340-380)	250	2.3	1	1100	1900 (1600-1900)	15
	Batch 8	7	71	63 (41-80)	40	0	29	14	3.8 (3.3-8.5)	61 (46-160)	3D Fast Spin Echo	Site 4	GE	Discovery MR450	Random ID 402	1.5	0.47	1.2	0.6	COR	510	510	240	12	24	-	570 (400-600)	90
	Batch 9	13	85	71 (47-77)	8	31	31	54	4.9 (1.2-7.4)	140 (17-230)	2D Spin Echo	Site 7	Siemens	Aera	Random ID 270	1.5	0.72 (0.72-0.75)	5	6	AX	320	280 (250-320)	150	9 (8.9-10)	1	-	500 (410-550)	90
	Batch 10	17	65	65 (45-75)	40	18	41	35	4.3 (0.5-5.9)	89 (6.5-240)	3D Fast Spin Echo	Site 4	Siemens	Avanto Fit	Random ID 24	1.5	1 (0.98-1.1)	1	-	COR	260 (250-260)	200 (180-260)	750	11	63 (49-65)	-	690 (600-700)	120
	Batch 11	15	67	57 (45-69)	10	40	67	27	5.7 (1.8-7.8)	150 (13-250)	3D MP-RAGE	Site 1	Siemens	Avanto	Random ID 989	1.5	0.55	1.1	-	AX	510 (450-510)	370 (280-380)	250	2.3	1	1100	1900 (1600-1900)	15
	Batch 12	6	83	63 (55-68)	50	0	50	33	5.2 (2.4-6.7)	95 (16-130)	3D MP-RAGE	Site 2	Siemens	Avanto	Random ID 975	1.5	0.55	1.1	-	AX	510	380	250	2.3	1	1100	1900	15
	Batch 13	18	56	62 (46-73)	10	17	39	33	4.4 (2.3-7.3)	110 (7.3-240)	3D MP-RAGE	Site 2	Siemens	Aera	Random ID 388	1.5	0.55	1.1	-	AX	510	380	250	2.3	1	1100	1900 (1600-1900)	15
	Batch 14	11	45	58 (44-71)	20	36	73	64	4.4 (2.7-7.5)	86 (19-280)	PROPELLER	Site 3	Siemens	Aera	Random ID 957	1.5	0.94 (0.9-1.1)	4	5.2	AX	260	260	360	46	15	1200 (910-1300)	2900 (2100-3200)	150 (140-150)

[a]Values for age, diameter and volume represent medians (range), and values for biopsy, gross resection, stupp and MGMT represent percentages of patients per batch

[b]Values represent mean (range) parameters per batch - ranges not stated for parameters that did not vary within the batch

[c]100% of enhancing and necrotic tumour removed according to radiological assessment

[d]Completed 60Gy in 30 fractions radiotherapy with concomitant temozolomide and began adjuvant temozolomide

[e]Percentage of patients per batch with methylation of the MGMT promoter

[f]Whole tumour volume (includes enhancement, necrosis and peritumoural high T2 signal)

[g]Site identifiers such as scanner location and machine identifier (ID) were anonymised

[h]Site name missing from DICOM header - unique batch identified using the machine identifier (ID)

AX = axial; COR = coronal; FLAIR = Fluid Attenuated Inversion Recovery; GE = General Electric; MGMT = O6-methylguanine-DNA methyltransferase; MP-RAGE = Magnetisation Prepared-RApid Gradient Echo PROPELLER = Periodically Rotated Overlapping ParallEL Lines with Enhanced Reconstruction; - = Not applicable or missing from DICOM header

Supplementary Table 2 – A step-by-step outline of the bootstrapping and model evaluation process

Steps involved in bootstrapping
1. Randomly sample patients ($\boldsymbol{b}$) from the original development dataset ($\boldsymbol{O}$), allowing duplicates to be selected, until the sample equals the size of original (sample size, $\boldsymbol{n}$).
2. Apply one of five feature selection strategies until the required number of radiomic features (four) is selected.
3. Create Cox proportional hazards models (I) using the selected radiomic features in sample $\boldsymbol{b}$ (radiomics only model, $\boldsymbol{Rad_b}$), (II) using only clinical variables in sample $\boldsymbol{b}$ (clinical only model, $\boldsymbol{Clin_b}$), and (III) a 'combined' model using the values of the selected radiomic features and clinical variables in sample $\boldsymbol{b}$ ($\boldsymbol{Comb_b}$).
4. Use the models produced in bootstrap sample $\boldsymbol{b}$ (step 3) to make survival predictions ($\widehat{\boldsymbol{p}}$) for each patient in the original data at a given time-point (1 year in our study). Hence, for each bootstrap sample, $\boldsymbol{b}$, $\boldsymbol{n}$ survival predictions ($\widehat{\boldsymbol{p}}$) will be produced ($\widehat{\boldsymbol{p}}_{\boldsymbol{b}}$, where $\boldsymbol{b}$ is each bootstrap sample).
5. Measure the 'test' performance of the models produced in the bootstrap sample $\boldsymbol{b}$, by supplying the original dataset $\boldsymbol{O}$ to the bootstrap models and calculate performance statistics $\boldsymbol{S_b}$.
6. Repeat steps 1-5 for a large number of repetitions ($\boldsymbol{B}$, $\boldsymbol{B = 1000}$ for our study).
7. Steps 1-6 will result in a set (of size between 1 and $\boldsymbol{B}$) of survival predictions and model performance statistics for each feature selection

process. This set is used to calculate the mean and 95% confidence interval of each model performance statistic.

8. Each of the predicted survival probabilities ($\hat{\boldsymbol{p}}_{\boldsymbol{b}}$) is plot against the survival that is actually observed in the original data $\boldsymbol{O}$ at 1-year (or another time point) to produce a calibration plot. Each bootstrap resample (between 1 and $\boldsymbol{B}$) will result in a unique calibration plot, and by overlaying these onto the same plotting region (up to $\boldsymbol{B}$ lines on one plot), a calibration instability plot can be drawn.

Supplementary Table 3 – List of radiomic features that were power-transformed using Box Cox transformation, the lambda value used and whether the feature was retained after transformation.

Bin Count	Intensity Standardisation	Lambda Used for Transformation	Feature Kept after Transformation	Imaging Modality	Feature Class	Feature Name
8	Z-Score	-0.5	Yes	FLAIR	gldm	SmallDependenceLowGrayLevelEmphasis
8	WhiteStripe	-0.5	Yes	FLAIR	gldm	SmallDependenceLowGrayLevelEmphasis
32	Z-Score	-0.7	Yes	FLAIR	gldm	LargeDependenceLowGrayLevelEmphasis
32	Z-Score	-0.8	Yes	FLAIR	gldm	LowGrayLevelEmphasis
64	Z-Score	-0.6	Yes	FLAIR	gldm	LargeDependenceLowGrayLevelEmphasis
64	Z-Score	-0.6	Yes	FLAIR	gldm	LowGrayLevelEmphasis
64	Z-Score	-0.7	Yes	FLAIR	glrlm	LongRunLowGrayLevelEmphasis
64	Z-Score	-0.4	Yes	T1	gldm	LargeDependenceLowGrayLevelEmphasis
128	Z-Score	-0.4	Yes	FLAIR	gldm	LargeDependenceLowGrayLevelEmphasis
128	Z-Score	-0.5	Yes	FLAIR	gldm	LowGrayLevelEmphasis
128	Z-Score	-0.5	Yes	FLAIR	glrlm	LongRunLowGrayLevelEmphasis
128	Z-Score	-0.1	Yes	FLAIR	glszm	LargeAreaLowGrayLevelEmphasis
128	Z-Score	-0.3	Yes	T1	gldm	LargeDependenceLowGrayLevelEmphasis
128	Z-Score	-0.3	Yes	T1	gldm	LowGrayLevelEmphasis
128	Z-Score	-0.4	Yes	T1	glrlm	LongRunLowGrayLevelEmphasis
128	Z-Score	-0.2	Yes	T1CE	gldm	LargeDependenceLowGrayLevelEmphasis
32	WhiteStripe	-0.7	Yes	FLAIR	gldm	LargeDependenceLowGrayLevelEmphasis
32	WhiteStripe	-0.8	Yes	FLAIR	gldm	LowGrayLevelEmphasis
32	WhiteStripe	-0.9	Yes	FLAIR	glrlm	LongRunLowGrayLevelEmphasis

64	WhiteStripe	-0.6	Yes	FLAIR	gldm	LargeDependenceLowGrayLevelEmphasis
64	WhiteStripe	-0.6	Yes	FLAIR	gldm	LowGrayLevelEmphasis
64	WhiteStripe	-0.7	Yes	FLAIR	glrlm	LongRunLowGrayLevelEmphasis
64	WhiteStripe	-0.4	Yes	T1	gldm	LargeDependenceLowGrayLevelEmphasis
128	WhiteStripe	-0.4	Yes	FLAIR	gldm	LargeDependenceLowGrayLevelEmphasis
128	WhiteStripe	-0.5	Yes	FLAIR	gldm	LowGrayLevelEmphasis
128	WhiteStripe	-0.5	Yes	FLAIR	glrlm	LongRunLowGrayLevelEmphasis
128	WhiteStripe	-0.1	Yes	FLAIR	glszm	LargeAreaLowGrayLevelEmphasis
128	WhiteStripe	-0.3	Yes	T1	gldm	LargeDependenceLowGrayLevelEmphasis
128	WhiteStripe	-0.3	Yes	T1	gldm	LowGrayLevelEmphasis
128	WhiteStripe	-0.4	Yes	T1	glrlm	LongRunLowGrayLevelEmphasis
128	WhiteStripe	-0.2	Yes	T1CE	gldm	LargeDependenceLowGrayLevelEmphasis
32	Histogram Matching	-0.6	Yes	FLAIR	gldm	LargeDependenceLowGrayLevelEmphasis
32	Histogram Matching	-0.7	Yes	FLAIR	gldm	LowGrayLevelEmphasis
32	Histogram Matching	-0.7	Yes	FLAIR	glrlm	LongRunLowGrayLevelEmphasis
64	Histogram Matching	-0.5	Yes	FLAIR	gldm	LargeDependenceLowGrayLevelEmphasis
64	Histogram Matching	-0.7	Yes	FLAIR	glrlm	LongRunLowGrayLevelEmphasis
64	Histogram Matching	-0.5	Yes	T1	gldm	LargeDependenceLowGrayLevelEmphasis
64	Histogram Matching	-0.4	Yes	T1	gldm	LowGrayLevelEmphasis
64	Histogram Matching	-0.6	Yes	T1	glrlm	LongRunLowGrayLevelEmphasis
64	Histogram Matching	-0.1	Yes	T1CE	gldm	LargeDependenceLowGrayLevelEmphasis
128	Histogram Matching	-0.4	Yes	FLAIR	gldm	LargeDependenceLowGrayLevelEmphasis
128	Histogram Matching	-0.4	Yes	FLAIR	gldm	LowGrayLevelEmphasis
128	Histogram Matching	-0.5	Yes	FLAIR	glrlm	LongRunLowGrayLevelEmphasis
128	Histogram Matching	0.0	Yes	FLAIR	glszm	LargeAreaLowGrayLevelEmphasis
128	Histogram Matching	-0.4	Yes	T1	gldm	LargeDependenceLowGrayLevelEmphasis
128	Histogram Matching	-0.4	Yes	T1	glrlm	LongRunLowGrayLevelEmphasis
128	Histogram Matching	-0.2	Yes	T1	glszm	LargeAreaLowGrayLevelEmphasis

128	Histogram Matching	-0.2	Yes	T1CE	gldm	LargeDependenceLowGrayLevelEmphasis
128	Histogram Matching	-0.3	Yes	T1CE	gldm	LowGrayLevelEmphasis
128	Histogram Matching	-0.4	Yes	T1CE	glrlm	LongRunLowGrayLevelEmphasis
32	No standardisation	-0.7	Yes	FLAIR	gldm	LargeDependenceLowGrayLevelEmphasis
32	No standardisation	-0.8	Yes	FLAIR	gldm	LowGrayLevelEmphasis
32	No standardisation	-0.9	Yes	FLAIR	glrlm	LongRunLowGrayLevelEmphasis
64	No standardisation	-0.6	Yes	FLAIR	gldm	LargeDependenceLowGrayLevelEmphasis
64	No standardisation	-0.6	Yes	FLAIR	gldm	LowGrayLevelEmphasis
64	No standardisation	-0.7	Yes	FLAIR	glrlm	LongRunLowGrayLevelEmphasis
64	No standardisation	-0.4	Yes	T1	gldm	LargeDependenceLowGrayLevelEmphasis
64	No standardisation	-0.5	Yes	T1	glrlm	LongRunLowGrayLevelEmphasis
128	No standardisation	-0.4	Yes	FLAIR	gldm	LargeDependenceLowGrayLevelEmphasis
128	No standardisation	-0.5	Yes	FLAIR	gldm	LowGrayLevelEmphasis
128	No standardisation	-0.5	Yes	FLAIR	glrlm	LongRunLowGrayLevelEmphasis
128	No standardisation	-0.1	Yes	FLAIR	glszm	LargeAreaLowGrayLevelEmphasis
128	No standardisation	-0.3	Yes	T1	gldm	LargeDependenceLowGrayLevelEmphasis
128	No standardisation	-0.4	Yes	T1	glrlm	LongRunLowGrayLevelEmphasis
128	No standardisation	-0.1	Yes	T1	glszm	LargeAreaLowGrayLevelEmphasis
128	No standardisation	-0.2	Yes	T1CE	gldm	LargeDependenceLowGrayLevelEmphasis

glcm = gray level co-occurrence matrix; gldm = gray level dependence matrix; glrlm = gray level run length matrix; glszm = gray level size zone matrix; FLAIR = Fluid Attenuated Inversion Recovery image; T1=T1-weighted image; T1CE = T1-weighted post-gadolinium image; T2 = T2-weighted image

Supplementary Table 4a – Model performance statistics for calibration, relative explained variation, relative model fit and discrimination by presenting mean and 95% confidence intervals of the statistics derived from 1000 bootstrap repetitions. Results are shown for 8 bin count with ComBat realignment. The models shown here are the clinical only and combined radiomics + clinical models, built using five different feature selection processes to select the radiomic features.

Batch size[a]	Feature Selection[b]	Model[c]	Calibration[d]				Relative Explained Variation[d]								Relative Model Fit[d]				Discrimination[d]							
			Calibration slope (Mean, 95% CI)[e]				Nagelkerke's R2 (Mean, 95% CI)[f]				Royston and Sauerbrei's R2[f]				Akaike Information Criterion (Mean, 95% CI)[g]				Royston and Sauerbrei's D (Mean, 95% CI)[hi]				Concordance Index			
			ZS	WS	HM	RAW	ZS	WS	HM	RAW	ZS	WS	HM	RAW	ZS	WS	HM	RAW	ZS	WS	HM	RAW	ZS	WS	HM	RAW
5 + C	Clinical Features	Clinical	0.82 (0.61 - 1.1)	0.82 (0.59 - 1.1)	0.81 (0.58 - 1.1)	0.83 (0.59 - 1.1)	0.27 (0.22 - 0.29)	0.27 (0.22 - 0.29)	0.27 (0.22 - 0.29)	0.27 (0.23 - 0.29)	0.21 (0.17 - 0.24)	0.21 (0.16 - 0.24)	0.21 (0.17 - 0.24)	0.21 (0.17 - 0.25)	1445 (1438 - 1456)	1445 (1438 - 1456)	1445 (1438 - 1456)	1444 (1438 - 1455)	1.1 (0.92 - 1.2)	1.1 (0.91 - 1.2)	1.1 (0.91 - 1.2)	1.1 (0.92 - 1.2)	0.72 (0.69 - 0.73)	0.71 (0.69 - 0.73)	0.72 (0.69 - 0.73)	0.72 (0.69 - 0.73)
	Backwards	Clin+Rad	0.76 (0.55 - 1)	0.76 (0.54 - 1)	0.76 (0.53 - 1)	0.77 (0.56 - 1)	0.27 (0.22 - 0.32)	0.27 (0.22 - 0.31)	0.27 (0.22 - 0.32)	0.28 (0.22 - 0.33)	0.23 (0.17 - 0.27)	0.22 (0.17 - 0.27)	0.22 (0.16 - 0.27)	0.23 (0.17 - 0.28)	1443 (1431 - 1457)	1443 (1432 - 1457)	1443 (1432 - 1458)	1441 (1428 - 1456)	1.1 (0.91 - 1.3)	1.1 (0.91 - 1.2)	1.1 (0.89 - 1.2)	1.1 (0.94 - 1.3)	0.72 (0.69 - 0.74)	0.71 (0.69 - 0.73)	0.72 (0.69 - 0.74)	0.72 (0.69 - 0.74)
	Forwards	Clin+Rad	0.7 (0.49 - 0.91)	0.7 (0.49 - 0.91)	0.7 (0.49 - 0.9)	0.7 (0.48 - 0.91)	0.27 (0.2 - 0.32)	0.28 (0.21 - 0.33)	0.28 (0.21 - 0.33)	0.29 (0.22 - 0.35)	0.22 (0.15 - 0.28)	0.23 (0.16 - 0.29)	0.23 (0.16 - 0.28)	0.25 (0.17 - 0.31)	1444 (1430 - 1463)	1442 (1426 - 1460)	1442 (1429 - 1460)	1438 (1421 - 1457)	1.1 (0.86 - 1.3)	1.1 (0.89 - 1.3)	1.1 (0.89 - 1.3)	1.2 (0.92 - 1.4)	0.71 (0.68 - 0.74)	0.71 (0.68 - 0.74)	0.71 (0.68 - 0.74)	0.72 (0.69 - 0.74)
	LASSO	Clin+Rad	0.72 (0.52 - 0.94)	0.72 (0.52 - 0.94)	0.72 (0.51 - 0.92)	0.73 (0.53 - 0.95)	0.27 (0.21 - 0.32)	0.28 (0.21 - 0.33)	0.28 (0.21 - 0.32)	0.3 (0.23 - 0.36)	0.22 (0.16 - 0.27)	0.23 (0.17 - 0.29)	0.23 (0.17 - 0.27)	0.25 (0.18 - 0.32)	1444 (1431 - 1460)	1441 (1428 - 1458)	1442 (1430 - 1459)	1436 (1419 - 1454)	1.1 (0.88 - 1.2)	1.1 (0.92 - 1.3)	1.1 (0.91 - 1.2)	1.2 (0.96 - 1.4)	0.71 (0.68 - 0.73)	0.72 (0.69 - 0.74)	0.71 (0.68 - 0.73)	0.72 (0.69 - 0.74)
	Random Survival Forests	Clin+Rad	0.76 (0.56 - 0.99)	0.76 (0.55 - 1)	0.76 (0.54 - 1)	0.76 (0.53 - 1)	0.29 (0.23 - 0.32)	0.29 (0.24 - 0.33)	0.27 (0.22 - 0.31)	0.28 (0.23 - 0.32)	0.24 (0.18 - 0.28)	0.25 (0.2 - 0.28)	0.23 (0.17 - 0.27)	0.23 (0.17 - 0.27)	1440 (1430 - 1455)	1437 (1428 - 1451)	1443 (1432 - 1456)	1440 (1429 - 1454)	1.2 (0.96 - 1.3)	1.2 (1 - 1.3)	1.1 (0.92 - 1.2)	1.1 (0.94 - 1.2)	0.72 (0.7 - 0.74)	0.72 (0.7 - 0.74)	0.72 (0.69 - 0.74)	0.72 (0.69 - 0.74)
	PCA + Clustering	Clin+Rad	0.76 (0.55 - 1)	0.76 (0.55 - 1)	0.75 (0.53 - 1)	0.76 (0.56 - 1)	0.29 (0.23 - 0.34)	0.28 (0.22 - 0.33)	0.28 (0.22 - 0.33)	0.28 (0.22 - 0.32)	0.24 (0.18 - 0.29)	0.24 (0.17 - 0.28)	0.23 (0.16 - 0.28)	0.24 (0.18 - 0.28)	1439 (1425 - 1455)	1440 (1428 - 1456)	1441 (1428 - 1458)	1440 (1430 - 1456)	1.2 (0.95 - 1.3)	1.1 (0.93 - 1.3)	1.1 (0.91 - 1.3)	1.1 (0.94 - 1.3)	0.72 (0.69 - 0.74)	0.72 (0.69 - 0.74)	0.72 (0.69 - 0.74)	0.72 (0.69 - 0.74)
10 + C	Clinical Features	Clinical	0.78 (0.53 - 1.1)	0.79 (0.54 - 1.1)	0.78 (0.53 - 1.1)	0.79 (0.53 - 1.1)	0.28 (0.23 - 0.31)	0.28 (0.23 - 0.31)	0.28 (0.23 - 0.31)	0.28 (0.22 - 0.31)	0.21 (0.16 - 0.25)	0.21 (0.15 - 0.25)	0.21 (0.15 - 0.25)	0.22 (0.15 - 0.25)	1139 (1132 - 1149)	1139 (1132 - 1150)	1138 (1132 - 1149)	1138 (1132 - 1150)	1.1 (0.88 - 1.2)	1.1 (0.85 - 1.2)	1.1 (0.87 - 1.2)	1.1 (0.87 - 1.2)	0.72 (0.69 - 0.74)	0.72 (0.69 - 0.74)	0.72 (0.69 - 0.74)	0.72 (0.69 - 0.74)
	Backwards	Clin+Rad	0.71 (0.47 - 0.97)	0.72 (0.47 - 1)	0.71 (0.47 - 0.97)	0.72 (0.48 - 0.98)	0.29 (0.22 - 0.35)	0.28 (0.21 - 0.34)	0.29 (0.22 - 0.34)	0.3 (0.22 - 0.36)	0.24 (0.15 - 0.31)	0.23 (0.15 - 0.3)	0.23 (0.15 - 0.3)	0.25 (0.16 - 0.32)	1136 (1122 - 1150)	1137 (1124 - 1152)	1136 (1124 - 1150)	1133 (1119 - 1150)	1.1 (0.87 - 1.4)	1.1 (0.85 - 1.3)	1.1 (0.86 - 1.3)	1.2 (0.91 - 1.4)	0.72 (0.69 - 0.75)	0.72 (0.69 - 0.74)	0.72 (0.69 - 0.74)	0.73 (0.69 - 0.75)

Batch size[a]	Feature Selection[b]	Model[c]	Calibration[d]				Relative Explained Variation[d]								Relative Model Fit[d]				Discrimination[d]							
			Calibration slope (Mean, 95% CI)[e]				Nagelkerke's R2 (Mean, 95% CI)[f]				Royston and Sauerbrei's R2[f]				Akaike Information Criterion (Mean, 95% CI)[g]				Royston and Sauerbrei's D (Mean, 95% CI)[hi]				Concordance Index			
			ZS	WS	HM	RAW	ZS	WS	HM	RAW	ZS	WS	HM	RAW	ZS	WS	HM	RAW	ZS	WS	HM	RAW	ZS	WS	HM	RAW
	Forwards	Clin+Rad	0.65 (0.44 - 0.88)	0.65 (0.42 - 0.89)	0.65 (0.43 - 0.87)	0.67 (0.45 - 0.9)	0.3 (0.21 - 0.36)	0.29 (0.2 - 0.36)	0.3 (0.21 - 0.37)	0.33 (0.24 - 0.4)	0.25 (0.16 - 0.33)	0.24 (0.15 - 0.33)	0.25 (0.15 - 0.34)	0.28 (0.18 - 0.36)	1134 (1119 - 1152)	1135 (1118 - 1155)	1133 (1117 - 1153)	1126 (1109 - 1146)	1.2 (0.89 - 1.4)	1.2 (0.86 - 1.4)	1.2 (0.85 - 1.5)	1.3 (0.96 - 1.5)	0.72 (0.69 - 0.75)	0.72 (0.69 - 0.75)	0.72 (0.69 - 0.75)	0.73 (0.7 - 0.76)
	LASSO	Clin+Rad	0.66 (0.45 - 0.9)	0.68 (0.46 - 0.91)	0.68 (0.47 - 0.91)	0.7 (0.48 - 0.94)	0.29 (0.22 - 0.35)	0.29 (0.21 - 0.36)	0.3 (0.22 - 0.36)	0.33 (0.23 - 0.4)	0.24 (0.15 - 0.32)	0.23 (0.15 - 0.32)	0.24 (0.15 - 0.33)	0.28 (0.18 - 0.36)	1135 (1121 - 1151)	1136 (1120 - 1153)	1134 (1119 - 1150)	1126 (1109 - 1149)	1.2 (0.87 - 1.4)	1.1 (0.88 - 1.4)	1.2 (0.87 - 1.4)	1.3 (0.97 - 1.5)	0.72 (0.69 - 0.75)	0.72 (0.69 - 0.74)	0.73 (0.69 - 0.75)	0.73 (0.7 - 0.76)
	Random Survival Forests	Clin+Rad	0.71 (0.48 - 0.98)	0.72 (0.49 - 1)	0.72 (0.49 - 0.98)	0.71 (0.47 - 1)	0.29 (0.22 - 0.33)	0.29 (0.22 - 0.33)	0.28 (0.21 - 0.32)	0.27 (0.2 - 0.31)	0.24 (0.17 - 0.3)	0.23 (0.16 - 0.27)	0.22 (0.15 - 0.27)	0.21 (0.15 - 0.26)	1136 (1126 - 1150)	1137 (1128 - 1150)	1139 (1129 - 1152)	1139 (1130 - 1155)	1.1 (0.93 - 1.3)	1.1 (0.91 - 1.2)	1.1 (0.87 - 1.2)	1.1 (0.84 - 1.2)	0.73 (0.7 - 0.75)	0.72 (0.69 - 0.74)	0.72 (0.69 - 0.74)	0.72 (0.68 - 0.74)
	PCA + Clustering	Clin+Rad	0.71 (0.47 - 0.98)	0.73 (0.48 - 1)	0.71 (0.46 - 0.98)	0.72 (0.47 - 0.98)	0.3 (0.23 - 0.36)	0.3 (0.23 - 0.35)	0.29 (0.22 - 0.36)	0.3 (0.22 - 0.35)	0.24 (0.16 - 0.32)	0.24 (0.16 - 0.31)	0.24 (0.16 - 0.32)	0.25 (0.16 - 0.31)	1134 (1120 - 1150)	1134 (1121 - 1149)	1135 (1120 - 1151)	1134 (1121 - 1150)	1.2 (0.88 - 1.4)	1.2 (0.9 - 1.4)	1.1 (0.88 - 1.4)	1.2 (0.91 - 1.4)	0.73 (0.69 - 0.75)	0.72 (0.69 - 0.75)	0.72 (0.69 - 0.75)	0.73 (0.7 - 0.75)
15 + C	Clinical Features	Clinical	0.74 (0.48 - 1)	0.74 (0.47 - 1.1)	0.75 (0.48 - 1.1)	0.74 (0.47 - 1.1)	0.27 (0.21 - 0.31)	0.27 (0.21 - 0.31)	0.27 (0.21 - 0.31)	0.27 (0.21 - 0.31)	0.21 (0.14 - 0.25)	0.21 (0.14 - 0.25)	0.21 (0.15 - 0.26)	0.21 (0.14 - 0.26)	884.1 (877.5 - 895.2)	884.2 (877.6 - 895.5)	884.1 (877.6 - 895.1)	884.2 (877.5 - 894.5)	1.1 (0.82 - 1.2)	1.1 (0.82 - 1.2)	1.1 (0.85 - 1.2)	1.1 (0.83 - 1.2)	0.72 (0.68 - 0.74)	0.72 (0.68 - 0.74)	0.72 (0.68 - 0.74)	0.72 (0.68 - 0.74)
	Backwards	Clin+Rad	0.66 (0.42 - 0.95)	0.67 (0.41 - 0.96)	0.67 (0.42 - 0.96)	0.66 (0.42 - 0.96)	0.28 (0.2 - 0.35)	0.28 (0.2 - 0.34)	0.28 (0.2 - 0.35)	0.29 (0.21 - 0.36)	0.23 (0.13 - 0.31)	0.22 (0.14 - 0.3)	0.23 (0.13 - 0.31)	0.24 (0.15 - 0.32)	882.1 (870.4 - 896)	883.1 (871.6 - 897)	882.5 (870.1 - 897.1)	881.3 (868.6 - 895.2)	1.1 (0.8 - 1.4)	1.1 (0.81 - 1.3)	1.1 (0.8 - 1.4)	1.1 (0.84 - 1.4)	0.72 (0.68 - 0.75)	0.72 (0.68 - 0.74)	0.72 (0.68 - 0.75)	0.72 (0.69 - 0.75)
	Forwards	Clin+Rad	0.59 (0.37 - 0.85)	0.59 (0.35 - 0.84)	0.6 (0.37 - 0.84)	0.59 (0.37 - 0.85)	0.29 (0.18 - 0.38)	0.3 (0.18 - 0.39)	0.31 (0.2 - 0.4)	0.32 (0.21 - 0.4)	0.24 (0.13 - 0.34)	0.24 (0.13 - 0.36)	0.25 (0.14 - 0.36)	0.27 (0.16 - 0.36)	880.4 (864.4 - 899.4)	879.9 (862.5 - 899.2)	877.4 (860 - 896.7)	875.6 (859.7 - 895.3)	1.2 (0.8 - 1.5)	1.2 (0.78 - 1.5)	1.2 (0.82 - 1.5)	1.2 (0.88 - 1.5)	0.72 (0.68 - 0.75)	0.72 (0.68 - 0.75)	0.73 (0.68 - 0.76)	0.73 (0.69 - 0.77)
	LASSO	Clin+Rad	0.62 (0.39 - 0.87)	0.62 (0.38 - 0.9)	0.63 (0.4 - 0.89)	0.62 (0.39 - 0.88)	0.29 (0.2 - 0.36)	0.29 (0.19 - 0.37)	0.3 (0.22 - 0.38)	0.32 (0.22 - 0.39)	0.24 (0.14 - 0.33)	0.23 (0.13 - 0.33)	0.24 (0.16 - 0.34)	0.26 (0.17 - 0.35)	880.7 (867.9 - 896)	881.7 (865.9 - 897.8)	878.7 (864 - 894)	876.1 (861 - 893.8)	1.1 (0.83 - 1.4)	1.1 (0.8 - 1.4)	1.2 (0.88 - 1.5)	1.2 (0.92 - 1.5)	0.72 (0.69 - 0.75)	0.72 (0.68 - 0.75)	0.73 (0.69 - 0.75)	0.73 (0.69 - 0.77)
	Random Survival Forests	Clin+Rad	0.66 (0.42 - 0.94)	0.68 (0.43 - 0.95)	0.68 (0.42 - 0.95)	0.66 (0.41 - 0.95)	0.29 (0.2 - 0.34)	0.29 (0.21 - 0.34)	0.28 (0.2 - 0.34)	0.27 (0.19 - 0.32)	0.23 (0.14 - 0.29)	0.23 (0.16 - 0.29)	0.22 (0.14 - 0.29)	0.21 (0.13 - 0.27)	881.7 (872.5 - 896.1)	880.5 (870.9 - 894.4)	883.1 (872.8 - 897.2)	884.5 (874.8 - 898)	1.1 (0.82 - 1.3)	1.1 (0.89 - 1.3)	1.1 (0.83 - 1.3)	1.1 (0.79 - 1.3)	0.73 (0.69 - 0.75)	0.72 (0.68 - 0.74)	0.72 (0.68 - 0.74)	0.71 (0.68 - 0.74)
	PCA + Clustering	Clin+Rad	0.66 (0.4 - 0.94)	0.66 (0.39 - 0.95)	0.66 (0.4 - 0.96)	0.66 (0.39 - 0.98)	0.29 (0.19 - 0.35)	0.29 (0.2 - 0.35)	0.28 (0.19 - 0.34)	0.28 (0.18 - 0.35)	0.23 (0.13 - 0.31)	0.23 (0.14 - 0.31)	0.23 (0.13 - 0.3)	0.23 (0.13 - 0.31)	881.5 (869.6 - 898.2)	881.7 (870.4 - 897.1)	882.5 (871.6 - 897.6)	882.7 (870.6 - 898.9)	1.1 (0.81 - 1.4)	1.1 (0.82 - 1.4)	1.1 (0.8 - 1.3)	1.1 (0.8 - 1.4)	0.73 (0.69 - 0.75)	0.72 (0.69 - 0.75)	0.72 (0.69 - 0.75)	0.72 (0.69 - 0.75)

CI = Confindence Interval; HM = Histogram Matching; LASSO = Least Absolute Shrinkage and Selection Operator; PCA = Principle Component Analysis; RAW = No intensity standardisation prior to radiomic extraction; WS = WhiteStripe standardisation; ZS = Z-Score intensity standardisation

[a]Minimum number of patients in each ComBat batch for realignment of radiomic features

Batch size[a]	Feature Selection[b]	Model[c]	Calibration[d]				Relative Explained Variation[d]								Relative Model Fit[d]				Discrimination[d]							
			Calibration slope (Mean, 95% CI)[e]				Nagelkerke's R2 (Mean, 95% CI)[f]				Royston and Sauerbrei's R2[f]				Akaike Information Criterion (Mean, 95% CI)[g]				Royston and Sauerbrei's D (Mean, 95% CI)[hi]				Concordance Index			
			ZS	WS	HM	RAW	ZS	WS	HM	RAW	ZS	WS	HM	RAW	ZS	WS	HM	RAW	ZS	WS	HM	RAW	ZS	WS	HM	RAW

[b]Maximum of four radiomic features selected with the chosen method

[c]Clinical features only or a combination of both clinical and radiomic features in the Cox proportional hazards model

[d]All performance measures indicate the value derived from the 'test' sample (ie. data withheld from bootstrap resample, and not used to build initial/training model)

[e]Overall calibration slope of model - Values closer to 1 indicate a better calibration

[f]Values range from 0 to 1, with higher values suggesting higher relative explanation of variation in outcome by the model compared to other model fit using same data

[g]Lower values suggest an improvement in relative model fit compared to other models fit using same dataset

[h]Values represent prognostic separation of two equal sized groups, split by the median risk score (log hazard ratio for linear predictor of Cox model) - values further from 0 suggest better discrimination

[i]Values range from 0.5 to 1, with values closer to 1 suggesting better model discrimination

Supplementary Table 4b – Model performance statistics for calibration, relative explained variation, relative model fit and discrimination by presenting mean and 95% confidence intervals of the statistics derived from 1000 bootstrap repetitions. Results are shown for 8 bin count, without ComBat realignment. The models shown here are the clinical only and combined radiomics + clinical models, built using five different feature selection processes to select the radiomic features.

Batch size[a]	Feature Selection[b]	Model[c]	Calibration[d]				Relative Explained Variation[d]								Relative Model Fit[d]				Discrimination[d]							
			Calibration slope (Mean, 95% CI)[e]				Nagelkerke's R2 (Mean, 95% CI)[f]				Royston and Sauerbrei's R2[f]				Akaike Information Criterion (Mean, 95% CI)[g]				Royston and Sauerbrei's D (Mean, 95% CI)[hi]				Concordance Index			
			ZS	WS	HM	RAW	ZS	WS	HM	RAW	ZS	WS	HM	RAW	ZS	WS	HM	RAW	ZS	WS	HM	RAW	ZS	WS	HM	RAW
5 - C	Clinical Features	Clinical	0.82 (0.59 - 1.1)	0.83 (0.6 - 1.1)	0.82 (0.61 - 1.1)	0.83 (0.6 - 1.1)	0.27 (0.22 - 0.29)	0.27 (0.23 - 0.29)	0.27 (0.23 - 0.29)	0.27 (0.23 - 0.29)	0.21 (0.16 - 0.25)	0.21 (0.17 - 0.25)	0.21 (0.17 - 0.25)	0.21 (0.17 - 0.24)	1445 (1438 - 1456)	1445 (1438 - 1455)	1444 (1438 - 1456)	1445 (1438 - 1455)	1.1 (0.91 - 1.2)	1.1 (0.92 - 1.2)	1.1 (0.92 - 1.2)	1.1 (0.92 - 1.2)	0.71 (0.69 - 0.73)	0.72 (0.69 - 0.73)	0.72 (0.69 - 0.73)	0.72 (0.69 - 0.73)
	Backwards	Clin+Rad	0.76 (0.53 - 1)	0.76 (0.55 - 1)	0.76 (0.55 - 1)	0.77 (0.56 - 1)	0.27 (0.22 - 0.32)	0.27 (0.22 - 0.31)	0.27 (0.22 - 0.31)	0.28 (0.23 - 0.33)	0.22 (0.16 - 0.27)	0.22 (0.16 - 0.27)	0.22 (0.16 - 0.27)	0.23 (0.17 - 0.28)	1443 (1431 - 1457)	1443 (1433 - 1458)	1443 (1432 - 1457)	1441 (1428 - 1456)	1.1 (0.91 - 1.3)	1.1 (0.9 - 1.2)	1.1 (0.89 - 1.2)	1.1 (0.94 - 1.3)	0.72 (0.69 - 0.74)	0.71 (0.69 - 0.73)	0.71 (0.69 - 0.73)	0.72 (0.69 - 0.74)
	Forwards	Clin+Rad	0.7 (0.49 - 0.93)	0.7 (0.46 - 0.92)	0.7 (0.5 - 0.92)	0.7 (0.49 - 0.9)	0.27 (0.2 - 0.32)	0.28 (0.2 - 0.33)	0.28 (0.21 - 0.32)	0.29 (0.22 - 0.35)	0.22 (0.15 - 0.27)	0.23 (0.15 - 0.29)	0.23 (0.16 - 0.27)	0.24 (0.17 - 0.31)	1444 (1430 - 1462)	1442 (1426 - 1462)	1442 (1430 - 1459)	1438 (1422 - 1457)	1.1 (0.86 - 1.3)	1.1 (0.85 - 1.3)	1.1 (0.89 - 1.3)	1.2 (0.92 - 1.4)	0.71 (0.68 - 0.74)	0.71 (0.68 - 0.74)	0.71 (0.68 - 0.74)	0.72 (0.69 - 0.75)
	LASSO	Clin+Rad	0.72 (0.51 - 0.95)	0.73 (0.52 - 0.96)	0.73 (0.52 - 0.96)	0.73 (0.55 - 0.97)	0.27 (0.2 - 0.31)	0.28 (0.21 - 0.33)	0.28 (0.22 - 0.32)	0.3 (0.23 - 0.35)	0.22 (0.15 - 0.27)	0.23 (0.17 - 0.29)	0.23 (0.16 - 0.27)	0.25 (0.19 - 0.31)	1444 (1432 - 1461)	1442 (1428 - 1458)	1442 (1430 - 1458)	1436 (1422 - 1453)	1.1 (0.85 - 1.2)	1.1 (0.93 - 1.3)	1.1 (0.89 - 1.3)	1.2 (1 - 1.4)	0.71 (0.69 - 0.73)	0.72 (0.69 - 0.74)	0.71 (0.69 - 0.74)	0.72 (0.69 - 0.74)
	Random Survival Forests	Clin+Rad	0.76 (0.54 - 1)	0.77 (0.56 - 1)	0.77 (0.55 - 1)	0.77 (0.54 - 1)	0.28 (0.23 - 0.32)	0.29 (0.24 - 0.33)	0.28 (0.22 - 0.32)	0.28 (0.23 - 0.32)	0.24 (0.18 - 0.28)	0.25 (0.19 - 0.28)	0.23 (0.16 - 0.27)	0.23 (0.18 - 0.27)	1440 (1430 - 1455)	1437 (1428 - 1451)	1442 (1431 - 1456)	1440 (1430 - 1454)	1.1 (0.95 - 1.3)	1.2 (1 - 1.3)	1.1 (0.91 - 1.2)	1.1 (0.96 - 1.3)	0.72 (0.69 - 0.74)	0.72 (0.7 - 0.74)	0.72 (0.69 - 0.74)	0.72 (0.69 - 0.74)
	PCA + Clustering	Clin+Rad	0.76 (0.54 - 1)	0.77 (0.54 - 1)	0.76 (0.54 - 1)	0.77 (0.54 - 1)	0.29 (0.23 - 0.33)	0.28 (0.22 - 0.33)	0.28 (0.22 - 0.33)	0.28 (0.23 - 0.32)	0.24 (0.17 - 0.29)	0.24 (0.17 - 0.28)	0.23 (0.17 - 0.28)	0.24 (0.18 - 0.28)	1439 (1426 - 1455)	1440 (1428 - 1457)	1441 (1428 - 1457)	1440 (1429 - 1455)	1.2 (0.93 - 1.3)	1.1 (0.92 - 1.3)	1.1 (0.91 - 1.3)	1.1 (0.95 - 1.3)	0.72 (0.69 - 0.74)	0.72 (0.69 - 0.74)	0.72 (0.69 - 0.74)	0.72 (0.69 - 0.74)
10 - C	Clinical Features	Clinical	0.79 (0.53 - 1.1)	0.77 (0.51 - 1.1)	0.78 (0.52 - 1.1)	0.78 (0.52 - 1.1)	0.28 (0.22 - 0.31)	0.28 (0.22 - 0.31)	0.28 (0.23 - 0.31)	0.28 (0.23 - 0.31)	0.21 (0.15 - 0.25)	0.21 (0.16 - 0.25)	0.21 (0.15 - 0.25)	0.21 (0.15 - 0.25)	1139 (1132 - 1150)	1139 (1132 - 1150)	1139 (1132 - 1150)	1139 (1132 - 1149)	1.1 (0.88 - 1.2)	1.1 (0.88 - 1.2)	1.1 (0.86 - 1.2)	1.1 (0.87 - 1.2)	0.72 (0.69 - 0.74)	0.72 (0.69 - 0.74)	0.72 (0.69 - 0.74)	0.72 (0.69 - 0.74)
	Backwards	Clin+Rad	0.72 (0.48 - 0.98)	0.71 (0.46 - 1)	0.71 (0.48 - 0.96)	0.73 (0.47 - 1)	0.29 (0.21 - 0.35)	0.28 (0.21 - 0.34)	0.29 (0.22 - 0.34)	0.3 (0.23 - 0.37)	0.23 (0.15 - 0.32)	0.23 (0.15 - 0.3)	0.23 (0.15 - 0.31)	0.25 (0.16 - 0.33)	1136 (1122 - 1152)	1137 (1125 - 1152)	1136 (1124 - 1151)	1133 (1117 - 1148)	1.1 (0.85 - 1.4)	1.1 (0.86 - 1.4)	1.1 (0.85 - 1.4)	1.2 (0.9 - 1.4)	0.72 (0.69 - 0.75)	0.72 (0.69 - 0.74)	0.72 (0.69 - 0.75)	0.73 (0.69 - 0.75)

Batch size[a]	Feature Selection[b]	Model[c]	Calibration[d]				Relative Explained Variation[d]								Relative Model Fit[d]				Discrimination[d]							
			Calibration slope (Mean, 95% CI)[e]				Nagelkerke's R2 (Mean, 95% CI)[f]				Royston and Sauerbrei's R2[f]				Akaike Information Criterion (Mean, 95% CI)[g]				Royston and Sauerbrei's D (Mean, 95% CI)[hi]				Concordance Index			
			ZS	WS	HM	RAW	ZS	WS	HM	RAW	ZS	WS	HM	RAW	ZS	WS	HM	RAW	ZS	WS	HM	RAW	ZS	WS	HM	RAW
	Forwards	Clin+Rad	0.66 (0.43 - 0.88)	0.64 (0.42 - 0.89)	0.65 (0.45 - 0.88)	0.67 (0.45 - 0.91)	0.3 (0.21 - 0.36)	0.29 (0.2 - 0.36)	0.3 (0.22 - 0.37)	0.33 (0.23 - 0.4)	0.25 (0.15 - 0.33)	0.24 (0.15 - 0.33)	0.25 (0.16 - 0.35)	0.28 (0.16 - 0.36)	1134 (1119 - 1153)	1135 (1120 - 1154)	1132 (1116 - 1151)	1126 (1108 - 1149)	1.2 (0.86 - 1.4)	1.1 (0.86 - 1.4)	1.2 (0.88 - 1.5)	1.3 (0.9 - 1.5)	0.72 (0.69 - 0.75)	0.72 (0.68 - 0.75)	0.72 (0.69 - 0.75)	0.73 (0.7 - 0.76)
	LASSO	Clin+Rad	0.68 (0.46 - 0.93)	0.67 (0.45 - 0.92)	0.68 (0.46 - 0.9)	0.69 (0.47 - 0.92)	0.29 (0.21 - 0.36)	0.29 (0.21 - 0.35)	0.3 (0.22 - 0.36)	0.33 (0.24 - 0.4)	0.24 (0.14 - 0.32)	0.23 (0.15 - 0.31)	0.25 (0.16 - 0.32)	0.28 (0.18 - 0.35)	1134 (1120 - 1153)	1136 (1121 - 1152)	1133 (1119 - 1151)	1126 (1109 - 1146)	1.2 (0.84 - 1.4)	1.1 (0.87 - 1.4)	1.2 (0.88 - 1.4)	1.3 (0.97 - 1.5)	0.72 (0.69 - 0.75)	0.72 (0.69 - 0.75)	0.73 (0.69 - 0.75)	0.73 (0.7 - 0.76)
	Random Survival Forests	Clin+Rad	0.71 (0.49 - 0.98)	0.71 (0.47 - 1)	0.71 (0.48 - 0.97)	0.7 (0.46 - 0.98)	0.29 (0.22 - 0.33)	0.28 (0.22 - 0.32)	0.28 (0.21 - 0.32)	0.27 (0.21 - 0.31)	0.24 (0.17 - 0.3)	0.23 (0.16 - 0.27)	0.22 (0.15 - 0.27)	0.21 (0.15 - 0.26)	1136 (1126 - 1151)	1137 (1128 - 1151)	1139 (1129 - 1153)	1139 (1130 - 1154)	1.1 (0.92 - 1.3)	1.1 (0.9 - 1.3)	1.1 (0.85 - 1.2)	1.1 (0.84 - 1.2)	0.73 (0.69 - 0.75)	0.72 (0.68 - 0.74)	0.72 (0.69 - 0.74)	0.72 (0.68 - 0.74)
	PCA + Clustering	Clin+Rad	0.72 (0.48 - 0.98)	0.72 (0.45 - 1)	0.71 (0.45 - 0.98)	0.71 (0.46 - 0.99)	0.3 (0.22 - 0.37)	0.29 (0.22 - 0.35)	0.29 (0.21 - 0.35)	0.29 (0.23 - 0.35)	0.24 (0.16 - 0.32)	0.24 (0.16 - 0.31)	0.24 (0.15 - 0.31)	0.24 (0.16 - 0.31)	1134 (1118 - 1150)	1134 (1121 - 1150)	1136 (1121 - 1152)	1135 (1122 - 1149)	1.2 (0.88 - 1.4)	1.1 (0.9 - 1.4)	1.1 (0.85 - 1.4)	1.2 (0.9 - 1.4)	0.72 (0.69 - 0.75)	0.72 (0.69 - 0.75)	0.72 (0.69 - 0.75)	0.73 (0.7 - 0.75)
15 - C	Clinical Features	Clinical	0.74 (0.48 - 1)	0.74 (0.48 - 1.1)	0.75 (0.49 - 1.1)	0.74 (0.49 - 1.1)	0.27 (0.21 - 0.31)	0.27 (0.21 - 0.31)	0.27 (0.21 - 0.31)	0.27 (0.21 - 0.31)	0.21 (0.15 - 0.26)	0.21 (0.14 - 0.25)	0.21 (0.15 - 0.26)	0.21 (0.14 - 0.25)	884.2 (877.7 - 895)	884.4 (877.8 - 894.8)	883.9 (877.4 - 894.8)	884.1 (877.5 - 895.3)	1.1 (0.85 - 1.2)	1.1 (0.84 - 1.2)	1.1 (0.84 - 1.2)	1.1 (0.84 - 1.2)	0.72 (0.68 - 0.74)	0.72 (0.68 - 0.74)	0.72 (0.69 - 0.74)	0.72 (0.68 - 0.74)
	Backwards	Clin+Rad	0.66 (0.41 - 0.93)	0.66 (0.42 - 0.96)	0.68 (0.41 - 0.96)	0.67 (0.42 - 0.96)	0.28 (0.2 - 0.35)	0.28 (0.2 - 0.34)	0.28 (0.2 - 0.35)	0.29 (0.2 - 0.36)	0.23 (0.14 - 0.31)	0.22 (0.14 - 0.31)	0.23 (0.13 - 0.31)	0.24 (0.14 - 0.33)	882.2 (869.6 - 896.4)	883.4 (870.8 - 896.9)	882.1 (869.2 - 896.8)	880.8 (867.2 - 896.6)	1.1 (0.82 - 1.4)	1.1 (0.82 - 1.4)	1.1 (0.78 - 1.4)	1.1 (0.84 - 1.4)	0.72 (0.68 - 0.75)	0.72 (0.68 - 0.75)	0.72 (0.68 - 0.75)	0.72 (0.69 - 0.75)
	Forwards	Clin+Rad	0.59 (0.38 - 0.82)	0.59 (0.35 - 0.84)	0.6 (0.38 - 0.84)	0.59 (0.35 - 0.85)	0.3 (0.18 - 0.38)	0.29 (0.19 - 0.38)	0.31 (0.19 - 0.4)	0.32 (0.2 - 0.4)	0.24 (0.14 - 0.34)	0.24 (0.13 - 0.35)	0.25 (0.14 - 0.36)	0.26 (0.15 - 0.36)	880 (864.7 - 899.1)	880.1 (864.1 - 898.8)	877.3 (859.3 - 898.2)	875.8 (859.8 - 897.1)	1.2 (0.81 - 1.5)	1.2 (0.8 - 1.5)	1.2 (0.82 - 1.5)	1.2 (0.86 - 1.5)	0.72 (0.68 - 0.75)	0.72 (0.68 - 0.75)	0.73 (0.69 - 0.76)	0.73 (0.69 - 0.77)
	LASSO	Clin+Rad	0.62 (0.39 - 0.87)	0.61 (0.39 - 0.87)	0.63 (0.41 - 0.87)	0.62 (0.41 - 0.87)	0.29 (0.19 - 0.37)	0.28 (0.19 - 0.37)	0.31 (0.21 - 0.39)	0.31 (0.22 - 0.39)	0.24 (0.14 - 0.33)	0.23 (0.13 - 0.34)	0.25 (0.15 - 0.35)	0.26 (0.17 - 0.35)	880.6 (866.7 - 898.3)	882.5 (865 - 898.1)	877.9 (862.5 - 894.5)	876.4 (861.2 - 893.1)	1.1 (0.81 - 1.4)	1.1 (0.8 - 1.5)	1.2 (0.85 - 1.5)	1.2 (0.91 - 1.5)	0.72 (0.69 - 0.75)	0.72 (0.68 - 0.75)	0.73 (0.69 - 0.75)	0.73 (0.69 - 0.77)
	Random Survival Forests	Clin+Rad	0.66 (0.43 - 0.93)	0.68 (0.45 - 0.97)	0.68 (0.43 - 0.95)	0.67 (0.43 - 0.98)	0.29 (0.21 - 0.34)	0.29 (0.22 - 0.34)	0.28 (0.2 - 0.33)	0.27 (0.19 - 0.32)	0.23 (0.15 - 0.29)	0.23 (0.16 - 0.29)	0.23 (0.15 - 0.29)	0.21 (0.13 - 0.27)	881.6 (871.9 - 894.7)	880.4 (871.5 - 893.7)	882.8 (873.2 - 896)	883.9 (874.8 - 897.4)	1.1 (0.87 - 1.3)	1.1 (0.89 - 1.3)	1.1 (0.85 - 1.3)	1.1 (0.8 - 1.2)	0.73 (0.69 - 0.75)	0.72 (0.68 - 0.74)	0.72 (0.68 - 0.74)	0.71 (0.67 - 0.74)
	PCA + Clustering	Clin+Rad	0.65 (0.4 - 0.92)	0.67 (0.42 - 0.96)	0.66 (0.38 - 0.93)	0.65 (0.36 - 0.95)	0.29 (0.2 - 0.35)	0.29 (0.2 - 0.35)	0.28 (0.18 - 0.34)	0.28 (0.19 - 0.34)	0.23 (0.13 - 0.31)	0.23 (0.14 - 0.3)	0.22 (0.13 - 0.3)	0.23 (0.14 - 0.31)	881.7 (869.8 - 896.9)	881.6 (870.5 - 896.2)	882.8 (871 - 899.5)	882.9 (871.6 - 898.3)	1.1 (0.8 - 1.4)	1.1 (0.82 - 1.4)	1.1 (0.81 - 1.4)	1.1 (0.83 - 1.4)	0.72 (0.69 - 0.75)	0.72 (0.69 - 0.75)	0.72 (0.68 - 0.75)	0.72 (0.68 - 0.75)

CI = Confindence Interval; HM = Histogram Matching; LASSO = Least Absolute Shrinkage and Selection Operator; PCA = Principle Component Analysis; RAW = No intensity standardisation prior to radiomic extraction; WS = WhiteStripe standardisation; ZS = Z-Score intensity standardisation

[a]Minimum number of patients in each ComBat batch for realignment of radiomic features

Batch size[a]	Feature Selection[b]	Model[c]	Calibration[d]				Relative Explained Variation[d]								Relative Model Fit[d]				Discrimination[d]							
			Calibration slope (Mean, 95% CI)[e]				Nagelkerke's R2 (Mean, 95% CI)[f]				Royston and Sauerbrei's R2[f]				Akaike Information Criterion (Mean, 95% CI)[g]				Royston and Sauerbrei's D (Mean, 95% CI)[hi]				Concordance Index			
			ZS	WS	HM	RAW	ZS	WS	HM	RAW	ZS	WS	HM	RAW	ZS	WS	HM	RAW	ZS	WS	HM	RAW	ZS	WS	HM	RAW

[b]Maximum of four radiomic features selected with the chosen method

[c]Clinical features only or a combination of both clinical and radiomic features in the Cox proportional hazards model

[d]All performance measures indicate the value derived from the 'test' sample (ie. data withheld from bootstrap resample, and not used to build initial/training model)

[e]Overall calibration slope of model - Values closer to 1 indicate a better calibration

[f]Values range from 0 to 1, with higher values suggesting higher relative explanation of variation in outcome by the model compared to other model fit using same data

[g]Lower values suggest an improvement in relative model fit compared to other models fit using same dataset

[h]Values represent prognostic separation of two equal sized groups, split by the median risk score (log hazard ratio for linear predictor of Cox model) - values further from 0 suggest better discrimination

[i]Values range from 0.5 to 1, with values closer to 1 suggesting better model discrimination

Supplementary Table 4c – Model performance statistics for calibration, relative explained variation, relative model fit and discrimination by presenting mean and 95% confidence intervals of the statistics derived from 1000 bootstrap repetitions. Results are shown for 32 bin count with ComBat realignment. The models shown here are the clinical only and combined radiomics + clinical models, built using five different feature selection processes to select the radiomic features.

Batch size[a]	Feature Selection[b]	Model[c]	Calibration[d]				Relative Explained Variation[d]								Relative Model Fit[d]				Discrimination[d]							
			Calibration slope (Mean, 95% CI)[e]				Nagelkerke's R2 (Mean, 95% CI)[f]				Royston and Sauerbrei's R2[f]				Akaike Information Criterion (Mean, 95% CI)[g]				Royston and Sauerbrei's D (Mean, 95% CI)[hi]				Concordance Index			
			ZS	WS	HM	RAW	ZS	WS	HM	RAW	ZS	WS	HM	RAW	ZS	WS	HM	RAW	ZS	WS	HM	RAW	ZS	WS	HM	RAW
5 + C	Clinical Features	Clinical	0.82 (0.59 - 1.1)	0.83 (0.6 - 1.1)	0.82 (0.6 - 1.1)	0.82 (0.6 - 1.1)	0.27 (0.23 - 0.29)	0.27 (0.22 - 0.29)	0.27 (0.22 - 0.29)	0.27 (0.23 - 0.29)	0.21 (0.17 - 0.24)	0.22 (0.17 - 0.24)	0.21 (0.16 - 0.24)	0.21 (0.17 - 0.24)	1444 (1438 - 1455)	1444 (1438 - 1457)	1445 (1438 - 1456)	1445 (1438 - 1455)	1.1 (0.92 - 1.2)	1.1 (0.92 - 1.2)	1.1 (0.91 - 1.2)	1.1 (0.93 - 1.2)	0.72 (0.69 - 0.73)	0.72 (0.69 - 0.73)	0.72 (0.69 - 0.73)	0.72 (0.69 - 0.73)
	Backwards	Clin+Rad	0.76 (0.55 - 1)	0.77 (0.54 - 1)	0.76 (0.56 - 1)	0.77 (0.56 - 1)	0.28 (0.22 - 0.32)	0.27 (0.21 - 0.32)	0.27 (0.22 - 0.32)	0.28 (0.23 - 0.32)	0.23 (0.17 - 0.28)	0.23 (0.16 - 0.27)	0.23 (0.17 - 0.27)	0.23 (0.17 - 0.28)	1442 (1430 - 1456)	1443 (1431 - 1459)	1443 (1431 - 1457)	1441 (1429 - 1455)	1.1 (0.91 - 1.3)	1.1 (0.89 - 1.3)	1.1 (0.92 - 1.3)	1.1 (0.94 - 1.3)	0.72 (0.69 - 0.74)	0.72 (0.68 - 0.74)	0.72 (0.68 - 0.74)	0.72 (0.69 - 0.74)
	Forwards	Clin+Rad	0.7 (0.51 - 0.92)	0.7 (0.48 - 0.93)	0.7 (0.51 - 0.91)	0.7 (0.5 - 0.91)	0.28 (0.21 - 0.34)	0.28 (0.2 - 0.33)	0.28 (0.21 - 0.34)	0.28 (0.22 - 0.34)	0.23 (0.16 - 0.3)	0.23 (0.16 - 0.29)	0.23 (0.16 - 0.29)	0.24 (0.17 - 0.29)	1442 (1424 - 1459)	1442 (1427 - 1461)	1442 (1426 - 1460)	1440 (1426 - 1458)	1.1 (0.9 - 1.3)	1.1 (0.89 - 1.3)	1.1 (0.89 - 1.3)	1.1 (0.93 - 1.3)	0.72 (0.68 - 0.74)	0.71 (0.68 - 0.74)	0.71 (0.68 - 0.74)	0.72 (0.69 - 0.74)
	LASSO	Clin+Rad	0.72 (0.54 - 0.94)	0.73 (0.52 - 0.96)	0.73 (0.53 - 0.97)	0.72 (0.5 - 0.93)	0.27 (0.21 - 0.32)	0.28 (0.22 - 0.33)	0.28 (0.22 - 0.33)	0.29 (0.22 - 0.33)	0.23 (0.16 - 0.28)	0.23 (0.17 - 0.28)	0.23 (0.17 - 0.28)	0.24 (0.17 - 0.29)	1443 (1430 - 1458)	1441 (1428 - 1458)	1441 (1428 - 1456)	1439 (1426 - 1457)	1.1 (0.91 - 1.3)	1.1 (0.91 - 1.3)	1.1 (0.93 - 1.3)	1.1 (0.94 - 1.3)	0.72 (0.68 - 0.74)	0.72 (0.68 - 0.74)	0.72 (0.68 - 0.74)	0.72 (0.69 - 0.74)
	Random Survival Forests	Clin+Rad	0.76 (0.55 - 1)	0.77 (0.56 - 1)	0.77 (0.55 - 1)	0.76 (0.54 - 1)	0.29 (0.24 - 0.32)	0.3 (0.24 - 0.33)	0.28 (0.22 - 0.32)	0.28 (0.23 - 0.32)	0.24 (0.19 - 0.28)	0.25 (0.19 - 0.28)	0.23 (0.16 - 0.27)	0.23 (0.18 - 0.27)	1439 (1430 - 1453)	1437 (1428 - 1451)	1442 (1432 - 1457)	1440 (1430 - 1455)	1.2 (0.98 - 1.3)	1.2 (0.99 - 1.3)	1.1 (0.91 - 1.2)	1.1 (0.94 - 1.3)	0.73 (0.7 - 0.74)	0.72 (0.7 - 0.74)	0.72 (0.69 - 0.74)	0.72 (0.69 - 0.74)
	PCA + Clustering	Clin+Rad	0.76 (0.55 - 1)	0.77 (0.55 - 1)	0.77 (0.57 - 1)	0.76 (0.55 - 1)	0.3 (0.24 - 0.34)	0.29 (0.23 - 0.34)	0.29 (0.23 - 0.33)	0.28 (0.23 - 0.32)	0.25 (0.18 - 0.31)	0.24 (0.18 - 0.3)	0.24 (0.17 - 0.29)	0.23 (0.17 - 0.28)	1437 (1423 - 1452)	1439 (1426 - 1455)	1439 (1426 - 1455)	1441 (1430 - 1455)	1.2 (0.97 - 1.4)	1.2 (0.95 - 1.3)	1.1 (0.93 - 1.3)	1.1 (0.94 - 1.3)	0.72 (0.7 - 0.74)	0.72 (0.69 - 0.74)	0.72 (0.69 - 0.74)	0.72 (0.69 - 0.74)
10 + C	Clinical Features	Clinical	0.79 (0.54 - 1.1)	0.79 (0.54 - 1.1)	0.78 (0.52 - 1)	0.79 (0.54 - 1.1)	0.28 (0.22 - 0.31)	0.28 (0.23 - 0.31)	0.28 (0.22 - 0.31)	0.28 (0.22 - 0.31)	0.21 (0.15 - 0.25)	0.22 (0.16 - 0.25)	0.21 (0.15 - 0.25)	0.21 (0.15 - 0.25)	1139 (1132 - 1150)	1139 (1132 - 1149)	1139 (1132 - 1150)	1138 (1132 - 1150)	1.1 (0.87 - 1.2)	1.1 (0.88 - 1.2)	1.1 (0.87 - 1.2)	1.1 (0.87 - 1.2)	0.72 (0.69 - 0.74)	0.72 (0.69 - 0.74)	0.72 (0.69 - 0.74)	0.72 (0.69 - 0.74)
	Backwards	Clin+Rad	0.71 (0.48 - 0.99)	0.72 (0.48 - 0.98)	0.72 (0.48 - 0.98)	0.73 (0.49 - 0.98)	0.29 (0.22 - 0.35)	0.29 (0.22 - 0.35)	0.29 (0.22 - 0.34)	0.3 (0.23 - 0.36)	0.24 (0.15 - 0.31)	0.23 (0.15 - 0.3)	0.24 (0.16 - 0.3)	0.25 (0.17 - 0.32)	1136 (1123 - 1152)	1136 (1123 - 1151)	1136 (1124 - 1150)	1133 (1119 - 1148)	1.1 (0.88 - 1.4)	1.1 (0.87 - 1.3)	1.1 (0.89 - 1.3)	1.2 (0.92 - 1.4)	0.72 (0.69 - 0.75)	0.72 (0.69 - 0.75)	0.72 (0.69 - 0.75)	0.73 (0.69 - 0.75)

Batch size[a]	Feature Selection[b]	Model[c]	Calibration[d]				Relative Explained Variation[d]								Relative Model Fit[d]				Discrimination[d]							
			Calibration slope (Mean, 95% CI)[e]				Nagelkerke's R2 (Mean, 95% CI)[f]				Royston and Sauerbrei's R2[f]				Akaike Information Criterion (Mean, 95% CI)[g]				Royston and Sauerbrei's D (Mean, 95% CI)[hi]				Concordance Index			
			ZS	WS	HM	RAW	ZS	WS	HM	RAW	ZS	WS	HM	RAW	ZS	WS	HM	RAW	ZS	WS	HM	RAW	ZS	WS	HM	RAW
	Forwards	Clin+Rad	0.66 (0.46 - 0.88)	0.64 (0.43 - 0.87)	0.65 (0.44 - 0.89)	0.66 (0.46 - 0.88)	0.31 (0.21 - 0.38)	0.29 (0.2 - 0.37)	0.3 (0.22 - 0.37)	0.31 (0.21 - 0.38)	0.26 (0.16 - 0.35)	0.24 (0.15 - 0.32)	0.25 (0.16 - 0.33)	0.26 (0.16 - 0.34)	1132 (1114 - 1153)	1135 (1118 - 1154)	1133 (1116 - 1151)	1132 (1114 - 1152)	1.2 (0.88 - 1.5)	1.1 (0.86 - 1.4)	1.2 (0.88 - 1.4)	1.2 (0.88 - 1.5)	0.73 (0.69 - 0.76)	0.72 (0.68 - 0.75)	0.73 (0.69 - 0.75)	0.73 (0.69 - 0.75)
	LASSO	Clin+Rad	0.68 (0.46 - 0.93)	0.68 (0.45 - 0.94)	0.68 (0.47 - 0.91)	0.69 (0.47 - 0.92)	0.3 (0.22 - 0.37)	0.29 (0.22 - 0.36)	0.3 (0.23 - 0.36)	0.31 (0.22 - 0.37)	0.25 (0.16 - 0.34)	0.24 (0.16 - 0.31)	0.25 (0.16 - 0.31)	0.26 (0.18 - 0.33)	1133 (1116 - 1151)	1135 (1120 - 1151)	1133 (1119 - 1148)	1131 (1117 - 1150)	1.2 (0.88 - 1.5)	1.1 (0.88 - 1.4)	1.2 (0.89 - 1.4)	1.2 (0.95 - 1.4)	0.73 (0.69 - 0.76)	0.72 (0.69 - 0.75)	0.73 (0.69 - 0.75)	0.73 (0.69 - 0.75)
	Random Survival Forests	Clin+Rad	0.71 (0.48 - 0.98)	0.72 (0.49 - 0.98)	0.72 (0.48 - 0.96)	0.71 (0.48 - 0.98)	0.29 (0.22 - 0.33)	0.29 (0.23 - 0.33)	0.28 (0.22 - 0.32)	0.27 (0.2 - 0.32)	0.24 (0.17 - 0.3)	0.23 (0.17 - 0.27)	0.22 (0.16 - 0.27)	0.21 (0.14 - 0.26)	1136 (1126 - 1150)	1136 (1127 - 1149)	1139 (1129 - 1151)	1139 (1130 - 1154)	1.2 (0.92 - 1.3)	1.1 (0.92 - 1.3)	1.1 (0.88 - 1.2)	1.1 (0.83 - 1.2)	0.73 (0.7 - 0.75)	0.72 (0.68 - 0.74)	0.72 (0.69 - 0.74)	0.72 (0.68 - 0.74)
	PCA + Clustering	Clin+Rad	0.72 (0.48 - 0.99)	0.72 (0.48 - 0.99)	0.71 (0.47 - 0.96)	0.72 (0.49 - 0.97)	0.31 (0.22 - 0.38)	0.3 (0.22 - 0.36)	0.3 (0.22 - 0.36)	0.29 (0.22 - 0.34)	0.26 (0.17 - 0.35)	0.25 (0.16 - 0.32)	0.25 (0.16 - 0.33)	0.24 (0.17 - 0.3)	1131 (1114 - 1150)	1133 (1119 - 1150)	1133 (1118 - 1150)	1135 (1124 - 1150)	1.2 (0.93 - 1.5)	1.2 (0.9 - 1.4)	1.2 (0.9 - 1.4)	1.2 (0.91 - 1.4)	0.73 (0.69 - 0.75)	0.73 (0.69 - 0.75)	0.73 (0.69 - 0.75)	0.72 (0.69 - 0.75)
15 + C	Clinical Features	Clinical	0.75 (0.48 - 1.1)	0.75 (0.48 - 1.1)	0.74 (0.47 - 1)	0.74 (0.49 - 1.1)	0.27 (0.21 - 0.31)	0.27 (0.21 - 0.31)	0.27 (0.2 - 0.31)	0.27 (0.21 - 0.31)	0.21 (0.14 - 0.26)	0.21 (0.15 - 0.25)	0.21 (0.14 - 0.26)	0.21 (0.14 - 0.25)	884 (877.1 - 895.2)	884.2 (877.6 - 894.5)	884.2 (877.7 - 896.4)	884.3 (877.7 - 895)	1.1 (0.83 - 1.2)	1.1 (0.85 - 1.2)	1.1 (0.84 - 1.2)	1.1 (0.82 - 1.2)	0.72 (0.68 - 0.74)	0.72 (0.68 - 0.74)	0.72 (0.68 - 0.74)	0.72 (0.68 - 0.74)
	Backwards	Clin+Rad	0.66 (0.42 - 0.95)	0.66 (0.43 - 0.94)	0.66 (0.42 - 0.96)	0.67 (0.42 - 0.96)	0.28 (0.21 - 0.35)	0.28 (0.2 - 0.34)	0.28 (0.2 - 0.34)	0.29 (0.2 - 0.35)	0.23 (0.14 - 0.31)	0.22 (0.14 - 0.29)	0.22 (0.14 - 0.3)	0.23 (0.14 - 0.31)	882.1 (869.2 - 895.5)	883.6 (872.7 - 896.5)	882.5 (871.2 - 896.6)	881.5 (869 - 896.6)	1.1 (0.84 - 1.4)	1.1 (0.82 - 1.3)	1.1 (0.83 - 1.3)	1.1 (0.82 - 1.4)	0.72 (0.69 - 0.75)	0.72 (0.68 - 0.75)	0.72 (0.68 - 0.75)	0.72 (0.68 - 0.75)
	Forwards	Clin+Rad	0.6 (0.36 - 0.87)	0.58 (0.35 - 0.83)	0.59 (0.36 - 0.85)	0.59 (0.36 - 0.85)	0.29 (0.18 - 0.38)	0.28 (0.18 - 0.36)	0.3 (0.19 - 0.38)	0.31 (0.19 - 0.4)	0.25 (0.13 - 0.35)	0.23 (0.13 - 0.32)	0.24 (0.14 - 0.34)	0.26 (0.14 - 0.36)	880 (863.6 - 899.7)	882.5 (867.7 - 899.9)	879.6 (863.7 - 897.9)	878 (859.5 - 898.8)	1.2 (0.81 - 1.5)	1.1 (0.79 - 1.4)	1.2 (0.82 - 1.5)	1.2 (0.82 - 1.5)	0.72 (0.68 - 0.76)	0.72 (0.68 - 0.75)	0.72 (0.68 - 0.75)	0.73 (0.68 - 0.76)
	LASSO	Clin+Rad	0.62 (0.4 - 0.88)	0.62 (0.4 - 0.89)	0.63 (0.41 - 0.9)	0.62 (0.42 - 0.87)	0.3 (0.21 - 0.36)	0.28 (0.2 - 0.35)	0.3 (0.2 - 0.37)	0.3 (0.2 - 0.38)	0.24 (0.15 - 0.32)	0.23 (0.14 - 0.3)	0.24 (0.15 - 0.32)	0.25 (0.15 - 0.33)	880 (866.9 - 895.1)	882.2 (870.5 - 897.2)	879 (866 - 895.8)	879.8 (864.1 - 896.8)	1.2 (0.86 - 1.4)	1.1 (0.82 - 1.3)	1.2 (0.86 - 1.4)	1.2 (0.87 - 1.5)	0.73 (0.69 - 0.75)	0.72 (0.68 - 0.75)	0.73 (0.69 - 0.75)	0.72 (0.68 - 0.76)
	Random Survival Forests	Clin+Rad	0.66 (0.43 - 0.93)	0.67 (0.42 - 0.97)	0.67 (0.43 - 0.96)	0.66 (0.42 - 0.93)	0.29 (0.21 - 0.34)	0.29 (0.21 - 0.35)	0.28 (0.2 - 0.33)	0.27 (0.18 - 0.33)	0.23 (0.15 - 0.29)	0.23 (0.16 - 0.29)	0.23 (0.15 - 0.29)	0.21 (0.13 - 0.27)	881.6 (872.1 - 894.9)	880.3 (870.7 - 894.2)	882.5 (873.2 - 897)	884.4 (874.5 - 899.5)	1.1 (0.87 - 1.3)	1.1 (0.89 - 1.3)	1.1 (0.84 - 1.3)	1.1 (0.78 - 1.3)	0.73 (0.69 - 0.75)	0.72 (0.69 - 0.74)	0.72 (0.68 - 0.74)	0.71 (0.68 - 0.74)
	PCA + Clustering	Clin+Rad	0.66 (0.42 - 0.96)	0.67 (0.4 - 0.95)	0.66 (0.41 - 0.95)	0.66 (0.42 - 0.93)	0.29 (0.21 - 0.36)	0.29 (0.21 - 0.35)	0.29 (0.2 - 0.35)	0.28 (0.19 - 0.35)	0.24 (0.14 - 0.31)	0.23 (0.15 - 0.3)	0.23 (0.14 - 0.3)	0.23 (0.14 - 0.31)	880.7 (867.7 - 895.6)	880.9 (870.3 - 895.7)	881.7 (869.3 - 896.8)	882.2 (870.4 - 898.1)	1.1 (0.84 - 1.4)	1.1 (0.85 - 1.3)	1.1 (0.83 - 1.4)	1.1 (0.84 - 1.4)	0.73 (0.69 - 0.75)	0.72 (0.69 - 0.75)	0.72 (0.68 - 0.75)	0.72 (0.69 - 0.75)

CI = Confindence Interval; HM = Histogram Matching; LASSO = Least Absolute Shrinkage and Selection Operator; PCA = Principle Component Analysis; RAW = No intensity standardisation prior to radiomic extraction; WS = WhiteStripe standardisation; ZS = Z-Score intensity standardisation

[a]Minimum number of patients in each ComBat batch for realignment of radiomic features

Batch size[a]	Feature Selection[b]	Model[c]	Calibration[d]				Relative Explained Variation[d]								Relative Model Fit[d]				Discrimination[d]							
			Calibration slope (Mean, 95% CI)[e]				Nagelkerke's R2 (Mean, 95% CI)[f]				Royston and Sauerbrei's R2[f]				Akaike Information Criterion (Mean, 95% CI)[g]				Royston and Sauerbrei's D (Mean, 95% CI)[hi]				Concordance Index			
			ZS	WS	HM	RAW	ZS	WS	HM	RAW	ZS	WS	HM	RAW	ZS	WS	HM	RAW	ZS	WS	HM	RAW	ZS	WS	HM	RAW

[b]Maximum of four radiomic features selected with the chosen method

[c]Clinical features only or a combination of both clinical and radiomic features in the Cox proportional hazards model

[d]All performance measures indicate the value derived from the 'test' sample (ie. data withheld from bootstrap resample, and not used to build initial/training model)

[e]Overall calibration slope of model - Values closer to 1 indicate a better calibration

[f]Values range from 0 to 1, with higher values suggesting higher relative explanation of variation in outcome by the model compared to other model fit using same data

[g]Lower values suggest an improvement in relative model fit compared to other models fit using same dataset

[h]Values represent prognostic separation of two equal sized groups, split by the median risk score (log hazard ratio for linear predictor of Cox model) - values further from 0 suggest better discrimination

[i]Values range from 0.5 to 1, with values closer to 1 suggesting better model discrimination

Supplementary Table 4d – Model performance statistics for calibration, relative explained variation, relative model fit and discrimination by presenting mean and 95% confidence intervals of the statistics derived from 1000 bootstrap repetitions. Results are shown for 32 bin count without ComBat realignment. The models shown here are the clinical only and combined radiomics + clinical models, built using five different feature selection processes to select the radiomic features.

Batch size[a]	Feature Selection[b]	Model[c]	Calibration[d]				Relative Explained Variation[d]								Relative Model Fit[d]				Discrimination[d]							
			Calibration slope (Mean, 95% CI)[e]				Nagelkerke's R2 (Mean, 95% CI)[f]				Royston and Sauerbrei's R2[f]				Akaike Information Criterion (Mean, 95% CI)[g]				Royston and Sauerbrei's D (Mean, 95% CI)[hi]				Concordance Index			
			ZS	WS	HM	RAW	ZS	WS	HM	RAW	ZS	WS	HM	RAW	ZS	WS	HM	RAW	ZS	WS	HM	RAW	ZS	WS	HM	RAW
5 - C	Clinical Features	Clinical	0.81 (0.59 - 1.1)	0.82 (0.6 - 1.1)	0.82 (0.59 - 1.1)	0.82 (0.59 - 1.1)	0.27 (0.23 - 0.29)	0.27 (0.23 - 0.29)	0.27 (0.22 - 0.29)	0.27 (0.22 - 0.29)	0.21 (0.17 - 0.24)	0.21 (0.17 - 0.25)	0.21 (0.17 - 0.25)	0.21 (0.17 - 0.24)	1445 (1438 - 1454)	1444 (1438 - 1456)	1444 (1438 - 1457)	1445 (1438 - 1457)	1.1 (0.93 - 1.2)	1.1 (0.92 - 1.2)	1.1 (0.91 - 1.2)	1.1 (0.92 - 1.2)	0.72 (0.69 - 0.73)	0.72 (0.69 - 0.73)	0.72 (0.69 - 0.73)	0.72 (0.69 - 0.73)

Batch size[a]	Feature Selection[b]	Model[c]	Calibration[d]				Relative Explained Variation[d]								Relative Model Fit[d]				Discrimination[d]							
			Calibration slope (Mean, 95% CI)[e]				Nagelkerke's R2 (Mean, 95% CI)[f]				Royston and Sauerbrei's R2[f]				Akaike Information Criterion (Mean, 95% CI)[g]				Royston and Sauerbrei's D (Mean, 95% CI)[hi]				Concordance Index			
			ZS	WS	HM	RAW	ZS	WS	HM	RAW	ZS	WS	HM	RAW	ZS	WS	HM	RAW	ZS	WS	HM	RAW	ZS	WS	HM	RAW
	Backwards	Clin+Rad	0.76 (0.55 - 1)	0.77 (0.56 - 1)	0.76 (0.54 - 1)	0.76 (0.56 - 0.99)	0.28 (0.22 - 0.32)	0.27 (0.22 - 0.32)	0.27 (0.22 - 0.32)	0.28 (0.22 - 0.32)	0.23 (0.17 - 0.28)	0.23 (0.17 - 0.27)	0.23 (0.16 - 0.27)	0.23 (0.17 - 0.28)	1442 (1430 - 1456)	1443 (1431 - 1457)	1443 (1430 - 1458)	1441 (1429 - 1457)	1.1 (0.94 - 1.3)	1.1 (0.91 - 1.3)	1.1 (0.9 - 1.2)	1.1 (0.93 - 1.3)	0.72 (0.69 - 0.74)	0.72 (0.69 - 0.74)	0.72 (0.69 - 0.74)	0.72 (0.69 - 0.74)
	Forwards	Clin+Rad	0.7 (0.51 - 0.9)	0.7 (0.49 - 0.9)	0.69 (0.5 - 0.92)	0.7 (0.49 - 0.9)	0.28 (0.21 - 0.34)	0.28 (0.21 - 0.33)	0.28 (0.21 - 0.34)	0.28 (0.22 - 0.33)	0.23 (0.16 - 0.3)	0.23 (0.16 - 0.29)	0.23 (0.16 - 0.29)	0.24 (0.17 - 0.3)	1442 (1425 - 1460)	1442 (1427 - 1458)	1442 (1425 - 1460)	1440 (1426 - 1458)	1.1 (0.89 - 1.3)	1.1 (0.91 - 1.3)	1.1 (0.88 - 1.3)	1.1 (0.91 - 1.3)	0.72 (0.68 - 0.74)	0.72 (0.68 - 0.74)	0.71 (0.68 - 0.74)	0.72 (0.69 - 0.74)
	LASSO	Clin+Rad	0.72 (0.53 - 0.95)	0.73 (0.53 - 0.94)	0.73 (0.51 - 0.96)	0.72 (0.51 - 0.94)	0.28 (0.21 - 0.32)	0.28 (0.22 - 0.33)	0.28 (0.21 - 0.32)	0.29 (0.23 - 0.34)	0.23 (0.16 - 0.28)	0.23 (0.17 - 0.28)	0.23 (0.16 - 0.28)	0.24 (0.18 - 0.29)	1442 (1429 - 1458)	1441 (1429 - 1457)	1441 (1429 - 1458)	1440 (1425 - 1455)	1.1 (0.9 - 1.3)	1.1 (0.94 - 1.3)	1.1 (0.89 - 1.3)	1.1 (0.95 - 1.3)	0.72 (0.69 - 0.74)	0.72 (0.69 - 0.74)	0.72 (0.69 - 0.74)	0.72 (0.69 - 0.74)
	Random Survival Forests	Clin+Rad	0.76 (0.55 - 1)	0.77 (0.55 - 1)	0.76 (0.54 - 1)	0.76 (0.55 - 1)	0.29 (0.23 - 0.32)	0.3 (0.24 - 0.33)	0.28 (0.21 - 0.32)	0.28 (0.23 - 0.32)	0.24 (0.19 - 0.28)	0.25 (0.19 - 0.28)	0.23 (0.17 - 0.27)	0.23 (0.18 - 0.27)	1440 (1430 - 1453)	1437 (1428 - 1451)	1442 (1432 - 1459)	1441 (1430 - 1455)	1.2 (0.98 - 1.3)	1.2 (0.99 - 1.3)	1.1 (0.91 - 1.2)	1.1 (0.94 - 1.2)	0.72 (0.7 - 0.74)	0.72 (0.7 - 0.74)	0.72 (0.69 - 0.74)	0.72 (0.69 - 0.74)
	PCA + Clustering	Clin+Rad	0.76 (0.55 - 1)	0.77 (0.55 - 1)	0.76 (0.55 - 1)	0.76 (0.55 - 1)	0.3 (0.24 - 0.35)	0.29 (0.23 - 0.33)	0.29 (0.23 - 0.33)	0.28 (0.22 - 0.32)	0.25 (0.18 - 0.31)	0.24 (0.18 - 0.29)	0.24 (0.17 - 0.29)	0.23 (0.17 - 0.28)	1437 (1422 - 1452)	1439 (1426 - 1455)	1439 (1426 - 1455)	1441 (1431 - 1457)	1.2 (0.96 - 1.4)	1.2 (0.95 - 1.3)	1.1 (0.93 - 1.3)	1.1 (0.93 - 1.3)	0.72 (0.7 - 0.74)	0.72 (0.69 - 0.74)	0.72 (0.69 - 0.74)	0.72 (0.69 - 0.74)
10 - C	Clinical Features	Clinical	0.78 (0.53 - 1.1)	0.79 (0.53 - 1.1)	0.78 (0.53 - 1.1)	0.78 (0.52 - 1.1)	0.28 (0.22 - 0.31)	0.28 (0.23 - 0.31)	0.28 (0.22 - 0.31)	0.28 (0.23 - 0.31)	0.21 (0.15 - 0.25)	0.21 (0.16 - 0.25)	0.21 (0.15 - 0.25)	0.21 (0.16 - 0.25)	1139 (1132 - 1150)	1139 (1132 - 1149)	1139 (1132 - 1150)	1138 (1132 - 1149)	1.1 (0.85 - 1.2)	1.1 (0.88 - 1.2)	1.1 (0.86 - 1.2)	1.1 (0.89 - 1.2)	0.72 (0.69 - 0.74)	0.72 (0.69 - 0.74)	0.72 (0.69 - 0.74)	0.72 (0.69 - 0.74)
	Backwards	Clin+Rad	0.71 (0.48 - 0.97)	0.72 (0.49 - 0.97)	0.71 (0.49 - 0.97)	0.72 (0.48 - 1)	0.29 (0.22 - 0.34)	0.29 (0.22 - 0.34)	0.29 (0.22 - 0.34)	0.3 (0.23 - 0.36)	0.24 (0.15 - 0.31)	0.23 (0.15 - 0.3)	0.23 (0.16 - 0.3)	0.25 (0.17 - 0.32)	1136 (1124 - 1151)	1136 (1125 - 1151)	1136 (1123 - 1151)	1133 (1119 - 1148)	1.1 (0.87 - 1.4)	1.1 (0.88 - 1.3)	1.1 (0.89 - 1.3)	1.2 (0.91 - 1.4)	0.72 (0.69 - 0.75)	0.72 (0.69 - 0.74)	0.72 (0.69 - 0.75)	0.73 (0.7 - 0.75)
	Forwards	Clin+Rad	0.66 (0.43 - 0.89)	0.65 (0.43 - 0.9)	0.64 (0.44 - 0.88)	0.66 (0.45 - 0.9)	0.31 (0.2 - 0.39)	0.29 (0.2 - 0.37)	0.3 (0.21 - 0.38)	0.31 (0.22 - 0.38)	0.26 (0.15 - 0.34)	0.24 (0.15 - 0.32)	0.25 (0.15 - 0.33)	0.26 (0.17 - 0.34)	1132 (1112 - 1154)	1135 (1118 - 1154)	1133 (1115 - 1152)	1131 (1114 - 1150)	1.2 (0.86 - 1.5)	1.1 (0.84 - 1.4)	1.2 (0.86 - 1.4)	1.2 (0.91 - 1.5)	0.73 (0.69 - 0.76)	0.72 (0.68 - 0.75)	0.73 (0.69 - 0.75)	0.73 (0.69 - 0.75)
	LASSO	Clin+Rad	0.68 (0.46 - 0.93)	0.69 (0.46 - 0.94)	0.68 (0.47 - 0.9)	0.69 (0.46 - 0.93)	0.31 (0.22 - 0.38)	0.29 (0.21 - 0.35)	0.3 (0.23 - 0.37)	0.31 (0.24 - 0.37)	0.26 (0.16 - 0.34)	0.24 (0.16 - 0.31)	0.25 (0.16 - 0.32)	0.26 (0.18 - 0.33)	1132 (1115 - 1151)	1135 (1121 - 1152)	1132 (1117 - 1149)	1131 (1116 - 1147)	1.2 (0.9 - 1.5)	1.2 (0.9 - 1.4)	1.2 (0.9 - 1.4)	1.2 (0.95 - 1.4)	0.73 (0.7 - 0.76)	0.72 (0.69 - 0.75)	0.73 (0.69 - 0.75)	0.73 (0.7 - 0.76)
	Random Survival Forests	Clin+Rad	0.71 (0.49 - 0.97)	0.73 (0.49 - 0.99)	0.71 (0.47 - 0.96)	0.71 (0.47 - 1)	0.29 (0.22 - 0.33)	0.29 (0.23 - 0.33)	0.28 (0.21 - 0.32)	0.27 (0.2 - 0.31)	0.24 (0.16 - 0.3)	0.23 (0.17 - 0.27)	0.22 (0.15 - 0.27)	0.21 (0.14 - 0.26)	1136 (1126 - 1151)	1136 (1128 - 1149)	1139 (1129 - 1152)	1139 (1130 - 1154)	1.1 (0.9 - 1.3)	1.1 (0.92 - 1.3)	1.1 (0.87 - 1.2)	1.1 (0.84 - 1.2)	0.73 (0.69 - 0.75)	0.72 (0.69 - 0.74)	0.72 (0.68 - 0.74)	0.72 (0.68 - 0.74)
	PCA + Clustering	Clin+Rad	0.72 (0.49 - 0.98)	0.73 (0.49 - 0.98)	0.71 (0.48 - 0.97)	0.71 (0.48 - 0.99)	0.31 (0.23 - 0.38)	0.3 (0.23 - 0.36)	0.3 (0.22 - 0.36)	0.29 (0.22 - 0.34)	0.26 (0.17 - 0.35)	0.25 (0.16 - 0.32)	0.25 (0.16 - 0.33)	0.24 (0.16 - 0.31)	1131 (1115 - 1148)	1132 (1119 - 1148)	1134 (1119 - 1151)	1135 (1123 - 1150)	1.2 (0.92 - 1.5)	1.2 (0.89 - 1.4)	1.2 (0.88 - 1.4)	1.2 (0.9 - 1.4)	0.73 (0.7 - 0.75)	0.73 (0.69 - 0.75)	0.73 (0.69 - 0.75)	0.73 (0.69 - 0.75)

Batch size[a]	Feature Selection[b]	Model[c]	Calibration[d]				Relative Explained Variation[d]								Relative Model Fit[d]				Discrimination[d]							
			Calibration slope (Mean, 95% CI)[e]				Nagelkerke's R2 (Mean, 95% CI)[f]				Royston and Sauerbrei's R2[f]				Akaike Information Criterion (Mean, 95% CI)[g]				Royston and Sauerbrei's D (Mean, 95% CI)[hi]				Concordance Index			
			ZS	WS	HM	RAW	ZS	WS	HM	RAW	ZS	WS	HM	RAW	ZS	WS	HM	RAW	ZS	WS	HM	RAW	ZS	WS	HM	RAW
15 - C	Clinical Features	Clinical	0.74 (0.48 - 1)	0.74 (0.47 - 1)	0.74 (0.48 - 1.1)	0.74 (0.48 - 1.1)	0.27 (0.21 - 0.31)	0.27 (0.2 - 0.31)	0.27 (0.21 - 0.31)	0.27 (0.2 - 0.31)	0.21 (0.14 - 0.26)	0.21 (0.14 - 0.26)	0.21 (0.15 - 0.25)	0.21 (0.14 - 0.26)	884.2 (877.6 - 894.6)	884.4 (877.5 - 896.1)	884.3 (877.5 - 894.2)	884.1 (877.4 - 895.9)	1.1 (0.82 - 1.2)	1 (0.82 - 1.2)	1.1 (0.85 - 1.2)	1.1 (0.83 - 1.2)	0.72 (0.68 - 0.74)	0.72 (0.68 - 0.74)	0.72 (0.68 - 0.74)	0.72 (0.68 - 0.74)
	Backwards	Clin+Rad	0.66 (0.41 - 0.96)	0.66 (0.41 - 0.91)	0.66 (0.42 - 0.95)	0.67 (0.42 - 0.97)	0.28 (0.2 - 0.35)	0.27 (0.18 - 0.34)	0.28 (0.2 - 0.34)	0.29 (0.2 - 0.35)	0.23 (0.14 - 0.3)	0.22 (0.13 - 0.29)	0.22 (0.14 - 0.3)	0.23 (0.14 - 0.31)	882.1 (869.9 - 895.7)	883.9 (872.7 - 899.1)	882.8 (871.1 - 896.6)	881.4 (869.6 - 896.3)	1.1 (0.82 - 1.4)	1.1 (0.78 - 1.3)	1.1 (0.82 - 1.3)	1.1 (0.84 - 1.4)	0.72 (0.68 - 0.75)	0.72 (0.68 - 0.74)	0.72 (0.68 - 0.75)	0.72 (0.69 - 0.75)
	Forwards	Clin+Rad	0.59 (0.35 - 0.84)	0.59 (0.35 - 0.85)	0.59 (0.35 - 0.83)	0.6 (0.36 - 0.84)	0.3 (0.18 - 0.38)	0.28 (0.18 - 0.36)	0.3 (0.19 - 0.39)	0.31 (0.19 - 0.4)	0.25 (0.13 - 0.34)	0.23 (0.13 - 0.32)	0.24 (0.13 - 0.34)	0.26 (0.15 - 0.36)	880 (863.8 - 899)	882.3 (867.4 - 899.1)	879.9 (862.6 - 897.7)	877.7 (860.1 - 898.6)	1.2 (0.8 - 1.5)	1.1 (0.79 - 1.4)	1.1 (0.8 - 1.5)	1.2 (0.85 - 1.5)	0.72 (0.68 - 0.76)	0.72 (0.68 - 0.75)	0.72 (0.69 - 0.75)	0.73 (0.68 - 0.76)
	LASSO	Clin+Rad	0.62 (0.38 - 0.88)	0.62 (0.38 - 0.87)	0.63 (0.41 - 0.88)	0.63 (0.41 - 0.87)	0.3 (0.21 - 0.37)	0.28 (0.2 - 0.34)	0.3 (0.21 - 0.37)	0.3 (0.2 - 0.38)	0.24 (0.15 - 0.32)	0.23 (0.14 - 0.29)	0.24 (0.14 - 0.32)	0.25 (0.14 - 0.33)	880 (866.6 - 895.7)	882.4 (871.2 - 897.3)	879.1 (866.5 - 895.3)	879.6 (864.5 - 896.5)	1.2 (0.85 - 1.4)	1.1 (0.83 - 1.3)	1.2 (0.84 - 1.4)	1.2 (0.83 - 1.4)	0.73 (0.69 - 0.75)	0.72 (0.68 - 0.75)	0.72 (0.69 - 0.75)	0.72 (0.68 - 0.76)
	Random Survival Forests	Clin+Rad	0.66 (0.43 - 0.94)	0.67 (0.42 - 0.94)	0.68 (0.44 - 0.94)	0.66 (0.42 - 0.94)	0.29 (0.21 - 0.34)	0.29 (0.21 - 0.34)	0.28 (0.21 - 0.34)	0.27 (0.19 - 0.32)	0.23 (0.15 - 0.29)	0.23 (0.15 - 0.29)	0.23 (0.14 - 0.29)	0.21 (0.13 - 0.27)	881.8 (872.2 - 895)	880.5 (871.3 - 894.7)	882.5 (872.5 - 895.3)	884.2 (875.1 - 898)	1.1 (0.86 - 1.3)	1.1 (0.87 - 1.3)	1.1 (0.82 - 1.3)	1.1 (0.8 - 1.3)	0.72 (0.69 - 0.75)	0.72 (0.68 - 0.74)	0.72 (0.68 - 0.74)	0.71 (0.67 - 0.74)
	PCA + Clustering	Clin+Rad	0.65 (0.41 - 0.92)	0.67 (0.4 - 0.94)	0.66 (0.4 - 0.95)	0.66 (0.41 - 0.96)	0.29 (0.2 - 0.36)	0.29 (0.2 - 0.35)	0.28 (0.19 - 0.35)	0.28 (0.2 - 0.35)	0.23 (0.14 - 0.31)	0.23 (0.14 - 0.3)	0.23 (0.13 - 0.3)	0.23 (0.15 - 0.31)	881.3 (868.7 - 896.9)	881 (870 - 896.2)	882.2 (870.2 - 897.7)	882.2 (869.9 - 897.2)	1.1 (0.81 - 1.4)	1.1 (0.82 - 1.3)	1.1 (0.8 - 1.3)	1.1 (0.85 - 1.4)	0.72 (0.68 - 0.75)	0.72 (0.69 - 0.75)	0.72 (0.69 - 0.75)	0.72 (0.68 - 0.75)

CI = Confindence Interval; HM = Histogram Matching; LASSO = Least Absolute Shrinkage and Selection Operator; PCA = Principle Component Analysis; RAW = No intensity standardisation prior to radiomic extraction; WS = WhiteStripe standardisation; ZS = Z-Score intensity standardisation

[a]Minimum number of patients in each ComBat batch for realignment of radiomic features

[b]Maximum of four radiomic features selected with the chosen method

[c]Clinical features only or a combination of both clinical and radiomic features in the Cox proportional hazards model

[d]All performance measures indicate the value derived from the 'test' sample (ie. data withheld from bootstrap resample, and not used to build initial/training model)

[e]Overall calibration slope of model - Values closer to 1 indicate a better calibration

[f]Values range from 0 to 1, with higher values suggesting higher relative explanation of variation in outcome by the model compared to other model fit using same data

[g]Lower values suggest an improvement in relative model fit compared to other models fit using same dataset

[h]Values represent prognostic separation of two equal sized groups, split by the median risk score (log hazard ratio for linear predictor of Cox model) - values further from 0 suggest better discrimination

[i]Values range from 0.5 to 1, with values closer to 1 suggesting better model discrimination

Supplementary Table 4e – Model performance statistics for calibration, relative explained variation, relative model fit and discrimination by presenting mean and 95% confidence intervals of the statistics derived from 1000 bootstrap repetitions. Results are shown for 64 bin count with ComBat realignment. The models shown here are the clinical only and combined radiomics + clinical models, built using five different feature selection processes to select the radiomic features.

Batch size[a]	Feature Selection[b]	Model[c]	Calibration[d]				Relative Explained Variation[d]								Relative Model Fit[d]				Discrimination[d]							
			Calibration slope (Mean, 95% CI)[e]				Nagelkerke's R2 (Mean, 95% CI)[f]				Royston and Sauerbrei's R2[f]				Akaike Information Criterion (Mean, 95% CI)[g]				Royston and Sauerbrei's D (Mean, 95% CI)[hi]				Concordance Index			
			ZS	WS	HM	RAW	ZS	WS	HM	RAW	ZS	WS	HM	RAW	ZS	WS	HM	RAW	ZS	WS	HM	RAW	ZS	WS	HM	RAW
5 + C	Clinical Features	Clinical	0.82 (0.6 - 1.1)	0.82 (0.6 - 1.1)	0.82 (0.59 - 1.1)	0.83 (0.59 - 1.1)	0.27 (0.23 - 0.29)	0.27 (0.23 - 0.29)	0.27 (0.22 - 0.29)	0.27 (0.23 - 0.29)	0.21 (0.16 - 0.25)	0.21 (0.17 - 0.25)	0.21 (0.17 - 0.25)	0.21 (0.16 - 0.24)	1445 (1438 - 1455)	1444 (1438 - 1455)	1445 (1438 - 1456)	1445 (1438 - 1455)	1.1 (0.91 - 1.2)	1.1 (0.92 - 1.2)	1.1 (0.91 - 1.2)	1.1 (0.91 - 1.2)	0.72 (0.69 - 0.73)	0.72 (0.69 - 0.73)	0.71 (0.69 - 0.73)	0.72 (0.69 - 0.73)
	Backwards	Clin+Rad	0.77 (0.56 - 1)	0.76 (0.55 - 1)	0.77 (0.55 - 1)	0.78 (0.55 - 1)	0.28 (0.22 - 0.32)	0.27 (0.22 - 0.32)	0.28 (0.22 - 0.32)	0.28 (0.23 - 0.33)	0.23 (0.17 - 0.28)	0.23 (0.17 - 0.28)	0.23 (0.17 - 0.27)	0.23 (0.17 - 0.28)	1442 (1429 - 1457)	1443 (1431 - 1457)	1442 (1430 - 1457)	1441 (1428 - 1455)	1.1 (0.91 - 1.3)	1.1 (0.91 - 1.3)	1.1 (0.92 - 1.3)	1.1 (0.93 - 1.3)	0.72 (0.69 - 0.74)	0.72 (0.69 - 0.74)	0.72 (0.69 - 0.74)	0.72 (0.69 - 0.74)
	Forwards	Clin+Rad	0.7 (0.5 - 0.92)	0.69 (0.46 - 0.92)	0.7 (0.48 - 0.94)	0.71 (0.5 - 0.92)	0.28 (0.2 - 0.34)	0.28 (0.21 - 0.33)	0.28 (0.2 - 0.34)	0.29 (0.22 - 0.34)	0.23 (0.15 - 0.3)	0.23 (0.16 - 0.29)	0.23 (0.15 - 0.29)	0.24 (0.16 - 0.3)	1441 (1424 - 1461)	1441 (1426 - 1460)	1441 (1426 - 1461)	1439 (1424 - 1457)	1.1 (0.88 - 1.3)	1.1 (0.88 - 1.3)	1.1 (0.86 - 1.3)	1.1 (0.89 - 1.3)	0.72 (0.68 - 0.74)	0.72 (0.69 - 0.74)	0.72 (0.68 - 0.74)	0.72 (0.69 - 0.74)
	LASSO	Clin+Rad	0.72 (0.52 - 0.95)	0.73 (0.51 - 0.95)	0.73 (0.51 - 0.95)	0.73 (0.52 - 0.94)	0.28 (0.21 - 0.33)	0.28 (0.23 - 0.33)	0.28 (0.21 - 0.33)	0.29 (0.23 - 0.34)	0.23 (0.16 - 0.29)	0.23 (0.17 - 0.28)	0.23 (0.16 - 0.28)	0.24 (0.17 - 0.29)	1441 (1427 - 1460)	1440 (1427 - 1455)	1441 (1428 - 1458)	1439 (1425 - 1455)	1.1 (0.9 - 1.3)	1.1 (0.93 - 1.3)	1.1 (0.9 - 1.3)	1.1 (0.94 - 1.3)	0.72 (0.68 - 0.74)	0.72 (0.69 - 0.74)	0.72 (0.69 - 0.74)	0.72 (0.69 - 0.75)
	Random Survival Forests	Clin+Rad	0.76 (0.55 - 1)	0.77 (0.56 - 1)	0.76 (0.55 - 1)	0.76 (0.54 - 1)	0.29 (0.23 - 0.32)	0.29 (0.24 - 0.33)	0.28 (0.22 - 0.32)	0.28 (0.23 - 0.32)	0.24 (0.18 - 0.28)	0.25 (0.19 - 0.28)	0.23 (0.16 - 0.27)	0.23 (0.18 - 0.27)	1440 (1430 - 1454)	1437 (1428 - 1451)	1442 (1431 - 1457)	1440 (1430 - 1454)	1.2 (0.97 - 1.3)	1.2 (1 - 1.3)	1.1 (0.91 - 1.2)	1.1 (0.95 - 1.2)	0.72 (0.7 - 0.74)	0.72 (0.7 - 0.74)	0.72 (0.69 - 0.74)	0.72 (0.69 - 0.74)
	PCA + Clustering	Clin+Rad	0.77 (0.56 - 1)	0.77 (0.55 - 1)	0.77 (0.55 - 1)	0.77 (0.55 - 1)	0.3 (0.24 - 0.34)	0.29 (0.23 - 0.33)	0.29 (0.23 - 0.33)	0.28 (0.23 - 0.32)	0.25 (0.19 - 0.3)	0.24 (0.18 - 0.29)	0.24 (0.18 - 0.29)	0.23 (0.18 - 0.28)	1437 (1425 - 1452)	1438 (1426 - 1453)	1438 (1427 - 1454)	1441 (1430 - 1455)	1.2 (0.98 - 1.3)	1.2 (0.97 - 1.3)	1.2 (0.95 - 1.3)	1.1 (0.95 - 1.3)	0.72 (0.7 - 0.74)	0.72 (0.7 - 0.74)	0.72 (0.69 - 0.74)	0.72 (0.69 - 0.74)
10 + C	Clinical Features	Clinical	0.77 (0.52 - 1.1)	0.79 (0.52 - 1.1)	0.79 (0.54 - 1.1)	0.78 (0.54 - 1.1)	0.28 (0.22 - 0.31)	0.28 (0.23 - 0.31)	0.28 (0.23 - 0.31)	0.28 (0.22 - 0.31)	0.21 (0.15 - 0.25)	0.21 (0.15 - 0.25)	0.22 (0.16 - 0.25)	0.21 (0.15 - 0.25)	1139 (1132 - 1151)	1138 (1132 - 1149)	1139 (1132 - 1149)	1139 (1132 - 1150)	1.1 (0.85 - 1.2)	1.1 (0.87 - 1.2)	1.1 (0.89 - 1.2)	1.1 (0.86 - 1.2)	0.72 (0.69 - 0.74)	0.72 (0.69 - 0.74)	0.72 (0.69 - 0.74)	0.72 (0.69 - 0.74)
	Backwards	Clin+Rad	0.71 (0.48	0.72 (0.47	0.72 (0.51 - 1)	0.72 (0.48 - 0.99)	0.29 (0.22	0.29 (0.22	0.29 (0.22	0.3 (0.23 - 0.36)	0.24 (0.16	0.24 (0.16	0.24 (0.16 - 0.3)	0.25 (0.16 - 0.32)	1135 (1122	1135 (1122	1136 (1123	1134 (1120 - 1149)	1.2 (0.88 - 1.4)	1.1 (0.88 - 1.4)	1.1 (0.9 - 1.4)	1.2 (0.9 - 1.4)	0.73 (0.69	0.72 (0.69	0.72 (0.69	0.73 (0.69 - 0.75)

Batch size[a]	Feature Selection[b]	Model[c]	Calibration[d]				Relative Explained Variation[d]								Relative Model Fit[d]				Discrimination[d]							
			Calibration slope (Mean, 95% CI)[e]				Nagelkerke's R2 (Mean, 95% CI)[f]				Royston and Sauerbrei's R2[f]				Akaike Information Criterion (Mean, 95% CI)[g]				Royston and Sauerbrei's D (Mean, 95% CI)[hi]				Concordance Index			
			ZS	WS	HM	RAW	ZS	WS	HM	RAW	ZS	WS	HM	RAW	ZS	WS	HM	RAW	ZS	WS	HM	RAW	ZS	WS	HM	RAW
			- 0.99)	- 0.99)			- 0.35)	- 0.35)	- 0.34)		- 0.31)	- 0.31)			- 1151)	- 1151)	- 1150)						- 0.75)	- 0.75)	- 0.74)	
	Forwards	Clin+Rad	0.66 (0.39 - 0.91)	0.64 (0.32 - 0.89)	0.66 (0.4 - 0.9)	0.66 (0.45 - 0.9)	0.31 (0.2 - 0.39)	0.29 (0.17 - 0.36)	0.3 (0.21 - 0.37)	0.31 (0.21 - 0.38)	0.26 (0.15 - 0.35)	0.24 (0.14 - 0.32)	0.26 (0.15 - 0.34)	0.26 (0.16 - 0.34)	1131 (1112 - 1154)	1136 (1119 - 1160)	1132 (1116 - 1153)	1132 (1115 - 1153)	1.2 (0.85 - 1.5)	1.1 (0.81 - 1.4)	1.2 (0.85 - 1.5)	1.2 (0.89 - 1.5)	0.73 (0.69 - 0.76)	0.72 (0.69 - 0.75)	0.73 (0.69 - 0.75)	0.73 (0.69 - 0.75)
	LASSO	Clin+Rad	0.68 (0.42 - 0.93)	0.68 (0.42 - 0.92)	0.68 (0.43 - 0.93)	0.68 (0.46 - 0.93)	0.31 (0.22 - 0.38)	0.29 (0.21 - 0.36)	0.31 (0.21 - 0.37)	0.3 (0.22 - 0.37)	0.26 (0.16 - 0.34)	0.24 (0.14 - 0.32)	0.26 (0.16 - 0.34)	0.26 (0.17 - 0.33)	1130 (1115 - 1151)	1135 (1120 - 1153)	1131 (1118 - 1152)	1132 (1117 - 1151)	1.2 (0.89 - 1.5)	1.2 (0.84 - 1.4)	1.2 (0.89 - 1.5)	1.2 (0.91 - 1.4)	0.73 (0.69 - 0.76)	0.72 (0.69 - 0.75)	0.73 (0.69 - 0.75)	0.73 (0.69 - 0.76)
	Random Survival Forests	Clin+Rad	0.7 (0.48 - 0.96)	0.72 (0.5 - 0.98)	0.72 (0.49 - 0.98)	0.71 (0.47 - 0.97)	0.29 (0.22 - 0.33)	0.29 (0.23 - 0.33)	0.28 (0.21 - 0.32)	0.27 (0.21 - 0.32)	0.24 (0.17 - 0.3)	0.23 (0.17 - 0.27)	0.22 (0.15 - 0.27)	0.21 (0.15 - 0.26)	1136 (1126 - 1150)	1136 (1128 - 1149)	1139 (1129 - 1152)	1139 (1130 - 1153)	1.1 (0.93 - 1.3)	1.1 (0.92 - 1.2)	1.1 (0.86 - 1.2)	1.1 (0.85 - 1.2)	0.73 (0.7 - 0.75)	0.72 (0.69 - 0.74)	0.72 (0.68 - 0.74)	0.72 (0.68 - 0.74)
	PCA + Clustering	Clin+Rad	0.72 (0.48 - 0.98)	0.72 (0.48 - 1)	0.73 (0.5 - 0.99)	0.71 (0.48 - 0.98)	0.31 (0.24 - 0.37)	0.31 (0.23 - 0.36)	0.3 (0.23 - 0.36)	0.29 (0.22 - 0.34)	0.26 (0.18 - 0.33)	0.26 (0.18 - 0.32)	0.25 (0.17 - 0.32)	0.24 (0.16 - 0.31)	1132 (1118 - 1147)	1131 (1120 - 1148)	1132 (1120 - 1148)	1135 (1124 - 1150)	1.2 (0.94 - 1.4)	1.2 (0.95 - 1.4)	1.2 (0.93 - 1.4)	1.2 (0.91 - 1.4)	0.73 (0.69 - 0.75)	0.73 (0.69 - 0.75)	0.73 (0.69 - 0.75)	0.72 (0.69 - 0.75)
15 + C	Clinical Features	Clinical	0.75 (0.49 - 1.1)	0.75 (0.48 - 1.1)	0.74 (0.48 - 1.1)	0.74 (0.48 - 1.1)	0.27 (0.21 - 0.31)	0.27 (0.21 - 0.31)	0.27 (0.21 - 0.31)	0.27 (0.21 - 0.31)	0.21 (0.14 - 0.25)	0.21 (0.14 - 0.26)	0.21 (0.14 - 0.26)	0.21 (0.14 - 0.26)	884.2 (877.3 - 894.6)	884.4 (877.7 - 894.8)	884.3 (877.4 - 894.9)	884.1 (877.3 - 894.8)	1.1 (0.84 - 1.2)	1.1 (0.84 - 1.2)	1.1 (0.82 - 1.2)	1.1 (0.83 - 1.2)	0.72 (0.68 - 0.74)	0.72 (0.69 - 0.74)	0.72 (0.69 - 0.74)	0.72 (0.69 - 0.74)
	Backwards	Clin+Rad	0.67 (0.4 - 0.96)	0.67 (0.43 - 0.98)	0.66 (0.42 - 0.98)	0.67 (0.42 - 0.97)	0.29 (0.2 - 0.35)	0.28 (0.2 - 0.34)	0.28 (0.2 - 0.35)	0.29 (0.2 - 0.35)	0.23 (0.14 - 0.3)	0.22 (0.14 - 0.29)	0.23 (0.14 - 0.31)	0.23 (0.14 - 0.31)	881.8 (869.8 - 896.9)	883.1 (872.1 - 896)	882.2 (869.4 - 896.2)	881.7 (869.3 - 896.4)	1.1 (0.83 - 1.4)	1.1 (0.83 - 1.3)	1.1 (0.82 - 1.4)	1.1 (0.83 - 1.4)	0.72 (0.68 - 0.75)	0.72 (0.68 - 0.75)	0.72 (0.69 - 0.75)	0.72 (0.68 - 0.75)
	Forwards	Clin+Rad	0.59 (0.3 - 0.85)	0.58 (0.31 - 0.86)	0.59 (0.31 - 0.85)	0.61 (0.36 - 0.89)	0.29 (0.17 - 0.37)	0.28 (0.17 - 0.35)	0.3 (0.17 - 0.38)	0.3 (0.2 - 0.39)	0.24 (0.13 - 0.33)	0.22 (0.13 - 0.31)	0.24 (0.13 - 0.34)	0.25 (0.15 - 0.35)	880.4 (865.5 - 901.7)	883.3 (869.8 - 901.6)	879.9 (864.5 - 901.1)	878.4 (862.6 - 896.6)	1.2 (0.79 - 1.4)	1.1 (0.78 - 1.4)	1.2 (0.79 - 1.5)	1.2 (0.85 - 1.5)	0.73 (0.68 - 0.76)	0.72 (0.68 - 0.75)	0.73 (0.69 - 0.75)	0.73 (0.69 - 0.76)
	LASSO	Clin+Rad	0.63 (0.38 - 0.89)	0.63 (0.38 - 0.91)	0.63 (0.38 - 0.87)	0.63 (0.39 - 0.9)	0.3 (0.2 - 0.36)	0.29 (0.2 - 0.35)	0.31 (0.21 - 0.38)	0.29 (0.18 - 0.37)	0.25 (0.14 - 0.32)	0.23 (0.14 - 0.3)	0.25 (0.15 - 0.34)	0.24 (0.14 - 0.33)	879 (867 - 896.2)	881.9 (870.4 - 896.1)	878.1 (864.1 - 895.2)	880.3 (865.4 - 899)	1.2 (0.83 - 1.4)	1.1 (0.84 - 1.4)	1.2 (0.84 - 1.5)	1.2 (0.81 - 1.4)	0.73 (0.69 - 0.76)	0.72 (0.69 - 0.75)	0.73 (0.7 - 0.75)	0.73 (0.68 - 0.76)
	Random Survival Forests	Clin+Rad	0.66 (0.43 - 0.96)	0.68 (0.45 - 0.97)	0.67 (0.44 - 0.96)	0.67 (0.42 - 0.95)	0.28 (0.21 - 0.34)	0.29 (0.22 - 0.34)	0.28 (0.2 - 0.34)	0.27 (0.19 - 0.33)	0.23 (0.15 - 0.29)	0.23 (0.16 - 0.29)	0.22 (0.14 - 0.29)	0.22 (0.13 - 0.27)	882.1 (872.4 - 895.5)	880.4 (871.2 - 894)	883.2 (872.8 - 896.3)	884 (874.4 - 897.9)	1.1 (0.86 - 1.3)	1.1 (0.89 - 1.3)	1.1 (0.83 - 1.3)	1.1 (0.8 - 1.3)	0.72 (0.69 - 0.75)	0.72 (0.68 - 0.74)	0.72 (0.68 - 0.74)	0.71 (0.68 - 0.74)
	PCA + Clustering	Clin+Rad	0.67 (0.42 - 0.98)	0.68 (0.41 - 0.98)	0.67 (0.39 - 0.95)	0.66 (0.41 - 0.95)	0.29 (0.21 - 0.36)	0.3 (0.22 - 0.35)	0.29 (0.21 - 0.35)	0.29 (0.2 - 0.35)	0.24 (0.15 - 0.31)	0.24 (0.15 - 0.31)	0.24 (0.15 - 0.3)	0.23 (0.14 - 0.31)	880.2 (868.8 - 894.3)	879.3 (869.1 - 893.4)	880.7 (870.2 - 895.7)	881.7 (869.8 - 896.9)	1.2 (0.87 - 1.4)	1.2 (0.87 - 1.4)	1.1 (0.85 - 1.3)	1.1 (0.81 - 1.4)	0.72 (0.69 - 0.75)	0.73 (0.69 - 0.75)	0.73 (0.69 - 0.75)	0.72 (0.69 - 0.75)

CI = Confindence Interval; HM = Histogram Matching; LASSO = Least Absolute Shrinkage and Selection Operator; PCA = Principle Component Analysis; RAW = No intensity standardisation prior to radiomic extraction; WS = WhiteStripe standardisation; ZS = Z-Score intensity standardisation

Batch size[a]	Feature Selection[b]	Model[c]	Calibration[d]				Relative Explained Variation[d]								Relative Model Fit[d]				Discrimination[d]							
			Calibration slope (Mean, 95% CI)[e]				Nagelkerke's R2 (Mean, 95% CI)[f]				Royston and Sauerbrei's R2[f]				Akaike Information Criterion (Mean, 95% CI)[g]				Royston and Sauerbrei's D (Mean, 95% CI)[hi]				Concordance Index			
			ZS	WS	HM	RAW	ZS	WS	HM	RAW	ZS	WS	HM	RAW	ZS	WS	HM	RAW	ZS	WS	HM	RAW	ZS	WS	HM	RAW

[a]Minimum number of patients in each ComBat batch for realignment of radiomic features

[b]Maximum of four radiomic features selected with the chosen method

[c]Clinical features only or a combination of both clinical and radiomic features in the Cox proportional hazards model

[d]All performance measures indicate the value derived from the 'test' sample (ie. data withheld from bootstrap resample, and not used to build initial/training model)

[e]Overall calibration slope of model - Values closer to 1 indicate a better calibration

[f]Values range from 0 to 1, with higher values suggesting higher relative explanation of variation in outcome by the model compared to other model fit using same data

[g]Lower values suggest an improvement in relative model fit compared to other models fit using same dataset

[h]Values represent prognostic separation of two equal sized groups, split by the median risk score (log hazard ratio for linear predictor of Cox model) - values further from 0 suggest better discrimination

[i]Values range from 0.5 to 1, with values closer to 1 suggesting better model discrimination

Supplementary Table 4f – Model performance statistics for calibration, relative explained variation, relative model fit and discrimination by presenting mean and 95% confidence intervals of the statistics derived from 1000 bootstrap repetitions. Results are shown for 64 bin count without ComBat realignment. The models shown here are the clinical only and combined radiomics + clinical models, built using five different feature selection processes to select the radiomic features.

Batch size[a]	Feature Selection[b]	Model[c]	Calibration[d]				Relative Explained Variation[d]								Relative Model Fit[d]				Discrimination[d]							
			Calibration slope (Mean, 95% CI)[e]				Nagelkerke's R2 (Mean, 95% CI)[f]				Royston and Sauerbrei's R2[f]				Akaike Information Criterion (Mean, 95% CI)[g]				Royston and Sauerbrei's D (Mean, 95% CI)[hi]				Concordance Index			
			ZS	WS	HM	RAW	ZS	WS	HM	RAW	ZS	WS	HM	RAW	ZS	WS	HM	RAW	ZS	WS	HM	RAW	ZS	WS	HM	RAW
5 - C	Clinical Features	Clinical	0.82 (0.6 - 1.1)	0.82 (0.59 - 1.1)	0.82 (0.6 - 1.1)	0.82 (0.58 - 1.1)	0.27 (0.22 - 0.29)	0.27 (0.23 - 0.29)	0.27 (0.23 - 0.29)	0.27 (0.23 - 0.29)	0.22 (0.17 - 0.25)	0.21 (0.17 - 0.25)	0.21 (0.17 - 0.25)	0.21 (0.17 - 0.25)	1444 (1438 - 1456)	1445 (1438 - 1455)	1444 (1438 - 1455)	1444 (1438 - 1455)	1.1 (0.91 - 1.2)	1.1 (0.91 - 1.2)	1.1 (0.92 - 1.2)	1.1 (0.92 - 1.2)	0.72 (0.69 - 0.73)	0.72 (0.69 - 0.73)	0.72 (0.68 - 0.73)	0.72 (0.69 - 0.73)

Batch size[a]	Feature Selection[b]	Model[c]	Calibration[d]				Relative Explained Variation[d]								Relative Model Fit[d]				Discrimination[d]							
			Calibration slope (Mean, 95% CI)[e]				Nagelkerke's R2 (Mean, 95% CI)[f]				Royston and Sauerbrei's R2[f]				Akaike Information Criterion (Mean, 95% CI)[g]				Royston and Sauerbrei's D (Mean, 95% CI)[hi]				Concordance Index			
			ZS	WS	HM	RAW	ZS	WS	HM	RAW	ZS	WS	HM	RAW	ZS	WS	HM	RAW	ZS	WS	HM	RAW	ZS	WS	HM	RAW
	Backwards	Clin+Rad	0.76 (0.56 - 1)	0.76 (0.55 - 1)	0.76 (0.55 - 1)	0.77 (0.56 - 0.99)	0.28 (0.22 - 0.32)	0.27 (0.22 - 0.32)	0.27 (0.22 - 0.32)	0.28 (0.23 - 0.32)	0.23 (0.17 - 0.28)	0.23 (0.17 - 0.27)	0.23 (0.17 - 0.28)	0.23 (0.18 - 0.28)	1442 (1430 - 1456)	1443 (1431 - 1457)	1443 (1431 - 1457)	1441 (1429 - 1454)	1.1 (0.94 - 1.3)	1.1 (0.92 - 1.3)	1.1 (0.93 - 1.3)	1.1 (0.96 - 1.3)	0.72 (0.69 - 0.74)	0.72 (0.69 - 0.74)	0.72 (0.68 - 0.74)	0.72 (0.69 - 0.74)
	Forwards	Clin+Rad	0.71 (0.5 - 0.94)	0.69 (0.48 - 0.92)	0.7 (0.49 - 0.92)	0.7 (0.49 - 0.92)	0.28 (0.2 - 0.34)	0.28 (0.21 - 0.33)	0.28 (0.21 - 0.34)	0.29 (0.21 - 0.34)	0.23 (0.16 - 0.3)	0.23 (0.16 - 0.29)	0.23 (0.16 - 0.29)	0.24 (0.16 - 0.29)	1441 (1424 - 1461)	1441 (1427 - 1460)	1441 (1424 - 1459)	1440 (1423 - 1458)	1.1 (0.88 - 1.3)	1.1 (0.89 - 1.3)	1.1 (0.9 - 1.3)	1.1 (0.89 - 1.3)	0.72 (0.68 - 0.74)	0.72 (0.69 - 0.74)	0.72 (0.69 - 0.74)	0.72 (0.68 - 0.74)
	LASSO	Clin+Rad	0.73 (0.55 - 0.96)	0.72 (0.52 - 0.94)	0.73 (0.53 - 0.94)	0.73 (0.51 - 0.95)	0.28 (0.22 - 0.34)	0.28 (0.22 - 0.33)	0.28 (0.22 - 0.33)	0.29 (0.23 - 0.34)	0.24 (0.17 - 0.29)	0.23 (0.17 - 0.28)	0.23 (0.16 - 0.28)	0.24 (0.17 - 0.29)	1440 (1426 - 1457)	1441 (1427 - 1456)	1441 (1429 - 1457)	1439 (1425 - 1455)	1.1 (0.93 - 1.3)	1.1 (0.93 - 1.3)	1.1 (0.91 - 1.3)	1.1 (0.94 - 1.3)	0.72 (0.69 - 0.74)	0.72 (0.69 - 0.74)	0.72 (0.69 - 0.74)	0.72 (0.69 - 0.74)
	Random Survival Forests	Clin+Rad	0.76 (0.57 - 1)	0.76 (0.56 - 1)	0.77 (0.57 - 1)	0.75 (0.54 - 0.99)	0.29 (0.23 - 0.32)	0.29 (0.24 - 0.33)	0.28 (0.22 - 0.32)	0.28 (0.23 - 0.32)	0.24 (0.18 - 0.28)	0.25 (0.19 - 0.28)	0.23 (0.17 - 0.27)	0.23 (0.18 - 0.27)	1439 (1430 - 1454)	1437 (1428 - 1451)	1442 (1431 - 1456)	1440 (1430 - 1454)	1.2 (0.97 - 1.3)	1.2 (0.99 - 1.3)	1.1 (0.94 - 1.2)	1.1 (0.95 - 1.3)	0.72 (0.7 - 0.74)	0.72 (0.7 - 0.74)	0.72 (0.69 - 0.74)	0.72 (0.69 - 0.74)
	PCA + Clustering	Clin+Rad	0.77 (0.57 - 0.99)	0.76 (0.56 - 1)	0.77 (0.55 - 1)	0.76 (0.54 - 0.99)	0.3 (0.24 - 0.34)	0.29 (0.23 - 0.34)	0.29 (0.24 - 0.33)	0.28 (0.23 - 0.32)	0.25 (0.19 - 0.3)	0.24 (0.18 - 0.29)	0.24 (0.18 - 0.29)	0.23 (0.18 - 0.28)	1437 (1425 - 1452)	1438 (1426 - 1453)	1438 (1427 - 1453)	1441 (1430 - 1454)	1.2 (1 - 1.3)	1.2 (0.97 - 1.3)	1.2 (0.97 - 1.3)	1.1 (0.95 - 1.3)	0.72 (0.7 - 0.74)	0.72 (0.7 - 0.74)	0.72 (0.69 - 0.74)	0.72 (0.69 - 0.74)
10 - C	Clinical Features	Clinical	0.78 (0.53 - 1.1)	0.78 (0.53 - 1.1)	0.79 (0.52 - 1.1)	0.77 (0.52 - 1.1)	0.28 (0.23 - 0.31)	0.28 (0.23 - 0.31)	0.28 (0.22 - 0.31)	0.28 (0.22 - 0.31)	0.21 (0.15 - 0.25)	0.21 (0.15 - 0.25)	0.21 (0.15 - 0.25)	0.21 (0.15 - 0.25)	1139 (1132 - 1149)	1138 (1132 - 1149)	1139 (1132 - 1150)	1139 (1132 - 1150)	1.1 (0.87 - 1.2)	1.1 (0.86 - 1.2)	1.1 (0.85 - 1.2)	1.1 (0.85 - 1.2)	0.72 (0.69 - 0.74)	0.72 (0.69 - 0.74)	0.72 (0.69 - 0.74)	0.72 (0.69 - 0.74)
	Backwards	Clin+Rad	0.72 (0.48 - 1)	0.72 (0.49 - 0.98)	0.72 (0.49 - 0.98)	0.72 (0.48 - 0.98)	0.29 (0.23 - 0.34)	0.29 (0.21 - 0.34)	0.29 (0.22 - 0.34)	0.3 (0.23 - 0.36)	0.24 (0.16 - 0.31)	0.24 (0.15 - 0.3)	0.24 (0.15 - 0.31)	0.25 (0.16 - 0.32)	1135 (1123 - 1149)	1136 (1124 - 1152)	1136 (1123 - 1150)	1134 (1120 - 1149)	1.1 (0.88 - 1.4)	1.1 (0.88 - 1.3)	1.1 (0.86 - 1.4)	1.2 (0.89 - 1.4)	0.72 (0.69 - 0.75)	0.72 (0.69 - 0.74)	0.72 (0.69 - 0.75)	0.73 (0.69 - 0.75)
	Forwards	Clin+Rad	0.66 (0.37 - 0.92)	0.65 (0.33 - 0.92)	0.66 (0.39 - 0.9)	0.67 (0.45 - 0.9)	0.31 (0.2 - 0.39)	0.29 (0.18 - 0.37)	0.3 (0.2 - 0.37)	0.31 (0.21 - 0.37)	0.26 (0.15 - 0.35)	0.24 (0.14 - 0.33)	0.26 (0.15 - 0.34)	0.26 (0.16 - 0.33)	1132 (1112 - 1155)	1135 (1116 - 1159)	1133 (1116 - 1155)	1132 (1116 - 1152)	1.2 (0.86 - 1.5)	1.2 (0.81 - 1.4)	1.2 (0.85 - 1.5)	1.2 (0.89 - 1.4)	0.73 (0.69 - 0.76)	0.72 (0.69 - 0.75)	0.73 (0.69 - 0.75)	0.73 (0.69 - 0.75)
	LASSO	Clin+Rad	0.68 (0.41 - 0.93)	0.68 (0.45 - 0.93)	0.69 (0.44 - 0.93)	0.69 (0.48 - 0.91)	0.31 (0.21 - 0.38)	0.3 (0.21 - 0.36)	0.31 (0.22 - 0.37)	0.3 (0.23 - 0.36)	0.26 (0.16 - 0.35)	0.25 (0.15 - 0.32)	0.26 (0.15 - 0.34)	0.26 (0.17 - 0.33)	1131 (1113 - 1152)	1134 (1120 - 1152)	1131 (1117 - 1151)	1132 (1118 - 1149)	1.2 (0.88 - 1.5)	1.2 (0.87 - 1.4)	1.2 (0.87 - 1.5)	1.2 (0.93 - 1.4)	0.73 (0.7 - 0.76)	0.72 (0.69 - 0.75)	0.73 (0.7 - 0.75)	0.73 (0.69 - 0.75)
	Random Survival Forests	Clin+Rad	0.71 (0.49 - 0.99)	0.72 (0.5 - 0.97)	0.72 (0.47 - 0.98)	0.7 (0.46 - 0.98)	0.29 (0.23 - 0.33)	0.29 (0.22 - 0.33)	0.27 (0.22 - 0.32)	0.27 (0.2 - 0.32)	0.24 (0.17 - 0.3)	0.23 (0.16 - 0.27)	0.22 (0.15 - 0.27)	0.21 (0.14 - 0.26)	1136 (1126 - 1149)	1136 (1127 - 1150)	1139 (1130 - 1152)	1139 (1130 - 1154)	1.1 (0.93 - 1.3)	1.1 (0.91 - 1.3)	1.1 (0.87 - 1.2)	1.1 (0.84 - 1.2)	0.73 (0.69 - 0.75)	0.72 (0.69 - 0.74)	0.72 (0.68 - 0.74)	0.72 (0.68 - 0.74)
	PCA + Clustering	Clin+Rad	0.72 (0.49 - 0.99)	0.73 (0.49 - 0.98)	0.73 (0.48 - 1)	0.71 (0.47 - 0.97)	0.31 (0.23 - 0.37)	0.31 (0.24 - 0.36)	0.3 (0.23 - 0.36)	0.29 (0.22 - 0.34)	0.26 (0.16 - 0.33)	0.26 (0.17 - 0.32)	0.25 (0.17 - 0.32)	0.24 (0.16 - 0.31)	1132 (1118 - 1149)	1131 (1119 - 1148)	1132 (1120 - 1148)	1135 (1123 - 1150)	1.2 (0.91 - 1.4)	1.2 (0.93 - 1.4)	1.2 (0.93 - 1.4)	1.2 (0.9 - 1.4)	0.73 (0.69 - 0.75)	0.73 (0.69 - 0.75)	0.73 (0.69 - 0.75)	0.72 (0.69 - 0.75)

Batch size[a]	Feature Selection[b]	Model[c]	Calibration[d]				Relative Explained Variation[d]								Relative Model Fit[d]				Discrimination[d]							
			Calibration slope (Mean, 95% CI)[e]				Nagelkerke's R2 (Mean, 95% CI)[f]				Royston and Sauerbrei's R2[f]				Akaike Information Criterion (Mean, 95% CI)[g]				Royston and Sauerbrei's D (Mean, 95% CI)[hi]				Concordance Index			
			ZS	WS	HM	RAW	ZS	WS	HM	RAW	ZS	WS	HM	RAW	ZS	WS	HM	RAW	ZS	WS	HM	RAW	ZS	WS	HM	RAW
15 - C	Clinical Features	Clinical	0.75 (0.48 - 1.1)	0.74 (0.48 - 1.1)	0.74 (0.48 - 1.1)	0.75 (0.48 - 1.1)	0.27 (0.21 - 0.31)	0.27 (0.21 - 0.31)	0.27 (0.21 - 0.31)	0.27 (0.21 - 0.31)	0.21 (0.14 - 0.26)	0.21 (0.15 - 0.26)	0.21 (0.14 - 0.26)	0.21 (0.14 - 0.26)	884.3 (877.7 - 894.8)	884 (877.6 - 895)	884.4 (877.6 - 895.7)	884.1 (877.7 - 894.9)	1.1 (0.83 - 1.2)	1.1 (0.86 - 1.2)	1.1 (0.82 - 1.2)	1.1 (0.84 - 1.2)	0.72 (0.68 - 0.74)	0.72 (0.68 - 0.74)	0.72 (0.68 - 0.74)	0.72 (0.68 - 0.74)
	Backwards	Clin+Rad	0.67 (0.42 - 0.95)	0.66 (0.41 - 0.97)	0.66 (0.41 - 0.95)	0.68 (0.43 - 0.97)	0.29 (0.2 - 0.35)	0.28 (0.2 - 0.34)	0.28 (0.19 - 0.35)	0.29 (0.21 - 0.35)	0.23 (0.14 - 0.31)	0.22 (0.13 - 0.3)	0.23 (0.13 - 0.3)	0.24 (0.14 - 0.32)	881.7 (869.4 - 896.1)	882.9 (871.8 - 897)	882.3 (870.7 - 898.6)	881.3 (870.1 - 895)	1.1 (0.83 - 1.4)	1.1 (0.81 - 1.3)	1.1 (0.8 - 1.3)	1.1 (0.84 - 1.4)	0.72 (0.69 - 0.75)	0.72 (0.68 - 0.75)	0.72 (0.68 - 0.75)	0.72 (0.69 - 0.75)
	Forwards	Clin+Rad	0.59 (0.31 - 0.86)	0.58 (0.31 - 0.84)	0.59 (0.32 - 0.85)	0.61 (0.38 - 0.88)	0.29 (0.16 - 0.37)	0.28 (0.16 - 0.35)	0.29 (0.17 - 0.38)	0.3 (0.2 - 0.39)	0.24 (0.12 - 0.33)	0.22 (0.12 - 0.31)	0.24 (0.13 - 0.33)	0.25 (0.14 - 0.35)	880.9 (866.2 - 902.6)	883.3 (869.2 - 902.8)	880.3 (864.6 - 901)	878.8 (861.8 - 897.3)	1.1 (0.76 - 1.4)	1.1 (0.77 - 1.4)	1.1 (0.79 - 1.4)	1.2 (0.84 - 1.5)	0.73 (0.68 - 0.75)	0.72 (0.68 - 0.75)	0.73 (0.69 - 0.76)	0.73 (0.68 - 0.76)
	LASSO	Clin+Rad	0.62 (0.35 - 0.87)	0.62 (0.39 - 0.9)	0.64 (0.36 - 0.88)	0.63 (0.4 - 0.92)	0.3 (0.19 - 0.37)	0.28 (0.19 - 0.34)	0.3 (0.2 - 0.37)	0.29 (0.2 - 0.37)	0.24 (0.14 - 0.33)	0.23 (0.14 - 0.3)	0.25 (0.14 - 0.33)	0.24 (0.15 - 0.33)	879.5 (866.8 - 897.8)	882 (870.9 - 897.7)	878.4 (865 - 896.4)	880.4 (866.4 - 896.5)	1.2 (0.83 - 1.4)	1.1 (0.82 - 1.3)	1.2 (0.84 - 1.4)	1.2 (0.85 - 1.4)	0.73 (0.69 - 0.76)	0.72 (0.68 - 0.75)	0.73 (0.69 - 0.75)	0.73 (0.69 - 0.75)
	Random Survival Forests	Clin+Rad	0.65 (0.43 - 0.93)	0.68 (0.44 - 0.97)	0.67 (0.45 - 0.94)	0.67 (0.44 - 0.95)	0.28 (0.2 - 0.33)	0.3 (0.22 - 0.35)	0.28 (0.2 - 0.33)	0.27 (0.19 - 0.32)	0.23 (0.15 - 0.28)	0.23 (0.16 - 0.29)	0.22 (0.14 - 0.29)	0.21 (0.14 - 0.28)	882.5 (872.9 - 896.2)	880.1 (870.8 - 893.8)	883.1 (873.6 - 896.7)	884 (874.8 - 897.4)	1.1 (0.85 - 1.3)	1.1 (0.88 - 1.3)	1.1 (0.84 - 1.3)	1.1 (0.82 - 1.3)	0.72 (0.69 - 0.75)	0.72 (0.69 - 0.74)	0.72 (0.68 - 0.74)	0.71 (0.68 - 0.74)
	PCA + Clustering	Clin+Rad	0.67 (0.42 - 0.93)	0.68 (0.42 - 0.96)	0.66 (0.41 - 0.95)	0.66 (0.42 - 0.95)	0.29 (0.21 - 0.36)	0.3 (0.22 - 0.36)	0.29 (0.2 - 0.35)	0.28 (0.2 - 0.35)	0.24 (0.15 - 0.32)	0.25 (0.15 - 0.31)	0.23 (0.13 - 0.3)	0.23 (0.14 - 0.31)	880.3 (868.7 - 895.5)	879.1 (868.5 - 893.6)	881 (869.6 - 896.9)	882 (870.4 - 896.7)	1.2 (0.84 - 1.4)	1.2 (0.88 - 1.4)	1.1 (0.81 - 1.4)	1.1 (0.82 - 1.4)	0.72 (0.69 - 0.75)	0.73 (0.69 - 0.75)	0.72 (0.68 - 0.75)	0.72 (0.68 - 0.75)

CI = Confindence Interval; HM = Histogram Matching; LASSO = Least Absolute Shrinkage and Selection Operator; PCA = Principle Component Analysis; RAW = No intensity standardisation prior to radiomic extraction; WS = WhiteStripe standardisation; ZS = Z-Score intensity standardisation

[a]Minimum number of patients in each ComBat batch for realignment of radiomic features

[b]Maximum of four radiomic features selected with the chosen method

[c]Clinical features only or a combination of both clinical and radiomic features in the Cox proportional hazards model

[d]All performance measures indicate the value derived from the 'test' sample (ie. data withheld from bootstrap resample, and not used to build initial/training model)

[e]Overall calibration slope of model - Values closer to 1 indicate a better calibration

[f]Values range from 0 to 1, with higher values suggesting higher relative explanation of variation in outcome by the model compared to other model fit using same data

[g]Lower values suggest an improvement in relative model fit compared to other models fit using same dataset

[h]Values represent prognostic separation of two equal sized groups, split by the median risk score (log hazard ratio for linear predictor of Cox model) - values further from 0 suggest better discrimination

[i]Values range from 0.5 to 1, with values closer to 1 suggesting better model discrimination

Supplementary Table 4g – Model performance statistics for calibration, relative explained variation, relative model fit and discrimination by presenting mean and 95% confidence intervals of the statistics derived from 1000 bootstrap repetitions. Results are shown for 128 bin count with ComBat realignment. The models shown here are the clinical only and combined radiomics + clinical models, built using five different feature selection processes to select the radiomic features.

Batch size[a]	Feature Selection[b]	Model[c]	Calibration[d]				Relative Explained Variation[d]								Relative Model Fit[d]				Discrimination[d]							
			Calibration slope (Mean, 95% CI)[e]				Nagelkerke's R2 (Mean, 95% CI)[f]				Royston and Sauerbrei's R2[f]				Akaike Information Criterion (Mean, 95% CI)[g]				Royston and Sauerbrei's D (Mean, 95% CI)[hi]				Concordance Index			
			ZS	WS	HM	RAW	ZS	WS	HM	RAW	ZS	WS	HM	RAW	ZS	WS	HM	RAW	ZS	WS	HM	RAW	ZS	WS	HM	RAW
5 + C	Clinical Features	Clinical	0.82 (0.6 - 1.1)	0.82 (0.6 - 1.1)	0.83 (0.58 - 1.1)	0.82 (0.61 - 1.1)	0.27 (0.23 - 0.29)	0.27 (0.23 - 0.29)	0.27 (0.23 - 0.29)	0.27 (0.23 - 0.29)	0.22 (0.17 - 0.24)	0.21 (0.17 - 0.25)	0.22 (0.17 - 0.25)	0.22 (0.17 - 0.25)	1444 (1438 - 1454)	1444 (1438 - 1455)	1444 (1438 - 1455)	1445 (1438 - 1455)	1.1 (0.93 - 1.2)	1.1 (0.92 - 1.2)	1.1 (0.92 - 1.2)	1.1 (0.92 - 1.2)	0.72 (0.69 - 0.73)	0.72 (0.69 - 0.73)	0.72 (0.69 - 0.73)	0.72 (0.69 - 0.73)
	Backwards	Clin+Rad	0.76 (0.55 - 1)	0.76 (0.55 - 1)	0.77 (0.55 - 1)	0.76 (0.55 - 0.99)	0.27 (0.22 - 0.31)	0.27 (0.22 - 0.31)	0.27 (0.22 - 0.31)	0.28 (0.22 - 0.33)	0.23 (0.17 - 0.27)	0.22 (0.16 - 0.27)	0.23 (0.17 - 0.27)	0.23 (0.17 - 0.28)	1443 (1432 - 1457)	1443 (1433 - 1458)	1443 (1433 - 1456)	1441 (1429 - 1456)	1.1 (0.93 - 1.3)	1.1 (0.89 - 1.2)	1.1 (0.92 - 1.2)	1.1 (0.93 - 1.3)	0.72 (0.69 - 0.74)	0.71 (0.68 - 0.73)	0.72 (0.69 - 0.74)	0.72 (0.69 - 0.74)
	Forwards	Clin+Rad	0.71 (0.5 - 0.94)	0.7 (0.48 - 0.93)	0.7 (0.49 - 0.93)	0.7 (0.48 - 0.93)	0.27 (0.2 - 0.32)	0.27 (0.2 - 0.32)	0.27 (0.2 - 0.33)	0.28 (0.2 - 0.34)	0.22 (0.15 - 0.27)	0.23 (0.15 - 0.28)	0.23 (0.14 - 0.28)	0.23 (0.16 - 0.29)	1444 (1431 - 1462)	1443 (1429 - 1462)	1443 (1429 - 1461)	1440 (1425 - 1461)	1.1 (0.87 - 1.3)	1.1 (0.88 - 1.3)	1.1 (0.84 - 1.3)	1.1 (0.9 - 1.3)	0.71 (0.68 - 0.74)	0.72 (0.68 - 0.74)	0.71 (0.68 - 0.74)	0.72 (0.69 - 0.74)
	LASSO	Clin+Rad	0.73 (0.53 - 0.95)	0.73 (0.53 - 0.98)	0.73 (0.53 - 0.97)	0.72 (0.51 - 0.93)	0.27 (0.21 - 0.31)	0.28 (0.22 - 0.32)	0.27 (0.22 - 0.31)	0.29 (0.22 - 0.34)	0.23 (0.16 - 0.27)	0.23 (0.17 - 0.27)	0.23 (0.17 - 0.27)	0.24 (0.17 - 0.29)	1443 (1432 - 1458)	1442 (1431 - 1457)	1443 (1432 - 1457)	1439 (1426 - 1457)	1.1 (0.91 - 1.2)	1.1 (0.93 - 1.3)	1.1 (0.93 - 1.2)	1.1 (0.94 - 1.3)	0.71 (0.69 - 0.74)	0.72 (0.69 - 0.74)	0.71 (0.69 - 0.73)	0.72 (0.69 - 0.75)
	Random Survival Forests	Clin+Rad	0.76 (0.55 - 1)	0.77 (0.57 - 1)	0.77 (0.55 - 1)	0.76 (0.54 - 0.99)	0.28 (0.23 - 0.32)	0.3 (0.24 - 0.33)	0.28 (0.22 - 0.31)	0.28 (0.23 - 0.32)	0.24 (0.18 - 0.28)	0.25 (0.2 - 0.28)	0.23 (0.17 - 0.27)	0.23 (0.17 - 0.27)	1440 (1431 - 1453)	1437 (1428 - 1451)	1442 (1432 - 1456)	1441 (1430 - 1455)	1.2 (0.97 - 1.3)	1.2 (1 - 1.3)	1.1 (0.93 - 1.2)	1.1 (0.94 - 1.2)	0.72 (0.7 - 0.74)	0.72 (0.7 - 0.74)	0.72 (0.69 - 0.74)	0.72 (0.69 - 0.74)
	PCA + Clustering	Clin+Rad	0.76 (0.56 - 1)	0.77 (0.56 - 1)	0.76 (0.54 - 1)	0.75 (0.55 - 0.99)	0.29 (0.24 - 0.34)	0.29 (0.23 - 0.33)	0.29 (0.23 - 0.33)	0.28 (0.22 - 0.32)	0.25 (0.18 - 0.3)	0.24 (0.18 - 0.29)	0.24 (0.18 - 0.29)	0.23 (0.17 - 0.28)	1437 (1426 - 1452)	1439 (1427 - 1455)	1440 (1427 - 1454)	1442 (1431 - 1456)	1.2 (0.97 - 1.3)	1.2 (0.95 - 1.3)	1.1 (0.94 - 1.3)	1.1 (0.93 - 1.3)	0.72 (0.7 - 0.74)	0.72 (0.69 - 0.74)	0.72 (0.69 - 0.74)	0.72 (0.69 - 0.74)
10 + C	Clinical Features	Clinical	0.77 (0.52 - 1.1)	0.78 (0.53 - 1.1)	0.79 (0.54 - 1.1)	0.78 (0.53 - 1.1)	0.28 (0.22 - 0.31)	0.28 (0.23 - 0.31)	0.28 (0.23 - 0.31)	0.28 (0.22 - 0.31)	0.21 (0.15 - 0.25)	0.21 (0.15 - 0.25)	0.22 (0.16 - 0.25)	0.21 (0.15 - 0.25)	1139 (1132 - 1151)	1139 (1132 - 1149)	1138 (1132 - 1149)	1139 (1132 - 1150)	1.1 (0.85 - 1.2)	1.1 (0.87 - 1.2)	1.1 (0.89 - 1.2)	1.1 (0.86 - 1.2)	0.72 (0.69 - 0.74)	0.72 (0.69 - 0.74)	0.72 (0.69 - 0.74)	0.72 (0.69 - 0.74)
	Backwards	Clin+Rad	0.71 (0.47	0.71 (0.47	0.72 (0.5 - 0.98)	0.72 (0.48 - 0.98)	0.29 (0.22	0.29 (0.22	0.29 (0.22	0.3 (0.23 - 0.36)	0.24 (0.15	0.23 (0.15 - 0.3)	0.24 (0.16 - 0.3)	0.25 (0.16 - 0.32)	1136 (1123	1137 (1124	1136 (1124	1134 (1119 - 1149)	1.1 (0.84 - 1.4)	1.1 (0.87 - 1.3)	1.1 (0.88 - 1.3)	1.2 (0.89 - 1.4)	0.72 (0.69	0.72 (0.69	0.72 (0.69	0.73 (0.69 - 0.75)

Batch size[a]	Feature Selection[b]	Model[c]	Calibration[d]				Relative Explained Variation[d]								Relative Model Fit[d]				Discrimination[d]							
			Calibration slope (Mean, 95% CI)[e]				Nagelkerke's R2 (Mean, 95% CI)[f]				Royston and Sauerbrei's R2[f]				Akaike Information Criterion (Mean, 95% CI)[g]				Royston and Sauerbrei's D (Mean, 95% CI)[hi]				Concordance Index			
			ZS	WS	HM	RAW	ZS	WS	HM	RAW	ZS	WS	HM	RAW	ZS	WS	HM	RAW	ZS	WS	HM	RAW	ZS	WS	HM	RAW
			- 0.97)	- 0.98)			- 0.35)	- 0.34)	- 0.34)		- 0.31)				- 1151)	- 1151)	- 1151)						- 0.75)	- 0.74)	- 0.74)	
	Forwards	Clin+Rad	0.65 (0.42 - 0.88)	0.64 (0.4 - 0.9)	0.65 (0.4 - 0.88)	0.66 (0.44 - 0.89)	0.29 (0.2 - 0.35)	0.28 (0.19 - 0.34)	0.29 (0.2 - 0.36)	0.31 (0.21 - 0.38)	0.24 (0.14 - 0.31)	0.23 (0.14 - 0.3)	0.24 (0.13 - 0.32)	0.26 (0.16 - 0.33)	1135 (1121 - 1155)	1138 (1124 - 1156)	1135 (1120 - 1155)	1131 (1114 - 1152)	1.2 (0.83 - 1.4)	1.1 (0.83 - 1.3)	1.2 (0.8 - 1.4)	1.2 (0.88 - 1.4)	0.72 (0.69 - 0.75)	0.72 (0.68 - 0.75)	0.72 (0.69 - 0.75)	0.73 (0.69 - 0.76)
	LASSO	Clin+Rad	0.68 (0.44 - 0.92)	0.68 (0.43 - 0.93)	0.68 (0.46 - 0.91)	0.69 (0.46 - 0.91)	0.3 (0.21 - 0.35)	0.28 (0.21 - 0.33)	0.3 (0.22 - 0.35)	0.32 (0.22 - 0.38)	0.25 (0.15 - 0.31)	0.23 (0.15 - 0.29)	0.24 (0.15 - 0.31)	0.26 (0.16 - 0.33)	1134 (1122 - 1152)	1137 (1126 - 1153)	1134 (1122 - 1151)	1130 (1115 - 1150)	1.2 (0.86 - 1.4)	1.1 (0.86 - 1.3)	1.2 (0.86 - 1.4)	1.2 (0.9 - 1.4)	0.73 (0.69 - 0.75)	0.72 (0.68 - 0.74)	0.73 (0.69 - 0.75)	0.73 (0.7 - 0.76)
	Random Survival Forests	Clin+Rad	0.7 (0.48 - 0.96)	0.72 (0.5 - 0.99)	0.72 (0.49 - 0.99)	0.7 (0.47 - 0.97)	0.29 (0.22 - 0.33)	0.29 (0.23 - 0.33)	0.27 (0.21 - 0.32)	0.27 (0.21 - 0.32)	0.24 (0.16 - 0.3)	0.23 (0.17 - 0.27)	0.22 (0.15 - 0.27)	0.21 (0.15 - 0.26)	1136 (1126 - 1150)	1136 (1127 - 1149)	1139 (1129 - 1153)	1140 (1130 - 1153)	1.1 (0.91 - 1.3)	1.1 (0.93 - 1.2)	1.1 (0.86 - 1.2)	1.1 (0.85 - 1.2)	0.73 (0.69 - 0.75)	0.72 (0.69 - 0.74)	0.72 (0.68 - 0.74)	0.72 (0.68 - 0.74)
	PCA + Clustering	Clin+Rad	0.72 (0.48 - 0.97)	0.72 (0.49 - 0.99)	0.72 (0.48 - 0.99)	0.7 (0.47 - 0.96)	0.31 (0.23 - 0.37)	0.3 (0.23 - 0.35)	0.3 (0.23 - 0.36)	0.29 (0.21 - 0.34)	0.26 (0.17 - 0.33)	0.25 (0.17 - 0.32)	0.25 (0.17 - 0.33)	0.24 (0.16 - 0.3)	1132 (1117 - 1148)	1133 (1121 - 1148)	1134 (1119 - 1149)	1136 (1125 - 1152)	1.2 (0.93 - 1.4)	1.2 (0.93 - 1.4)	1.2 (0.92 - 1.4)	1.1 (0.88 - 1.3)	0.73 (0.7 - 0.75)	0.73 (0.7 - 0.75)	0.72 (0.69 - 0.75)	0.72 (0.69 - 0.75)
15 + C	Clinical Features	Clinical	0.75 (0.48 - 1.1)	0.75 (0.49 - 1.1)	0.74 (0.49 - 1)	0.74 (0.49 - 1.1)	0.27 (0.21 - 0.31)	0.27 (0.21 - 0.31)	0.27 (0.21 - 0.31)	0.27 (0.21 - 0.31)	0.21 (0.14 - 0.25)	0.21 (0.15 - 0.26)	0.21 (0.14 - 0.26)	0.21 (0.14 - 0.26)	884.3 (877.6 - 895.4)	884.4 (877.7 - 895)	884.4 (877.4 - 895.7)	884.2 (877.5 - 894.9)	1.1 (0.84 - 1.2)	1.1 (0.87 - 1.2)	1 (0.82 - 1.2)	1.1 (0.83 - 1.2)	0.72 (0.69 - 0.74)	0.72 (0.68 - 0.74)	0.72 (0.68 - 0.74)	0.72 (0.68 - 0.74)
	Backwards	Clin+Rad	0.66 (0.42 - 0.97)	0.67 (0.43 - 0.97)	0.66 (0.43 - 0.96)	0.67 (0.41 - 0.95)	0.28 (0.2 - 0.34)	0.27 (0.19 - 0.33)	0.28 (0.2 - 0.34)	0.29 (0.2 - 0.35)	0.23 (0.14 - 0.3)	0.22 (0.14 - 0.3)	0.22 (0.14 - 0.3)	0.23 (0.14 - 0.31)	882.6 (871 - 897)	883.8 (873.1 - 897.3)	882.8 (871.2 - 896.6)	881.4 (869.1 - 896.8)	1.1 (0.83 - 1.3)	1.1 (0.82 - 1.3)	1.1 (0.83 - 1.3)	1.1 (0.84 - 1.4)	0.72 (0.68 - 0.75)	0.72 (0.68 - 0.75)	0.72 (0.68 - 0.75)	0.72 (0.68 - 0.75)
	Forwards	Clin+Rad	0.6 (0.35 - 0.88)	0.59 (0.35 - 0.87)	0.6 (0.36 - 0.86)	0.6 (0.37 - 0.89)	0.29 (0.18 - 0.36)	0.28 (0.17 - 0.36)	0.29 (0.2 - 0.37)	0.3 (0.19 - 0.38)	0.24 (0.13 - 0.32)	0.22 (0.11 - 0.31)	0.24 (0.14 - 0.33)	0.25 (0.14 - 0.33)	881.5 (868 - 899.4)	883.2 (868.4 - 900.9)	880.3 (866 - 896.9)	879 (864.7 - 898.8)	1.1 (0.8 - 1.4)	1.1 (0.74 - 1.4)	1.2 (0.81 - 1.4)	1.2 (0.82 - 1.4)	0.72 (0.68 - 0.75)	0.72 (0.68 - 0.75)	0.72 (0.68 - 0.75)	0.73 (0.69 - 0.76)
	LASSO	Clin+Rad	0.63 (0.39 - 0.91)	0.63 (0.39 - 0.9)	0.63 (0.4 - 0.91)	0.63 (0.4 - 0.9)	0.29 (0.2 - 0.35)	0.28 (0.19 - 0.34)	0.3 (0.22 - 0.37)	0.3 (0.21 - 0.37)	0.24 (0.14 - 0.32)	0.23 (0.13 - 0.3)	0.25 (0.15 - 0.33)	0.25 (0.16 - 0.32)	881 (869.1 - 897)	882.5 (871.1 - 897.9)	878.4 (865.9 - 893.8)	878.7 (866.4 - 894.5)	1.1 (0.84 - 1.4)	1.1 (0.8 - 1.4)	1.2 (0.87 - 1.4)	1.2 (0.89 - 1.4)	0.73 (0.69 - 0.75)	0.72 (0.68 - 0.75)	0.73 (0.69 - 0.75)	0.73 (0.68 - 0.76)
	Random Survival Forests	Clin+Rad	0.66 (0.42 - 0.94)	0.68 (0.45 - 0.96)	0.67 (0.43 - 0.94)	0.67 (0.41 - 0.95)	0.28 (0.2 - 0.33)	0.29 (0.22 - 0.34)	0.28 (0.2 - 0.33)	0.27 (0.19 - 0.33)	0.23 (0.15 - 0.29)	0.24 (0.16 - 0.29)	0.22 (0.14 - 0.28)	0.21 (0.13 - 0.27)	882.4 (873 - 896.1)	880.3 (871.2 - 893.5)	883.7 (873.9 - 897)	884.4 (874.3 - 898.8)	1.1 (0.85 - 1.3)	1.1 (0.89 - 1.3)	1.1 (0.82 - 1.3)	1.1 (0.79 - 1.3)	0.72 (0.69 - 0.75)	0.72 (0.68 - 0.74)	0.72 (0.68 - 0.74)	0.71 (0.68 - 0.74)
	PCA + Clustering	Clin+Rad	0.67 (0.4 - 0.95)	0.67 (0.42 - 0.95)	0.66 (0.4 - 0.93)	0.66 (0.39 - 0.94)	0.29 (0.21 - 0.35)	0.29 (0.21 - 0.35)	0.29 (0.21 - 0.35)	0.28 (0.19 - 0.34)	0.24 (0.15 - 0.31)	0.24 (0.15 - 0.31)	0.23 (0.14 - 0.3)	0.23 (0.14 - 0.3)	880.8 (869.7 - 895.2)	880.7 (869.8 - 895.5)	881.4 (870.3 - 895.1)	883 (871.3 - 897.8)	1.2 (0.85 - 1.4)	1.1 (0.86 - 1.4)	1.1 (0.83 - 1.4)	1.1 (0.82 - 1.3)	0.72 (0.69 - 0.75)	0.72 (0.69 - 0.75)	0.72 (0.69 - 0.75)	0.72 (0.69 - 0.75)

CI = Confindence Interval; HM = Histogram Matching; LASSO = Least Absolute Shrinkage and Selection Operator; PCA = Principle Component Analysis; RAW = No intensity standardisation prior to radiomic extraction; WS = WhiteStripe standardisation; ZS = Z-Score intensity standardisation

Batch size[a]	Feature Selection[b]	Model[c]	Calibration[d]				Relative Explained Variation[d]								Relative Model Fit[d]				Discrimination[d]							
			Calibration slope (Mean, 95% CI)[e]				Nagelkerke's R2 (Mean, 95% CI)[f]				Royston and Sauerbrei's R2[f]				Akaike Information Criterion (Mean, 95% CI)[g]				Royston and Sauerbrei's D (Mean, 95% CI)[hi]				Concordance Index			
			ZS	WS	HM	RAW	ZS	WS	HM	RAW	ZS	WS	HM	RAW	ZS	WS	HM	RAW	ZS	WS	HM	RAW	ZS	WS	HM	RAW

[a]Minimum number of patients in each ComBat batch for realignment of radiomic features

[b]Maximum of four radiomic features selected with the chosen method

[c]Clinical features only or a combination of both clinical and radiomic features in the Cox proportional hazards model

[d]All performance measures indicate the value derived from the 'test' sample (ie. data withheld from bootstrap resample, and not used to build initial/training model)

[e]Overall calibration slope of model - Values closer to 1 indicate a better calibration

[f]Values range from 0 to 1, with higher values suggesting higher relative explanation of variation in outcome by the model compared to other model fit using same data

[g]Lower values suggest an improvement in relative model fit compared to other models fit using same dataset

[h]Values represent prognostic separation of two equal sized groups, split by the median risk score (log hazard ratio for linear predictor of Cox model) - values further from 0 suggest better discrimination

[i]Values range from 0.5 to 1, with values closer to 1 suggesting better model discrimination

Supplementary Table 4h – Model performance statistics for calibration, relative explained variation, relative model fit and discrimination by presenting mean and 95% confidence intervals of the statistics derived from 1000 bootstrap repetitions. Results are shown for 128 bin count without ComBat realignment. The models shown here are the clinical only and combined radiomics + clinical models, built using five different feature selection processes to select the radiomic features.

Batch size[a]	Feature Selection[b]	Model[c]	Calibration[d]				Relative Explained Variation[d]								Relative Model Fit[d]				Discrimination[d]							
			Calibration slope (Mean, 95% CI)[e]				Nagelkerke's R2 (Mean, 95% CI)[f]				Royston and Sauerbrei's R2[f]				Akaike Information Criterion (Mean, 95% CI)[g]				Royston and Sauerbrei's D (Mean, 95% CI)[hi]				Concordance Index			
			ZS	WS	HM	RAW	ZS	WS	HM	RAW	ZS	WS	HM	RAW	ZS	WS	HM	RAW	ZS	WS	HM	RAW	ZS	WS	HM	RAW
5 - C	Clinical Features	Clinical	0.83 (0.6 - 1.1)	0.82 (0.59 - 1.1)	0.82 (0.6 - 1.1)	0.82 (0.59 - 1.1)	0.27 (0.23 - 0.29)	0.27 (0.22 - 0.29)	0.27 (0.22 - 0.29)	0.27 (0.23 - 0.29)	0.21 (0.17 - 0.24)	0.21 (0.17 - 0.24)	0.22 (0.17 - 0.25)	0.21 (0.17 - 0.25)	1445 (1438 - 1455)	1444 (1438 - 1456)	1444 (1438 - 1456)	1444 (1438 - 1455)	1.1 (0.91 - 1.2)	1.1 (0.92 - 1.2)	1.1 (0.92 - 1.2)	1.1 (0.91 - 1.2)	0.72 (0.69 - 0.73)	0.72 (0.69 - 0.73)	0.72 (0.69 - 0.73)	0.72 (0.69 - 0.73)
	Backwards	Clin+Rad	0.77 (0.55 - 1)	0.76 (0.54 - 1)	0.76 (0.55 - 1)	0.77 (0.55 - 1)	0.27 (0.22 - 0.31)	0.27 (0.22 - 0.31)	0.27 (0.22 - 0.31)	0.28 (0.23 - 0.32)	0.23 (0.16 - 0.27)	0.22 (0.16 - 0.27)	0.22 (0.17 - 0.27)	0.23 (0.18 - 0.28)	1443 (1432 - 1457)	1443 (1433 - 1458)	1443 (1433 - 1457)	1441 (1429 - 1455)	1.1 (0.91 - 1.3)	1.1 (0.9 - 1.2)	1.1 (0.92 - 1.2)	1.1 (0.94 - 1.3)	0.72 (0.69 - 0.74)	0.71 (0.69 - 0.74)	0.72 (0.69 - 0.74)	0.72 (0.69 - 0.74)
	Forwards	Clin+Rad	0.71 (0.5 - 0.94)	0.7 (0.49 - 0.92)	0.71 (0.5 - 0.94)	0.7 (0.47 - 0.91)	0.27 (0.2 - 0.32)	0.27 (0.2 - 0.32)	0.28 (0.21 - 0.33)	0.28 (0.2 - 0.34)	0.22 (0.15 - 0.28)	0.23 (0.15 - 0.28)	0.23 (0.16 - 0.28)	0.23 (0.16 - 0.29)	1444 (1430 - 1462)	1443 (1429 - 1461)	1442 (1427 - 1460)	1441 (1426 - 1461)	1.1 (0.87 - 1.3)	1.1 (0.87 - 1.3)	1.1 (0.89 - 1.3)	1.1 (0.9 - 1.3)	0.71 (0.68 - 0.74)	0.72 (0.68 - 0.74)	0.71 (0.68 - 0.74)	0.72 (0.69 - 0.74)
	LASSO	Clin+Rad	0.74 (0.52 - 0.98)	0.73 (0.5 - 0.99)	0.73 (0.52 - 0.95)	0.73 (0.51 - 0.94)	0.27 (0.21 - 0.31)	0.27 (0.2 - 0.32)	0.28 (0.21 - 0.32)	0.29 (0.22 - 0.33)	0.22 (0.16 - 0.27)	0.23 (0.16 - 0.27)	0.23 (0.16 - 0.27)	0.24 (0.17 - 0.28)	1444 (1432 - 1459)	1443 (1431 - 1461)	1442 (1431 - 1459)	1440 (1426 - 1456)	1.1 (0.88 - 1.2)	1.1 (0.89 - 1.3)	1.1 (0.9 - 1.3)	1.1 (0.94 - 1.3)	0.72 (0.69 - 0.74)	0.72 (0.69 - 0.74)	0.71 (0.68 - 0.74)	0.72 (0.69 - 0.74)
	Random Survival Forests	Clin+Rad	0.77 (0.57 - 1)	0.77 (0.56 - 1)	0.77 (0.56 - 1)	0.76 (0.54 - 1)	0.28 (0.23 - 0.32)	0.29 (0.24 - 0.33)	0.28 (0.22 - 0.32)	0.28 (0.23 - 0.32)	0.24 (0.18 - 0.29)	0.25 (0.19 - 0.28)	0.23 (0.17 - 0.27)	0.23 (0.18 - 0.27)	1440 (1430 - 1454)	1437 (1428 - 1451)	1442 (1432 - 1457)	1441 (1431 - 1454)	1.2 (0.97 - 1.3)	1.2 (1 - 1.3)	1.1 (0.93 - 1.2)	1.1 (0.95 - 1.2)	0.72 (0.7 - 0.74)	0.72 (0.7 - 0.74)	0.72 (0.69 - 0.74)	0.72 (0.69 - 0.74)
	PCA + Clustering	Clin+Rad	0.77 (0.56 - 1)	0.76 (0.53 - 1)	0.76 (0.55 - 1)	0.76 (0.53 - 1)	0.29 (0.24 - 0.33)	0.29 (0.22 - 0.33)	0.29 (0.23 - 0.33)	0.28 (0.22 - 0.32)	0.25 (0.18 - 0.3)	0.24 (0.17 - 0.29)	0.24 (0.17 - 0.29)	0.23 (0.17 - 0.28)	1438 (1426 - 1453)	1439 (1428 - 1457)	1440 (1428 - 1455)	1442 (1430 - 1457)	1.2 (0.97 - 1.3)	1.2 (0.94 - 1.3)	1.1 (0.93 - 1.3)	1.1 (0.94 - 1.3)	0.72 (0.7 - 0.74)	0.72 (0.69 - 0.74)	0.72 (0.69 - 0.74)	0.72 (0.69 - 0.74)
10 - C	Clinical Features	Clinical	0.78 (0.53 - 1.1)	0.78 (0.52 - 1.1)	0.78 (0.53 - 1.1)	0.78 (0.52 - 1.1)	0.28 (0.23 - 0.31)	0.27 (0.22 - 0.31)	0.28 (0.23 - 0.31)	0.28 (0.22 - 0.31)	0.21 (0.15 - 0.25)	0.21 (0.14 - 0.25)	0.21 (0.15 - 0.25)	0.21 (0.15 - 0.25)	1138 (1132 - 1149)	1139 (1132 - 1151)	1139 (1132 - 1149)	1139 (1132 - 1150)	1.1 (0.88 - 1.2)	1.1 (0.84 - 1.2)	1.1 (0.87 - 1.2)	1.1 (0.87 - 1.2)	0.72 (0.69 - 0.74)	0.72 (0.69 - 0.74)	0.72 (0.69 - 0.74)	0.72 (0.69 - 0.74)
	Backwards	Clin+Rad	0.71 (0.48 - 0.96)	0.72 (0.48 - 0.98)	0.71 (0.49 - 0.99)	0.72 (0.48 - 0.98)	0.29 (0.22 - 0.34)	0.28 (0.22 - 0.33)	0.29 (0.22 - 0.34)	0.3 (0.23 - 0.36)	0.24 (0.16 - 0.3)	0.23 (0.16 - 0.3)	0.24 (0.15 - 0.3)	0.25 (0.17 - 0.32)	1135 (1124 - 1151)	1137 (1126 - 1151)	1136 (1125 - 1150)	1133 (1119 - 1149)	1.1 (0.88 - 1.3)	1.1 (0.88 - 1.3)	1.1 (0.87 - 1.3)	1.2 (0.92 - 1.4)	0.72 (0.69 - 0.75)	0.72 (0.69 - 0.74)	0.72 (0.69 - 0.74)	0.73 (0.7 - 0.75)

Batch size[a]	Feature Selection[b]	Model[c]	Calibration[d]				Relative Explained Variation[d]								Relative Model Fit[d]				Discrimination[d]							
			Calibration slope (Mean, 95% CI)[e]				Nagelkerke's R2 (Mean, 95% CI)[f]				Royston and Sauerbrei's R2[f]				Akaike Information Criterion (Mean, 95% CI)[g]				Royston and Sauerbrei's D (Mean, 95% CI)[hi]				Concordance Index			
			ZS	WS	HM	RAW	ZS	WS	HM	RAW	ZS	WS	HM	RAW	ZS	WS	HM	RAW	ZS	WS	HM	RAW	ZS	WS	HM	RAW
	Forwards	Clin+Rad	0.66 (0.41 - 0.91)	0.64 (0.4 - 0.88)	0.64 (0.43 - 0.88)	0.66 (0.44 - 0.89)	0.29 (0.2 - 0.36)	0.28 (0.18 - 0.34)	0.29 (0.2 - 0.36)	0.31 (0.21 - 0.38)	0.24 (0.14 - 0.31)	0.23 (0.14 - 0.29)	0.24 (0.14 - 0.32)	0.26 (0.16 - 0.34)	1135 (1120 - 1155)	1138 (1123 - 1158)	1135 (1120 - 1154)	1131 (1115 - 1152)	1.2 (0.82 - 1.4)	1.1 (0.81 - 1.3)	1.2 (0.84 - 1.4)	1.2 (0.91 - 1.5)	0.72 (0.69 - 0.75)	0.72 (0.68 - 0.74)	0.72 (0.69 - 0.75)	0.73 (0.69 - 0.76)
	LASSO	Clin+Rad	0.68 (0.45 - 0.93)	0.68 (0.45 - 0.92)	0.67 (0.46 - 0.9)	0.69 (0.48 - 0.94)	0.3 (0.22 - 0.35)	0.28 (0.2 - 0.33)	0.29 (0.22 - 0.35)	0.32 (0.22 - 0.38)	0.24 (0.15 - 0.31)	0.23 (0.15 - 0.29)	0.24 (0.16 - 0.3)	0.27 (0.18 - 0.33)	1134 (1122 - 1151)	1137 (1126 - 1154)	1134 (1123 - 1151)	1130 (1115 - 1150)	1.2 (0.88 - 1.4)	1.1 (0.85 - 1.3)	1.2 (0.88 - 1.4)	1.2 (0.96 - 1.4)	0.73 (0.69 - 0.75)	0.72 (0.68 - 0.74)	0.72 (0.69 - 0.75)	0.73 (0.69 - 0.76)
	Random Survival Forests	Clin+Rad	0.71 (0.48 - 0.97)	0.72 (0.48 - 0.98)	0.71 (0.5 - 0.97)	0.7 (0.47 - 0.99)	0.29 (0.22 - 0.34)	0.28 (0.22 - 0.33)	0.28 (0.21 - 0.32)	0.27 (0.21 - 0.31)	0.24 (0.17 - 0.3)	0.23 (0.16 - 0.27)	0.22 (0.15 - 0.27)	0.21 (0.14 - 0.26)	1136 (1125 - 1150)	1137 (1127 - 1151)	1139 (1129 - 1152)	1140 (1130 - 1153)	1.1 (0.92 - 1.3)	1.1 (0.89 - 1.2)	1.1 (0.86 - 1.2)	1.1 (0.83 - 1.2)	0.73 (0.69 - 0.75)	0.72 (0.68 - 0.74)	0.72 (0.68 - 0.74)	0.72 (0.68 - 0.74)
	PCA + Clustering	Clin+Rad	0.72 (0.49 - 0.99)	0.71 (0.49 - 0.96)	0.71 (0.48 - 0.96)	0.71 (0.48 - 0.98)	0.31 (0.23 - 0.37)	0.3 (0.22 - 0.36)	0.3 (0.22 - 0.35)	0.29 (0.22 - 0.34)	0.26 (0.17 - 0.33)	0.25 (0.16 - 0.32)	0.25 (0.16 - 0.32)	0.24 (0.16 - 0.3)	1131 (1117 - 1149)	1134 (1120 - 1150)	1134 (1121 - 1150)	1135 (1125 - 1150)	1.2 (0.93 - 1.4)	1.2 (0.88 - 1.4)	1.2 (0.89 - 1.4)	1.2 (0.9 - 1.3)	0.73 (0.7 - 0.75)	0.72 (0.69 - 0.75)	0.73 (0.69 - 0.75)	0.72 (0.69 - 0.75)
15 - C	Clinical Features	Clinical	0.75 (0.49 - 1)	0.74 (0.47 - 1.1)	0.74 (0.48 - 1.1)	0.74 (0.48 - 1)	0.27 (0.21 - 0.31)	0.27 (0.21 - 0.31)	0.27 (0.21 - 0.31)	0.27 (0.21 - 0.31)	0.21 (0.14 - 0.26)	0.21 (0.14 - 0.26)	0.21 (0.14 - 0.25)	0.21 (0.15 - 0.26)	884 (877.4 - 894.4)	884.4 (877.6 - 895.7)	883.9 (877.6 - 894.5)	884.2 (877.4 - 894.6)	1.1 (0.84 - 1.2)	1.1 (0.83 - 1.2)	1.1 (0.84 - 1.2)	1.1 (0.84 - 1.2)	0.72 (0.68 - 0.74)	0.72 (0.68 - 0.74)	0.72 (0.69 - 0.74)	0.72 (0.69 - 0.74)
	Backwards	Clin+Rad	0.66 (0.41 - 0.94)	0.66 (0.42 - 0.94)	0.66 (0.43 - 0.96)	0.67 (0.41 - 0.93)	0.28 (0.19 - 0.35)	0.27 (0.18 - 0.33)	0.28 (0.2 - 0.34)	0.29 (0.2 - 0.35)	0.23 (0.14 - 0.3)	0.22 (0.13 - 0.29)	0.23 (0.14 - 0.3)	0.23 (0.14 - 0.31)	882.5 (870.5 - 897.3)	883.8 (873.2 - 899.1)	882.6 (871.1 - 896.1)	881.6 (869.5 - 896.9)	1.1 (0.83 - 1.4)	1.1 (0.8 - 1.3)	1.1 (0.81 - 1.3)	1.1 (0.83 - 1.4)	0.72 (0.68 - 0.75)	0.72 (0.68 - 0.74)	0.72 (0.68 - 0.75)	0.72 (0.69 - 0.75)
	Forwards	Clin+Rad	0.61 (0.37 - 0.87)	0.58 (0.34 - 0.87)	0.6 (0.38 - 0.85)	0.6 (0.35 - 0.86)	0.29 (0.18 - 0.37)	0.28 (0.16 - 0.36)	0.3 (0.2 - 0.37)	0.3 (0.19 - 0.37)	0.24 (0.13 - 0.33)	0.22 (0.12 - 0.31)	0.24 (0.13 - 0.33)	0.25 (0.14 - 0.33)	881.1 (866.2 - 899.7)	883.4 (868.9 - 902.8)	880 (865.9 - 897.3)	879.5 (865 - 897.4)	1.1 (0.79 - 1.4)	1.1 (0.75 - 1.4)	1.2 (0.8 - 1.5)	1.2 (0.82 - 1.4)	0.72 (0.68 - 0.75)	0.72 (0.67 - 0.75)	0.72 (0.69 - 0.75)	0.73 (0.69 - 0.76)
	LASSO	Clin+Rad	0.63 (0.39 - 0.9)	0.63 (0.39 - 0.9)	0.64 (0.39 - 0.92)	0.63 (0.41 - 0.91)	0.29 (0.2 - 0.36)	0.28 (0.19 - 0.34)	0.31 (0.21 - 0.37)	0.3 (0.2 - 0.37)	0.24 (0.15 - 0.32)	0.23 (0.14 - 0.3)	0.25 (0.15 - 0.33)	0.25 (0.15 - 0.32)	880.5 (868.3 - 897)	882.4 (871 - 897.3)	878.2 (866 - 895.3)	879 (866.2 - 896.9)	1.2 (0.86 - 1.4)	1.1 (0.81 - 1.3)	1.2 (0.84 - 1.5)	1.2 (0.86 - 1.4)	0.73 (0.69 - 0.75)	0.72 (0.68 - 0.75)	0.73 (0.69 - 0.75)	0.73 (0.69 - 0.76)
	Random Survival Forests	Clin+Rad	0.66 (0.43 - 0.95)	0.68 (0.42 - 0.96)	0.67 (0.41 - 0.95)	0.67 (0.43 - 0.92)	0.29 (0.21 - 0.34)	0.29 (0.21 - 0.34)	0.28 (0.21 - 0.33)	0.27 (0.19 - 0.32)	0.23 (0.16 - 0.29)	0.23 (0.15 - 0.29)	0.22 (0.14 - 0.28)	0.21 (0.13 - 0.27)	881.9 (872 - 895.6)	880.5 (871.1 - 895.3)	883.2 (873.6 - 895.5)	884.3 (874.8 - 897.9)	1.1 (0.88 - 1.3)	1.1 (0.88 - 1.3)	1.1 (0.83 - 1.3)	1.1 (0.79 - 1.3)	0.73 (0.69 - 0.75)	0.72 (0.68 - 0.74)	0.72 (0.68 - 0.74)	0.71 (0.68 - 0.74)
	PCA + Clustering	Clin+Rad	0.67 (0.41 - 0.96)	0.67 (0.42 - 0.97)	0.67 (0.4 - 0.95)	0.66 (0.4 - 0.93)	0.29 (0.2 - 0.36)	0.29 (0.2 - 0.35)	0.29 (0.21 - 0.35)	0.28 (0.19 - 0.34)	0.24 (0.14 - 0.32)	0.24 (0.15 - 0.31)	0.24 (0.15 - 0.31)	0.23 (0.14 - 0.3)	880.5 (868.8 - 896.5)	881 (869.7 - 895.9)	881.1 (870.7 - 894.5)	882.6 (871 - 897.9)	1.2 (0.82 - 1.4)	1.1 (0.85 - 1.4)	1.1 (0.85 - 1.4)	1.1 (0.82 - 1.3)	0.72 (0.69 - 0.75)	0.72 (0.68 - 0.75)	0.72 (0.69 - 0.75)	0.72 (0.69 - 0.75)

CI = Confindence Interval; HM = Histogram Matching; LASSO = Least Absolute Shrinkage and Selection Operator; PCA = Principle Component Analysis; RAW = No intensity standardisation prior to radiomic extraction; WS = WhiteStripe standardisation; ZS = Z-Score intensity standardisation

[a]Minimum number of patients in each ComBat batch for realignment of radiomic features

Batch size[a]	Feature Selection[b]	Model[c]	Calibration[d]				Relative Explained Variation[d]								Relative Model Fit[d]				Discrimination[d]							
			Calibration slope (Mean, 95% CI)[e]				Nagelkerke's R2 (Mean, 95% CI)[f]				Royston and Sauerbrei's R2[f]				Akaike Information Criterion (Mean, 95% CI)[g]				Royston and Sauerbrei's D (Mean, 95% CI)[hi]				Concordance Index			
			ZS	WS	HM	RAW	ZS	WS	HM	RAW	ZS	WS	HM	RAW	ZS	WS	HM	RAW	ZS	WS	HM	RAW	ZS	WS	HM	RAW

[b]Maximum of four radiomic features selected with the chosen method

[c]Clinical features only or a combination of both clinical and radiomic features in the Cox proportional hazards model

[d]All performance measures indicate the value derived from the 'test' sample (ie. data withheld from bootstrap resample, and not used to build initial/training model)

[e]Overall calibration slope of model - Values closer to 1 indicate a better calibration

[f]Values range from 0 to 1, with higher values suggesting higher relative explanation of variation in outcome by the model compared to other model fit using same data

[g]Lower values suggest an improvement in relative model fit compared to other models fit using same dataset

[h]Values represent prognostic separation of two equal sized groups, split by the median risk score (log hazard ratio for linear predictor of Cox model) - values further from 0 suggest better discrimination

[i]Values range from 0.5 to 1, with values closer to 1 suggesting better model discrimination

***Supplementary Figure 1a** – Stacked barcharts demonstrating the different ComBat batch labels per MRI sequence, for minimum batch size = 5. Each bar represents a different MRI sequence. Each segment of a bar represents a unique batch label (see key for details).*

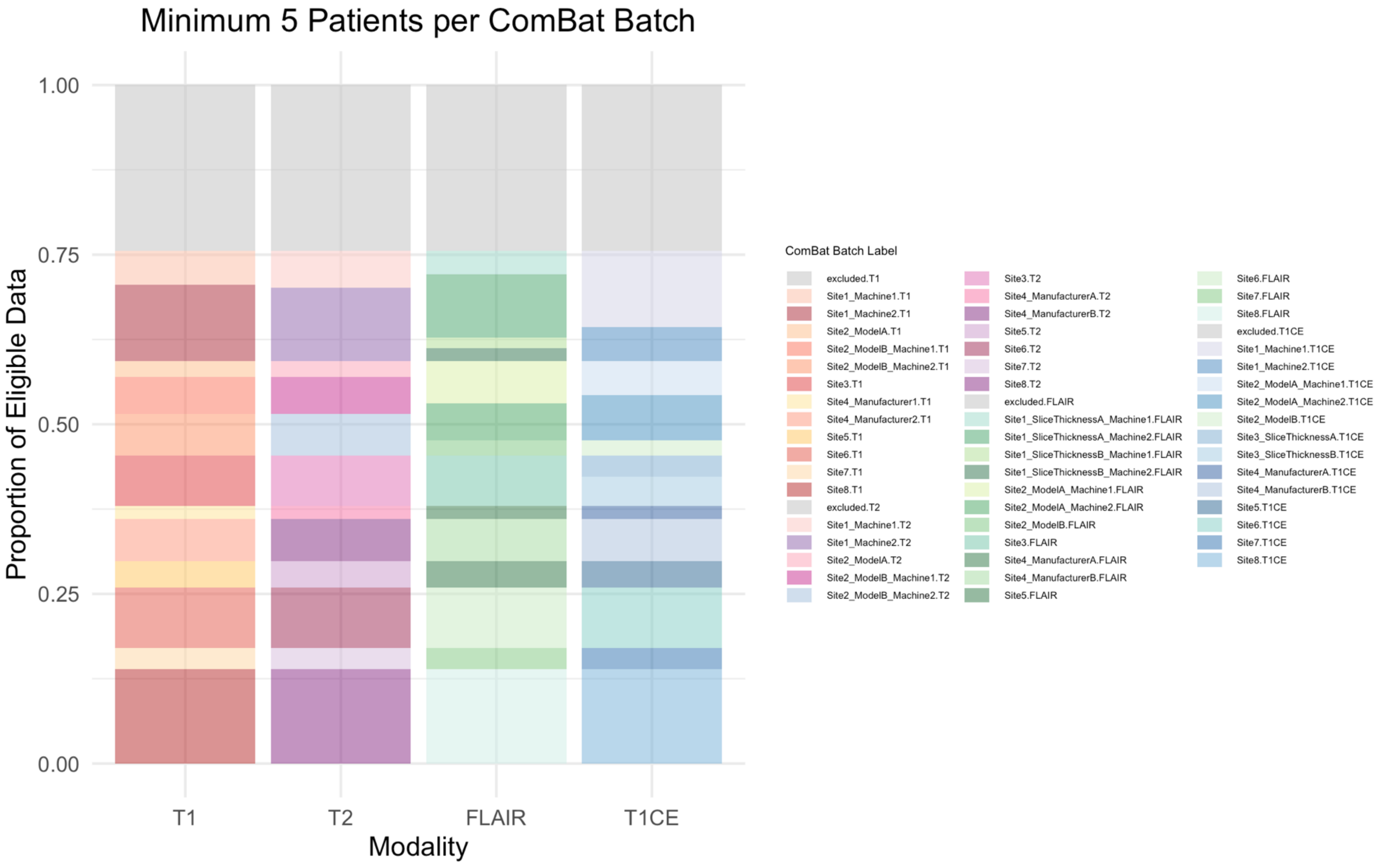

Supplementary Figure 1b *– Stacked barcharts demonstrating the different ComBat batch labels per MRI sequence, for minimum batch size = 10. Each bar represents a different MRI sequence. Each segment of a bar represents a unique batch label (see key for details).*

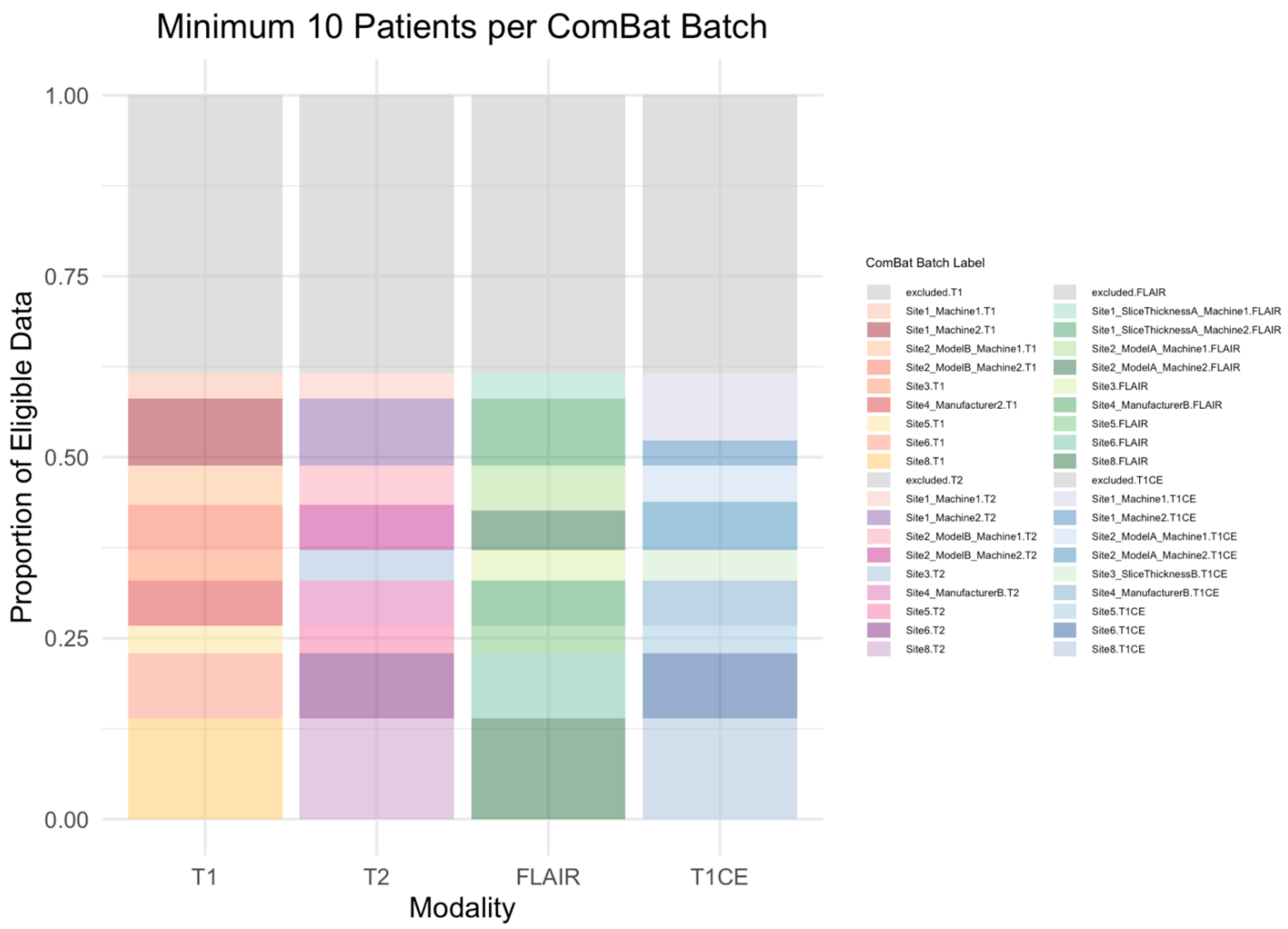

Supplementary Figure 1c *– Stacked barcharts demonstrating the different ComBat batch labels per MRI sequence, for minimum batch size = 10. Each bar represents a different MRI sequence. Each segment of a bar represents a unique batch label (see key for details).*

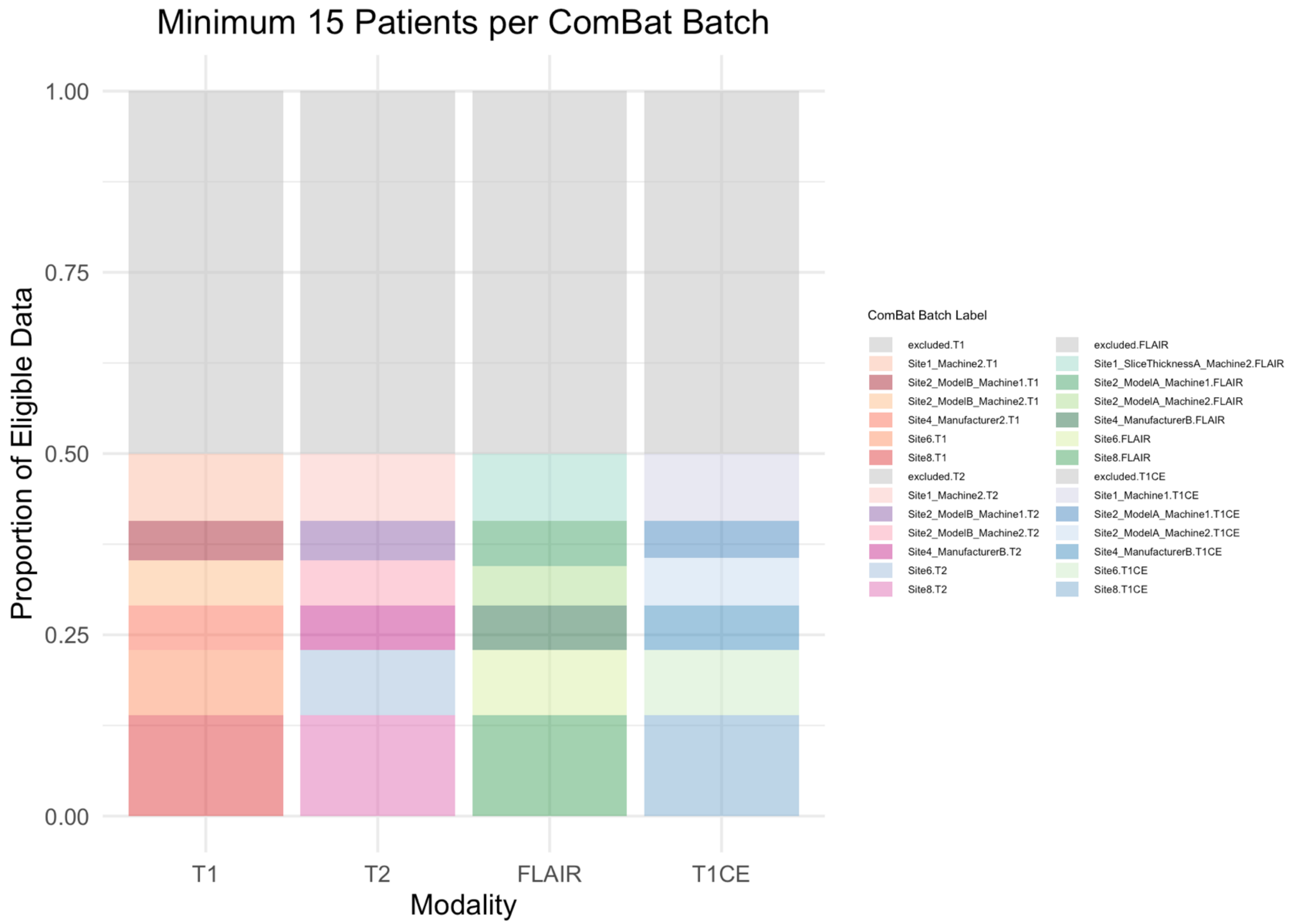

***Supplementary Figure 2a** – Calibration instability plots showing backwards feature selection clinical-radiomic combined models using bin count 32, different intensity standardisation techniques, with and without ComBat realignment and showing the effects of different ComBat batch sizes (5 and 15)*

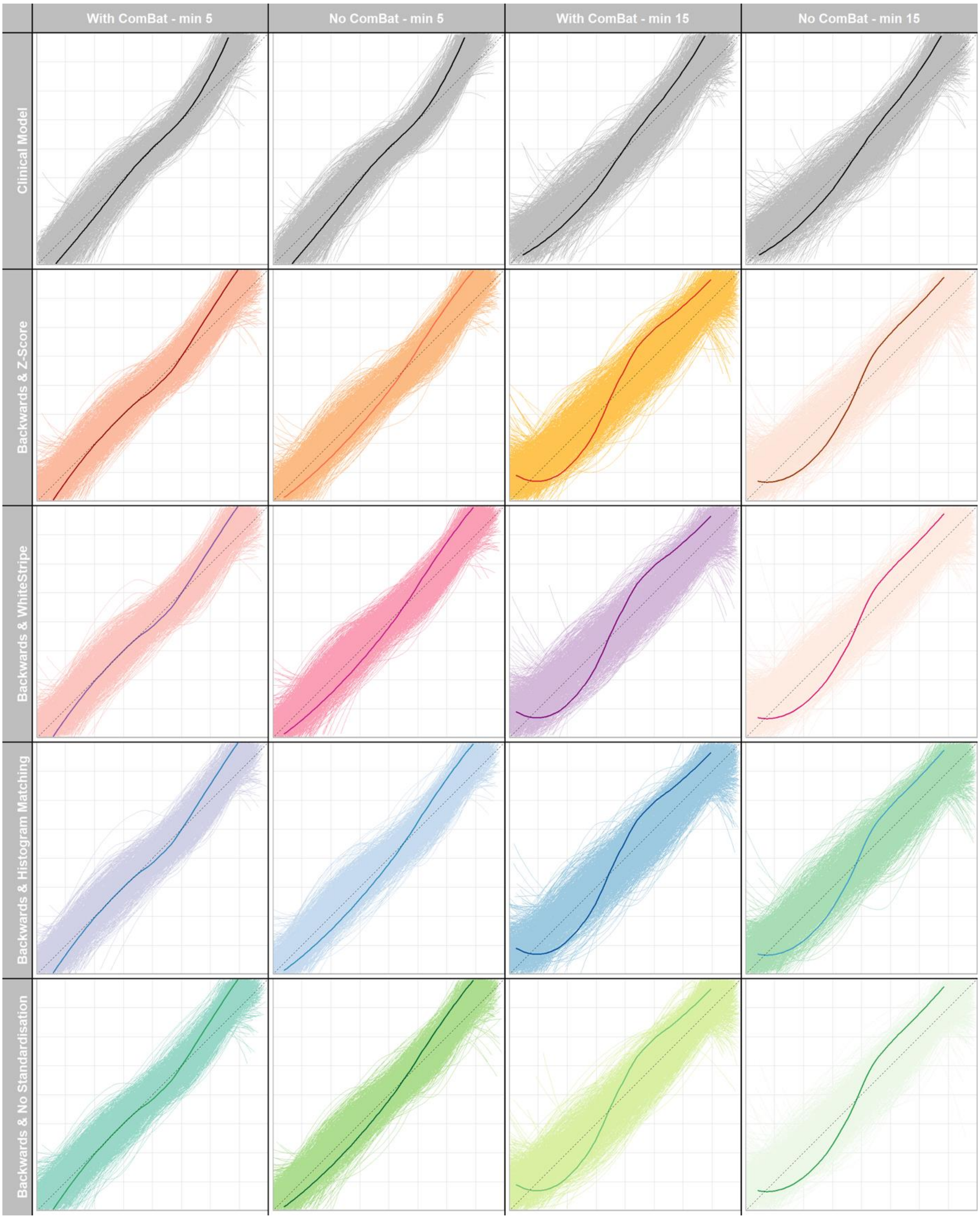

***Supplementary Figure 2b** – Calibration instability plots showing forwards feature selection clinical-radiomic combined models using bin count 32, different intensity standardisation techniques, with and without ComBat realignment and showing the effects of different ComBat batch sizes (5 and 15)*

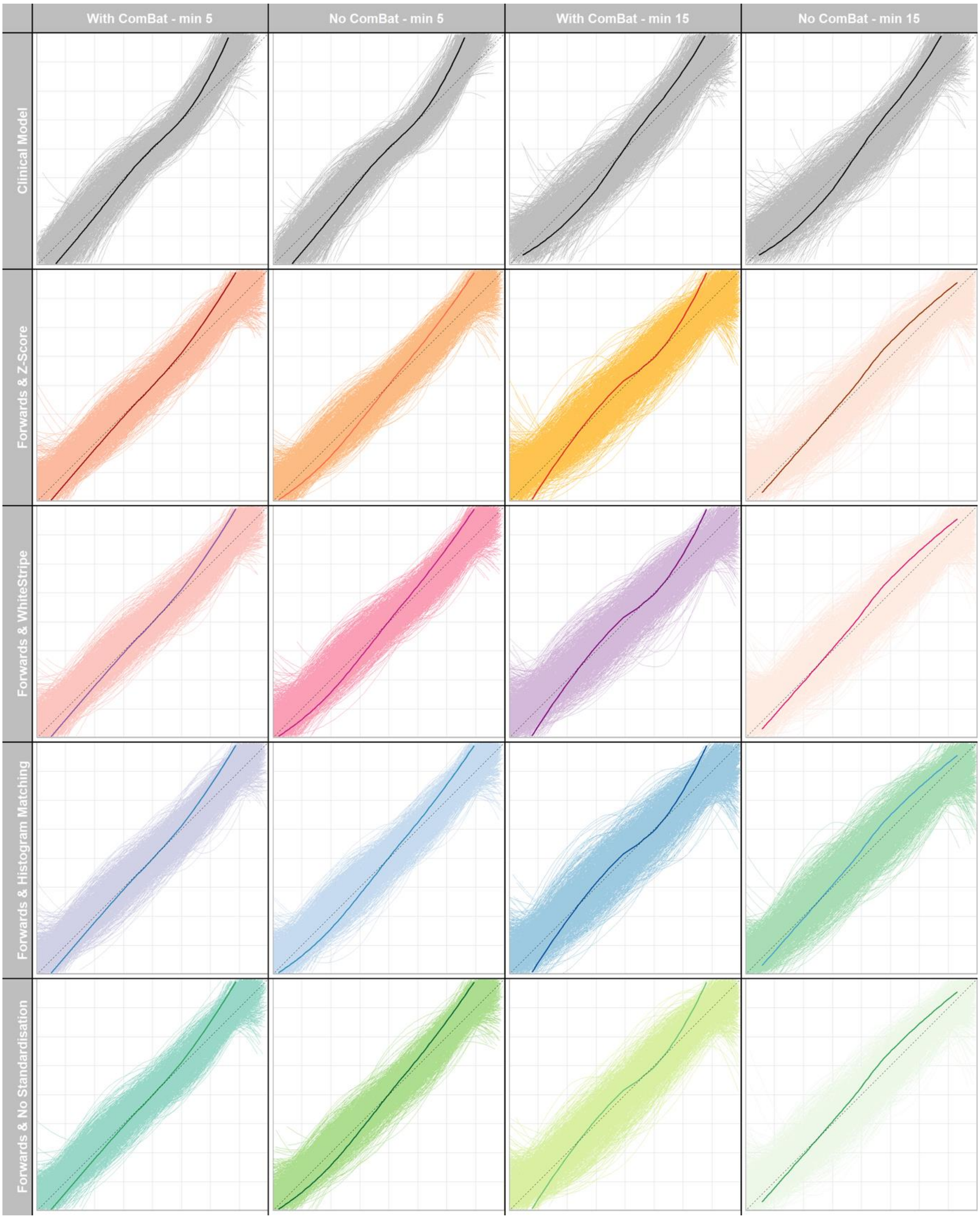

Supplementary Figure 2c *– Calibration instability plots showing Least Absolute Shrinkage and Selection Operator (LASSO) feature selection clinical-radiomic combined models using bin count 32, different intensity standardisation techniques, with and without ComBat realignment and showing the effects of different ComBat batch sizes (5 and 15)*

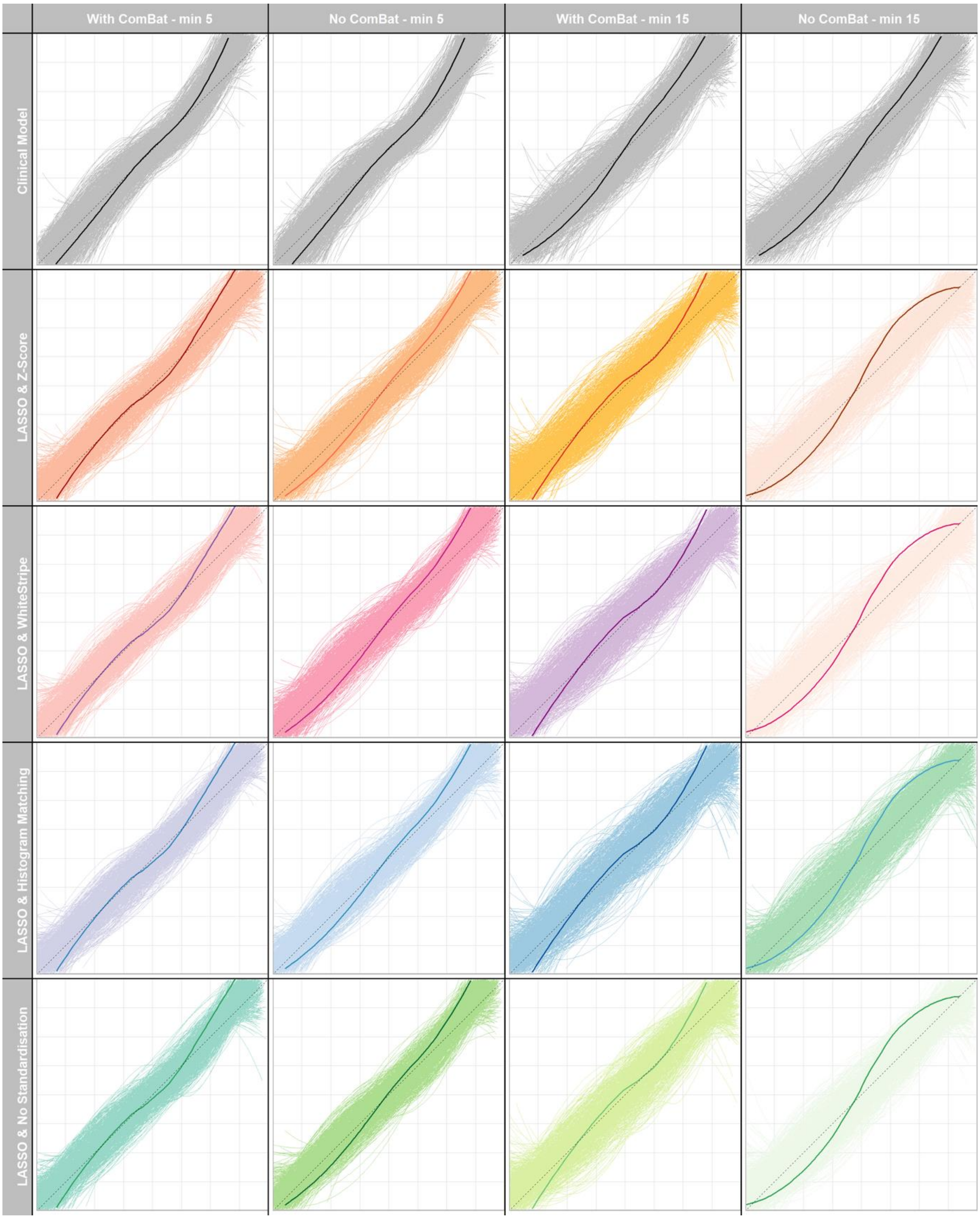

Supplementary Figure 2d *– Calibration instability plots showing random survival forests feature selection clinical-radiomic combined models using bin count 32, different intensity standardisation techniques, with and without ComBat realignment and showing the effects of different ComBat batch sizes (5 and 15)*

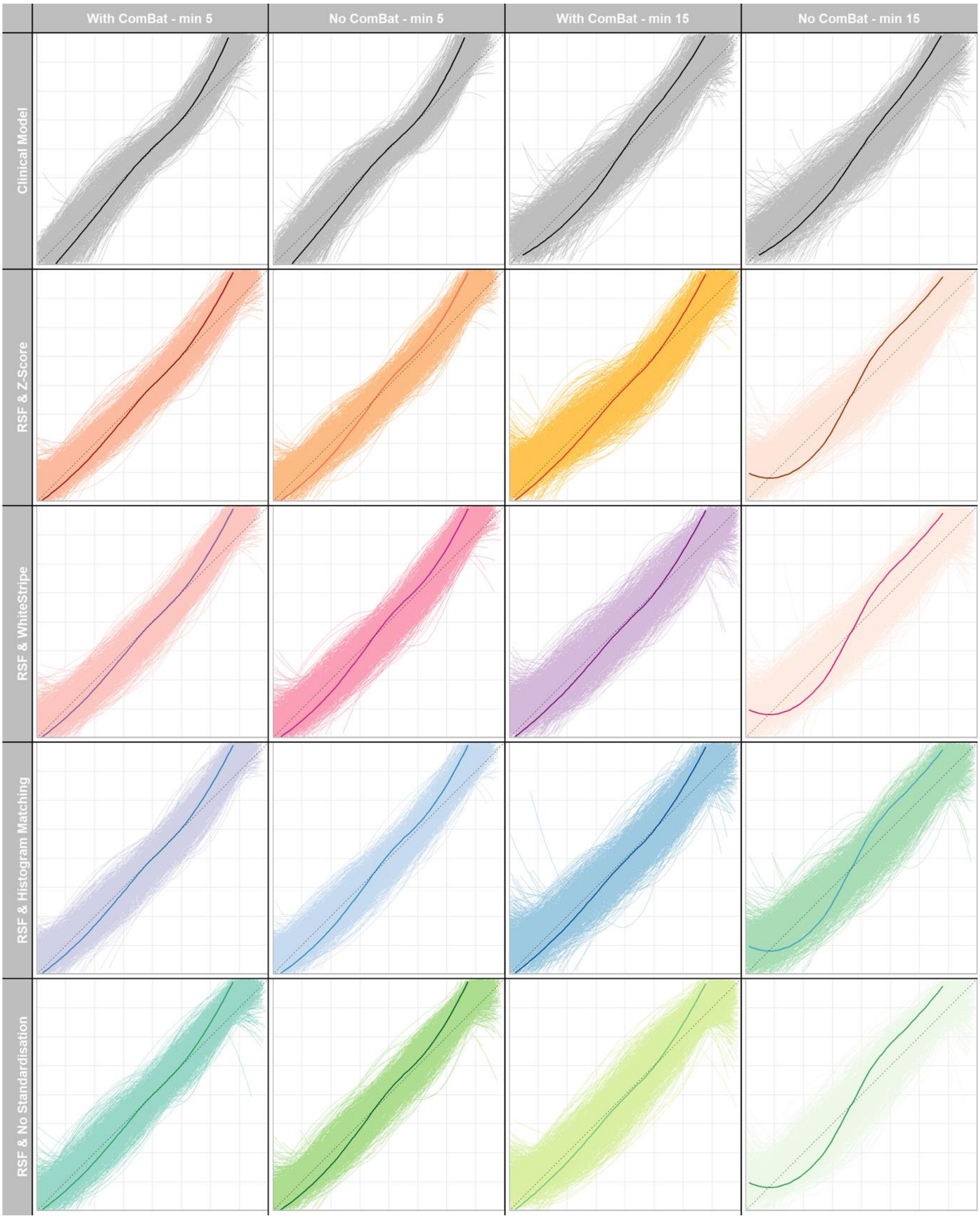

Supplement: Supplementary file 1 — ELECTRONIC SUPPLEMENTARY MATERIAL [file 330_2024_11168_MOESM1_ESM.pdf]
